# Photoinduced Photocatalyst-Free Cascade Cyclization of Alkynes with Sodium Sulfinates for the Synthesis of Benzothiophenes and Thioflavones

**DOI:** 10.3390/molecules28114436

**Published:** 2023-05-30

**Authors:** Hongqiang Dong, Chunli Chen, Jinlei Zhao, Yigang Ji, Wenchao Yang

**Affiliations:** 1 The Open Research Fund of the National and Local Joint Engineering Laboratory of High Efficiency and Superior-Quality Cultivation and Fruit Deep Processing Technology of Characteristic Fruit Trees in South Xinjiang, College of Agriculture, Tarim University, Alaer 843300, China; chenchunli1111@163.com; 2 National Engineering Research Center of Low-Carbon Processing and Utilization of Forest Biomass, Nanjing Forestry University, Nanjing 210037, China; hhuzhjl@163.com; 3 Jiangsu Surveying and Design Institute of Water Resources Co., Ltd., Yangzhou 225127, China; 4 Jiangsu Key Laboratory of Biofuctional Molecules, Department of Life Sciences and Chemistry, Jiangsu Second Normal University, Nanjing 210013, China; 5 Guangling College and School of Plant Protection, Yangzhou University, Yangzhou 225009, China

**Keywords:** sulfonylation, sodium sulfinates, radical, cyclization, alkynes

## Abstract

The subject of this investigation is a new method for the construction of sulfonylated heterocycles which overcomes the limitations of classical approaches using a cheap feedstock sulfonylating agent, especially under photocatalyst- and metal-free conditions.

## 1. Introduction

**Scheme 1:**
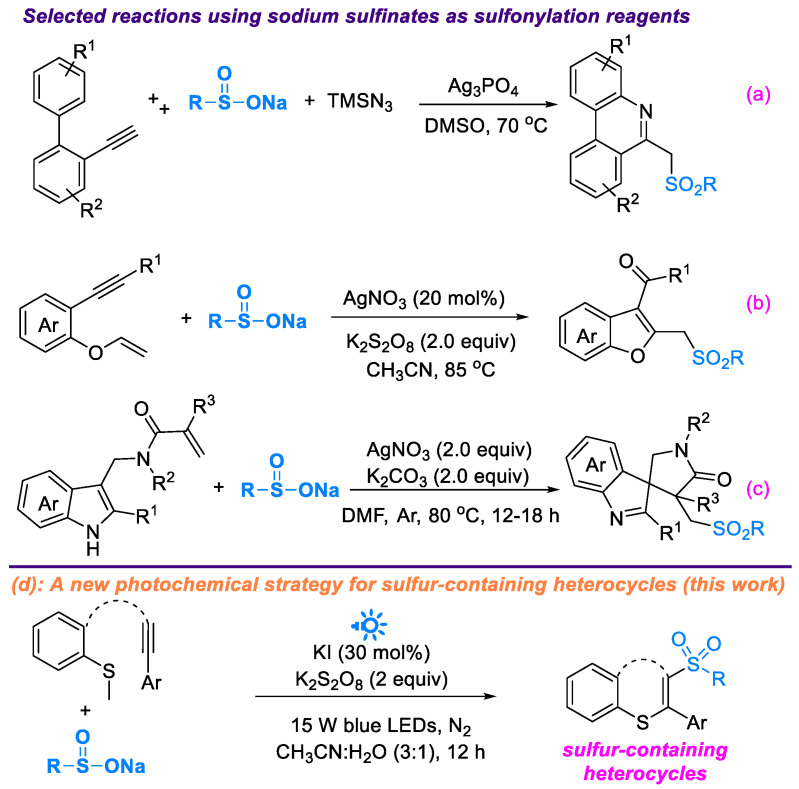
Selected reactions using sodium sulfinates as sulfonylation reagents (**a**–**c**) and our work (**d**).

Sulfonylated heterocycles are important motifs found in many natural products, agrochemicals, and pharmaceuticals, with special physiological and biological activities [1,2,3,4,5,6,7,8,9,10,11,12,13,14,15,16,17,18,19,20,21,22,23,24]. Generally, the oxidation of thioethers is the most popular approach to access sulfones [25,26]. However, in many cases, strong oxidation conditions are not applicable to all functional groups. As a result, developing the efficient and mild synthesis of value-added sulfonylated heterocycles has played a significant role in advancing heterocyclic chemistry, as well as accelerating the discovery of novel agrochemicals [27,28,29,30,31,32,33,34,35,36,37]. Sodium sulfinates, as a common and stable sulfonation raw material, are widely applied in the construction of organic sulfones, including the cascade reaction, direct C–H functionalization, and oxidative coupling, etc., [38,39,40,41]. Among these protocols, radical cascade cyclization of C–C unsaturated bonds has provided a powerful tool for the collection of sulfonylated heterocycles by introducing two different functional groups on the both ends of the alkynes or alkenes in one step [42,43,44,45,46,47,48]. From the point of synthetic chemistry, these radical cascade reactions can be very convenient and efficient to realize direct conversion from relatively inexpensive C–C unsaturated bonds to high-value-added, complex molecular scaffolds with abundant bioactivities. For example, Bi and co-workers realized a silver-catalyzed cascade cyclization of alkynes with sodium sulfinates for the synthesis of 6-methyl sulfonylated phenanthridines, where mechanistic studies indicate the transformation should proceed through an iminyl radical intermediate (Scheme 1a) [42]. Soon afterward, Wu and Jiang’s groups independently utilized sodium sulfinates and 1,6-enynes to achieve the sulfonylated benzofurans’ synthesis by using an AgNO_3_/K_2_S_2_O_8_ system (Scheme 1b) [43]. Very recently, another radical cascade spiro-cyclization of alkenes with sodium sulfinates for the direct synthesis of sulfonylated spiro[indole-3,3′-pyrrolidines] was reported by Wang’s group (Scheme 1c) [44]. Although significant progress has clearly been made in recent years, most of the traditional transformations usually depend on transition-metal catalysts or harsh reaction conditions. As a result, the development of a practical and green protocol to realize the radical cyclization of alkynes is still an attractive topic.

Over the past few decades, visible-light-induced organic reactions have emerged as an essential tool to construct new chemical bonds, featuring mild reaction conditions such as metal-free, room temperature, and simple operation conditions [49,50,51,52]. In 2015, we reported an example of using sulfinic acids as sulfonation reagents to react with alkyne in the presence of TBHP under visible-light irradiation [10]. However, the sulfonation reagent in this work is prepared from sodium sulfonates, leading to increase reaction steps. With our continuing interest in sustainable and photochemical chemistry [53,54,55,56,57,58], herein we disclose a photo-induced radical cascade cyclization of alkynes with sodium sulfinates for the divergent synthesis of sulfonated benzothiophenes and thioflavones under metal- and photocatalyst-free conditions (Scheme 1d).

## 2. Results and Discussion

Given these considerations, we set out to study this photoinduced cascade cyclization reaction by the treatment of 4-methylbenzenesulfinate and 2-alkynylthioanisoles in the presence of 15-W blue LED. Thankfully, the desired product benzothiophene **3a** was obtained with 82% yield using [Ir(dFCF_3_ppy)_2_dtbbpy]PF_6_ as a photocatalyst, KI as an additive, and K_2_S_2_O_8_ as an oxidant (Table 1, entry 1). After the evaluation of various additives such as NaI, NaBr, and KCl, KI was found to be the most effective to promote the reaction (Table 1, Entries 2–4). Then, other oxidants were further investigated, and the yields of the target products were lower than found using K_2_S_2_O_8_ (Table 1, Entries 5–8). We then examined the effect of other photocatalysts to this reaction, including 4CzIPN, 4CzIPN-*^t^*Bu and Mes-Acr^+^ClO_4_ (Table 1, Entries 9–12). It was found that 4CzIPN, 4CzIPN-*^t^*Bu, and Mes-Acr^+^ClO_4_ exhibited a lower catalytic activity than [Ir(dFCF_3_ppy)_2_dtbbpy]PF_6_ and, to our surprise, the sulfonated benzothiophenes could be obtained with considerable yield in the absence of a photocatalyst. This result shows that a photocatalyst is not essential for this reaction system. Finally, no desired product was observed without the irradiation of 15-W blue LED.

**Scheme 2:**
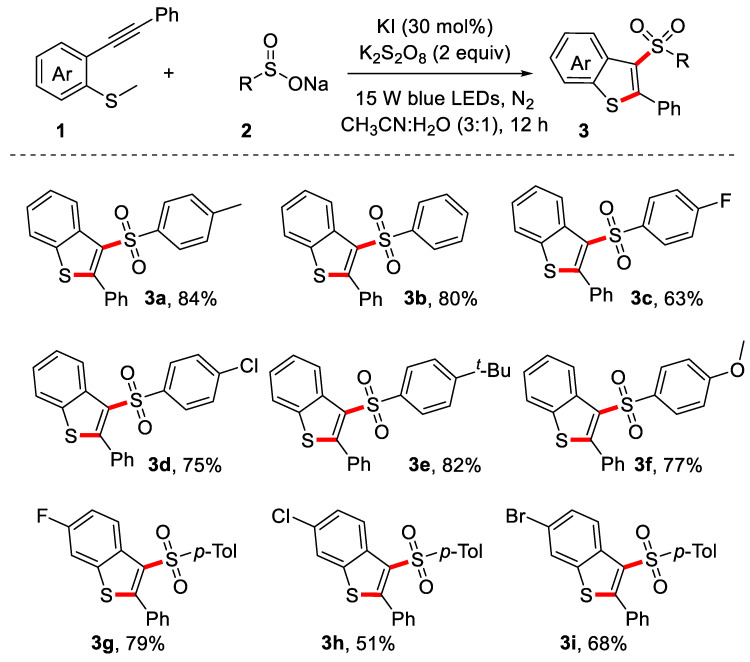
Substrate scope of sodium sulfinates: **1** (0.20 mmol), sodium sulfinates (**2**, 2.0 equiv), KI (30 mol%), K_2_S_2_O_8_ (2 equiv), CH_3_CN/H_2_O (3:1, 2.0 mL), N_2_, 15-W blue LED at room temperature for 12 h; isolated yield of the product based on **1**, reaction progress is monitored through TLC.

After the standard reaction condition was optimized, we tested a variety of sodium sulfinates to explore the reaction scope, and the results are summarized in Scheme 2. The sodium sulfinates with electron-donating and electron-withdrawing groups on the phenyl ring proceeded through this reaction smoothly. For example, sodium sulfinates containing a halogen group (F or Cl) were all favorable, affording the corresponding products with 63% and 75% yields. In addition, sodium sulfinates containing OMe or OEt were also suitable substrates. As shown in Scheme 2, when sodium sulfinates containing a substituent group on the Ar ring were employed for this transformation, 51–79% yields of the products (**3g**–**3i**) were obtained.

**Scheme 3:**
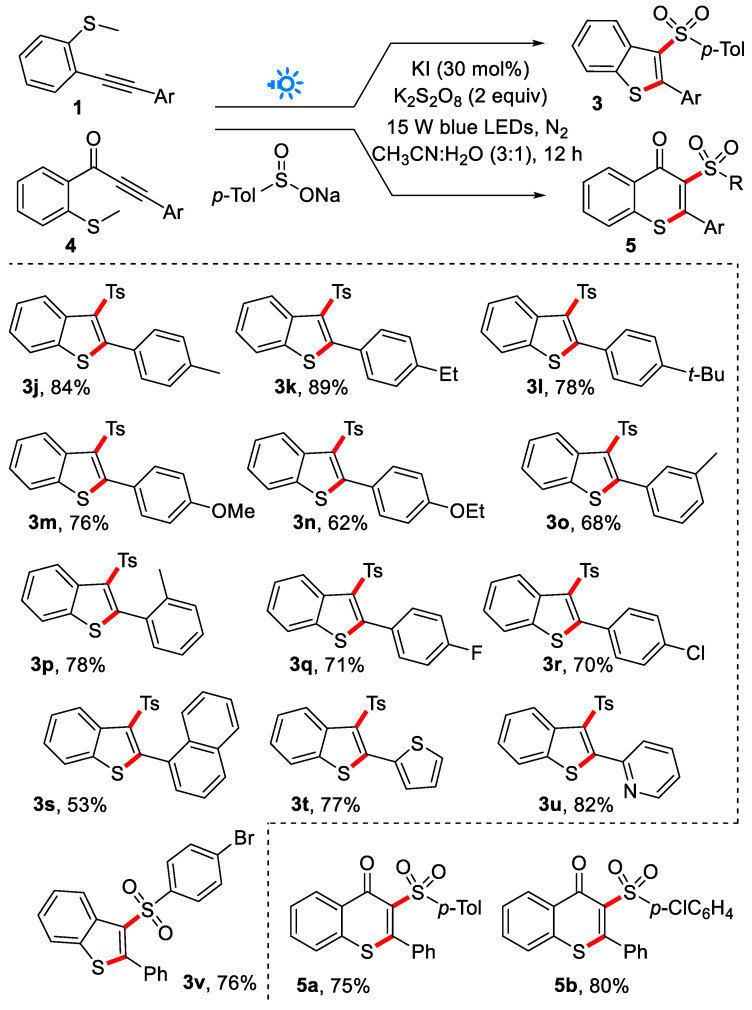
Substrate scope of 2-alkynylthioanisoles: Unless otherwise noted, all reactions were performed with **1** (0.20 mmol), sodium sulfinates (**2**, 2.0 equiv), KI (30 mol%), K_2_S_2_O_8_ (2 equiv), CH_3_CN/H_2_O (3:1, 2.0 mL), N_2_, 15-W blue LED at room temperature for 12 h; isolated yield of the product based on **1**, reaction progress is monitored through TLC.

In order to further prove the practicability and efficiency of the photochemical approach, we further expand the substrate scope on 2-alkynylthioanisoles. As shown in Scheme 3, a variety of substituted 2-alkynylthioanisoles could react with 4-methylbenzenesulfinate to produce the corresponding sulfonated benzothiophenes (**3j**–**3u**) with 53–89% yields. The 2-alkynylthioanisoles bearing strong electron-donating groups on the Ar ring were compatible, affording the corresponding products (**3m** and **3n**) with 76% and 62% yields. Moreover, substrates possessing a heteroaromatic ring, such as pyridine and thiophene, could also undergo the reaction smoothly, generating the corresponding products with reasonable yields (**3t** and **3u**). There is no doubt that the bromine-substituted sodium sulfinate could be successfully transformed into the corresponding product **3v**, which will allow further complex molecule synthesis via cross-coupling reactions. We next examined the alkynes systems to evaluate their applicability to this transformation, synthesizing and applying aryl ynones as cascade substrates to provide sulfonated thioflavones (**5a**–**5b**) with good yields.

**Scheme 4:**
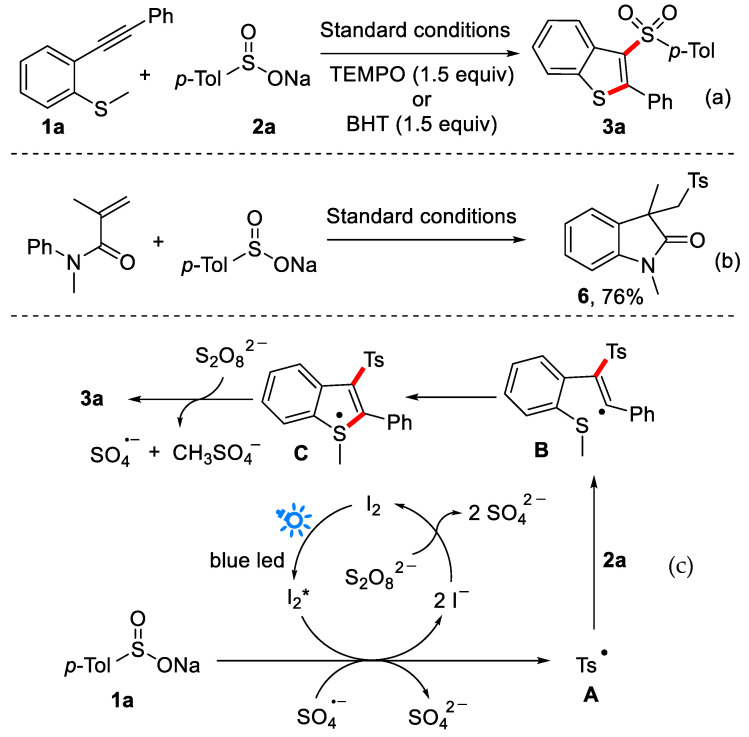
The controlled experiments (**a**,**b**) and proposed mechanism (**c**).

In order to elucidate the reaction mechanism of this reaction, a free-radical inhibition experiment was conducted, in which stoichiometric amounts of radical scavengers were added and the reaction was fully suppressed (Scheme 4a). In addition, a well-known free radical accepter *N*-arylacrylamide, which was widely used in radical tandem reactions, was applied instead of 2-alkynylthioanisoles to give sulfonylated oxindole at a 76% yield (Scheme 4b). These results indicated that the free radical process may be involved in this transformation. A plausible reaction mechanism is outlined in Scheme 4 based on the above observations and previous reports [59]. Initially, I_2_ is generated from KI by the oxidation of K_2_S_2_O_8_, which would then transfer to the excited state I_2_* with the irradiation of 15-W blue LEDs. Subsequently, the reaction between I_2_*, SO_4_^•–^, and 4-methylbenzenesulfinate produces a sulfonyl radical, followed by the radical addition of the sulfonyl radical with alkyne in **1a** to give a vinyl radical intermediate. After that, the addition of a vinyl radical to the sulfur atom produces the intermediate **C**. Finally, the corresponding cascade product **3a** is obtained via oxidation and demethylation.

## 3. Materials and Methods

### 3.1. General Information

All reactions were carried out under a nitrogen atmosphere. ^1^H NMR ^13^C NMR, and ^19^F NMR spectra were measured on a Bruker Avance NMR spectrometer (600 MHz/151 MHz/565 NMR) in CDCl_3_ as solvent and recorded in ppm relative to the internal tetramethylsilane standard. ^1^H NMR data are reported as follows: δ, chemical shift; coupling constants (*J* are given in Hertz, Hz) and integration. Abbreviations to denote the multiplicity of a particular signal are s (singlet), d (doublet), t (triplet), q (quartet), dd (doublet of doublets), and m (multiplet). The 15-W blue LED was purchased from https://item.taobao.com/item.htm?id=524749100016&ali_refid=a3_430673_1006:1121803562:N:FxDVk1sg3f08W8u%2BfdnZUtGvFtTT9lsR:af40c0c0536da02c91473a14c4e25edc&ali_trackid=1_af40c0c0536da02c91473a14c4e25edc&spm=a2e0b.20350158.31919782.21&mt= (accessed on 15 May 2023). Due to the fact that the target compound is known, mass spectrometry analysis for compound characterization was not conducted in the article.

### 3.2. Preparation of the Starting Materials

The 2-alkynylthioanisoles (**1a**) derivatives were prepared according to the reported method [56,60]. The solvents and oxidants including DMF, THF, K_2_S_2_O_8_, DTBP, etc., were purchased from commercial suppliers including Bidepharm (Shanghai, China); functionalized anilines, photocatalysts, and functionalized aryl sulfonyl chlorides were purchased from Energy Chemical (Shanghai, China); petroleum ether and ethyl acetate were purchased from Shanghai Chemical Company (Shanghai, China). All solvents were dried and freshly distilled in N_2_ prior to use. Products were purified by flash chromatography on a 200–300 mesh silica gel.

### 3.3. General Procedure for the Synthesis of ***3a***

A dry 15-mL tube was charged with 2-alkynylthioanisole (**1a**, 0.20 mmol), sodium sulfinates (**2a**, 0.40 mmol), CH_3_CN:H_2_O (3:1, 2 mL), KI (30 mol%), K_2_S_2_O_8_ (2 equiv), and a magnetic stir bar. Then, the mixture was reacted under a 15-W blue LED light at room temperature and a nitrogen atmosphere for 12 hours. After the reaction, the mixture was concentrated to obtain the crude product, and the crude product was further purified by rapid chromatography (silica gel, petroleum ether (PE)/ethyl acetate (EA) = 30/1 to 15/1) to obtain the required product **3a**. The 1H NMR, 13C NMR and 19F NMR spectra of the products can be found in the Supplementary Materials.

*Product* **3a: *2-phenyl-3-tosylbenzo[b]thiophene*.** The sulfonylation product was purified by flash column chromatography on silica gel (PE/AcOEt: 30/1–15/1) to afford **3a** as a white solid (58 mg, 84% yield). ^1^H NMR (600 MHz, CDCl_3_**:** δ 8.62 (d, J = 8.4 Hz, 1H), 7.76 (d, J = 8.1 Hz, 1H), 7.53 (d, J = 8.3 Hz, 2H), 7.52–7.48 (m, 1H), 7.47–7.43 (m, 1H), 7.43–7.37 (m, 5H), 7.10 (d, J = 8.1 Hz, 2H), 2.30 (s, 3H). ^13^C NMR (151 MHz, CDCl_3_)**:** δ 152.5, 143.9, 139.4, 138.2, 136.1, 131.7, 130.5, 130.3, 129.5, 129.4, 127.6, 127.0, 125.9, 125.6, 124.6, 121.8, 21.5.

*Product* **3b: *2-phenyl-3-(phenylsulfonyl)benzo[b]thiophene***. The sulfonylation product was purified by flash column chromatography on silica gel (PE/AcOEt: 30/1–15/1) to afford the **3b** as a white solid (56 mg, 80% yield). ^1^H NMR (600 MHz, CDCl_3_)**:** δ 8.64 (d, J = 8.4 Hz, 1H), 7.80 (d, J = 8.0 Hz, 1H), 7.64 (dd, J = 8.4, 1.1 Hz, 2H), 7.55–7.51 (m, 1H), 7.48–7.43 (m, 3H), 7.43–7.39 (m, 4H), 7.33 (t, J = 7.9 Hz, 2H). ^13^C NMR (151 MHz, CDCl_3_)**:** δ 152.9, 142.3, 138.1, 136.2, 132.9, 131.6, 130.5, 130.0, 129.5, 128.7, 127.7, 126.9, 126.0, 125.6, 124.6, 121.7.

*Product* **3c: *3-((4-fluorophenyl)sulfonyl)-2-phenylbenzo[b]thiophene***. The sulfonylation product was purified by flash column chromatography on silica gel (PE/AcOEt: 30/1–15/1) to afford **3c** as a yellow solid (46 mg, 63% yield). ^1^H NMR (600 MHz, CDCl_3_)**:** δ 8.65 (d, J = 8.3 Hz, 1H), 7.81 (d, J = 8.0 Hz, 1H), 7.64–7.59 (m, 2H), 7.56–7.51 (m, 1H), 7.49–7.43 (m, 2H), 7.42–7.38 (m, 4H), 7.01–6.94 (m, 2H). ^13^C NMR (151 MHz, CDCl_3_)**:** δ 166.0, 164.3, 152.8, 138.3 (d, J = 3.1 Hz), 138.1, 136.0, 131.4, 130.5, 130.0, 129.8, 129.7 (d, J = 22.7 Hz), 126.9 (d, J = 252.1 Hz), 125.7, 124.5, 121.8, 115.9 (d, J = 22.8 Hz).

*Product* **3d: *3-((4-chlorophenyl)sulfonyl)-2-phenylbenzo[b]thiophene***. The sulfonylation product was purified by flash column chromatography on silica gel (PE/AcOEt: 30/1–15/1) to afford **3d** as a yellow solid (57 mg, 75% yield). ^1^H NMR (600 MHz, CDCl_3_)**:** δ 8.63 (d, J = 8.4 Hz, 1H), 7.82 (d, J = 8.1 Hz, 1H), 7.54 (t, J = 8.9 Hz, 3H), 7.50–7.44 (m, 2H), 7.43–7.38 (m, 4H), 7.28 (d, J = 8.6 Hz, 2H). ^13^C NMR (151 MHz, CDCl_3_)**:** δ 153.1, 140.7, 139.5, 138.1, 136.0, 131.4, 130.5, 129.7, 129.6, 129.0, 128.4, 127.7, 126.1, 125.7, 124.5, 121.8.

*Product* **3e: *3-((4-(tert-butyl)phenyl)sulfonyl)-2-phenylbenzo[b]thiophene***. The sulfonylation product was purified by flash column chromatography on silica gel (PE/AcOEt: 30/1–15/1) to afford **3e** as a white solid (66 mg, 82% yield). ^1^H NMR (600 MHz, CDCl_3_)**:** δ 8.65 (d, J = 8.4 Hz, 1H), 7.81 (d, J = 8.1 Hz, 1H), 7.58 (d, J = 8.6 Hz, 2H), 7.55–7.52 (m, 1H), 7.47–7.43 (m, 2H), 7.43–7.38 (m, 4H), 7.33 (d, J = 8.6 Hz, 2H), 1.26 (s, 9H). ^13^C NMR (151 MHz, CDCl_3_)**:** δ 156.7, 152.4, 139.2, 138.1, 136.2, 131.7, 130.4, 129.4, 127.6, 126.9, 125.9, 125.7, 125.5, 124.7, 121.7, 35.1, 31.0.

*Product* **3f: *3-((4-methoxyphenyl)sulfonyl)-2-phenylbenzo[b]thiophene***. The sulfonylation product was purified by flash column chromatography on silica gel (PE/AcOEt: 15/1–7/1) to afford **3f** as a white solid (58 mg, 77% yield). ^1^H NMR (600 MHz, CDCl_3_)**:** δ 8.56 (d, J = 8.4 Hz, 1H), 7.73 (d, J = 8.1 Hz, 1H), 7.50 (d, J = 9.0 Hz, 2H), 7.47–7.43 (m, 1H), 7.41–7.38 (m, 1H), 7.37–7.33 (m, 5H), 6.74–6.69 (m, 2H), 3.71 (s, 3H).^13^C NMR (151 MHz, CDCl_3_)**:** δ 162.1, 151.0, 137.1, 135.0, 133.0, 130.7, 129.7, 129.4, 128.3, 128.2, 126.6, 124.8, 124.5, 123.6, 120.7, 112.9, 54.5.

*Product* **3g: *6-fluoro-2-phenyl-3-tosylbenzo[b]thiophene***. The sulfonylation product was purified by flash column chromatography on silica gel (PE/AcOEt: 30/1–15/1) to afford **3g** as a brown solid (60 mg, 79% yield). ^1^H NMR (600 MHz, CDCl_3_): δ 8.61 (dd, J = 9.2, 5.1 Hz, 1H), 7.50 (d, J = 8.3 Hz, 2H), 7.48–7.44 (m, 2H), 7.41–7.38 (m, 4H), 7.29–7.24 (m, 1H), 7.12 (d, J = 8.2 Hz, 2H), 2.33 (s, 3H). ^13^C NMR (151 MHz, CDCl_3_): δ 160.8 (d, J = 247.7 Hz), 152.0 (d, J = 3.3 Hz), 144.0, 139.2 (d, J = 4.4 Hz), 139.1, 132.6, 131.3, 130.5, 130.1, 129.6, 129.4, 127.7, 127.0, 126.1 (d, J = 8.9 Hz), 114.9 (d, J = 23.9 Hz), 107.9 (d, J = 25.4 Hz), 21.5.

*Product* **3h: *6-chloro-2-phenyl-3-tosylbenzo[b]thiophene.*** The sulfonylation product was purified by flash column chromatography on silica gel (PE/AcOEt: 30/1–15/1) to afford **3h** as a white solid (40 mg, 51% yield). ^1^H NMR (600 MHz, CDCl_3_): δ 8.57 (d, J = 8.9 Hz, 1H), 7.78 (d, J = 1.9 Hz, 1H), 7.50–7.46 (m, 4H), 7.40 (d, J = 4.4 Hz, 4H), 7.12 (d, J = 8.2 Hz, 2H), 2.33 (s, 3H). ^13^C NMR (151 MHz, CDCl_3_): δ 152.7, 144.1, 139.1, 139.1, 134.6, 131.9, 131.2, 130.5, 130.3, 129.6, 129.4, 127.7, 127.0, 126.8, 125.6, 121.3, 21.5.

*Product* **3i***: **6-bromo-2-phenyl-3-tosylbenzo[b]thiophene**.* The sulfonylation product was purified by flash column chromatography on silica gel (PE/AcOEt: 30/1–15/1) to afford **3i** as a yellow solid (60 mg, 68% yield). ^1^H NMR (600 MHz, CDCl_3_): δ 8.51 (d, J = 8.9 Hz, 1H), 7.94 (d, J = 1.8 Hz, 1H), 7.62 (dd, J = 8.9, 1.8 Hz, 1H), 7.48 (t, J = 7.0 Hz, 3H), 7.40 (d, J = 4.4 Hz, 4H), 7.12 (d, J = 8.2 Hz, 2H), 2.33 (s, 3H). ^13^C NMR (151 MHz, CDCl_3_): δ 152.7, 144.1, 139.4, 139.1, 135.0, 131.1, 130.5, 130.3, 129.7, 129.4, 129.4, 127.7, 127.0, 125.8, 124.2, 119.7, 21.5.

*Product* **3j: *2-(p-tolyl)-3-tosylbenzo[b]thiophene***. The sulfonylation product was purified by flash column chromatography on silica gel (PE/AcOEt: 30/1–15/1) to afford **3j** as a white solid (63 mg, 84% yield). ^1^H NMR (600 MHz, CDCl_3_): δ 8.59 (d, J = 8.4 Hz, 1H), 7.76 (d, J = 8.0 Hz, 1H), 7.56 (d, J = 8.3 Hz, 2H), 7.51–7.47 (m, 1H), 7.42–7.37 (m, 1H), 7.33 (d, J = 8.0 Hz, 2H), 7.21 (d, J = 7.9 Hz, 2H), 7.12 (d, J = 8.2 Hz, 2H), 2.43 (s, 3H), 2.31 (s, 3H). ^13^C NMR (151 MHz, CDCl_3_): δ 153.0, 143.8, 139.6, 139.5, 138.1, 136.2, 130.4, 129.9, 129.4, 128.7, 128.4, 127.0, 125.8, 125.5, 124.5, 121.7, 21.5, 21.5.

*Product* **3k: *2-(4-ethylphenyl)-3-tosylbenzo[b]thiophene***. The sulfonylation product was purified by flash column chromatography on silica gel (PE/AcOEt: 30/1–15/1) to afford **3k** as a white solid (69 mg, 89% yield). ^1^H NMR (600 MHz, CDCl_3_): δ 8.61 (d, J = 8.4 Hz, 1H), 7.74 (d, J = 8.1 Hz, 1H), 7.54 (d, J = 8.3 Hz, 2H), 7.50–7.46 (m, 1H), 7.40–7.36 (m, 1H), 7.34 (d, J = 8.1 Hz, 2H), 7.22 (d, J = 8.0 Hz, 2H), 7.09 (d, J = 8.2 Hz, 2H), 2.71 (q, J = 7.6 Hz, 2H), 2.29 (s, 3H), 1.29 (t, J = 7.6 Hz, 3H). ^13^C NMR (151 MHz, CDCl_3_): δ 152.9, 145.8, 143.8, 139.5, 138.1, 136.2, 130.5, 130.0, 129.4, 128.9, 127.2, 127.0, 125.8, 125.5, 124.6, 121.7, 28.8, 21.5, 15.4.

*Product* **3l: *2-(4-(tert-butyl)phenyl)-3-tosylbenzo[b]thiophene***. The sulfonylation product was purified by flash column chromatography on silica gel (PE/AcOEt: 30/1–15/1) to afford **3l** as a white solid (65 mg, 78% yield). ^1^H NMR (600 MHz, CDCl_3_): δ 8.63 (d, J = 8.3 Hz, 1H), 7.78 (d, J = 8.0 Hz, 1H), 7.52–7.48 (m, 3H), 7.43–7.37 (m, 3H), 7.36–7.32 (m, 2H), 7.08 (d, J = 8.1 Hz, 2H), 2.31 (s, 3H), 1.38 (s, 9H). ^13^C NMR (151 MHz, CDCl_3_): δ 152.7, 152.6, 143.6, 139.4, 138.1, 136.3, 130.2, 130.0, 129.2, 128.6, 127.1, 125.8, 125.4, 124.6, 124.6, 121.7, 34.8, 31.3, 21.5.

*Product* **3m: *2-(4-methoxyphenyl)-3-tosylbenzo[b]thiophene***. The sulfonylation product was purified by flash column chromatography on silica gel (PE/AcOEt: 15/1–7/1) to afford **3m** as a white solid (59 mg, 76% yield). ^1^H NMR (600 MHz, CDCl_3_): δ 8.60 (d, J = 8.4 Hz, 1H), 7.77 (d, J = 8.0 Hz, 1H), 7.54 (d, J = 8.3 Hz, 2H), 7.52–7.47 (m, 1H), 7.42–7.36 (m, 3H), 7.12 (d, J = 8.2 Hz, 2H), 6.93 (d, J = 8.7 Hz, 2H), 3.88 (s, 3H), 2.32 (s, 3H). ^13^C NMR (151 MHz, CDCl_3_): δ 160.6, 152.8, 143.8, 139.5, 138.0, 136.3, 131.9, 129.8, 129.4, 126.9, 125.8, 125.4, 124.5, 123.7, 121.7, 113.1, 55.4, 21.5.

*Product* **3n: *2-(4-ethoxyphenyl)-3-tosylbenzo[b]thiophene***. The sulfonylation product was purified by flash column chromatography on silica gel (PE/AcOEt: 15/1–7/1) to afford **3n** as a white solid (50 mg, 62% yield). ^1^H NMR (600 MHz, CDCl_3_): δ 8.61 (d, J = 8.3 Hz, 1H), 7.77 (d, J = 8.0 Hz, 1H), 7.54 (d, J = 8.3 Hz, 2H), 7.52–7.47 (m, 1H), 7.42–7.38 (m, 1H), 7.38–7.34 (m, 2H), 7.12 (d, J = 8.1 Hz, 2H), 6.94–6.89 (m, 2H), 4.10 (q, J = 7.0 Hz, 2H), 2.32 (s, 3H), 1.46 (t, J = 7.0 Hz, 3H). ^13^C NMR (151 MHz, CDCl_3_): δ 160.0, 153.0, 143.7, 139.6, 138.0, 136.3, 131.9, 129.7, 129.3, 126.9, 125.8, 125.4, 124.5, 123.5, 121.6, 113.6, 63.6, 21.5, 14.8.

*Product* **3o***: **2-(m-tolyl)-3-tosylbenzo[b]thiophene**.* The sulfonylation product was purified by flash column chromatography on silica gel (PE/AcOEt: 30/1–15/1) to afford **3o** as a white solid (51 mg, 68% yield). ^1^H NMR (600 MHz, CDCl_3_): δ 8.63 (d, J = 8.4 Hz, 1H), 7.79 (d, J = 8.1 Hz, 1H), 7.55 (d, J = 8.3 Hz, 2H), 7.54–7.50 (m, 1H), 7.45–7.40 (m, 1H), 7.29 (dd, J = 14.9, 7.5 Hz, 2H), 7.23 (d, J = 7.3 Hz, 1H), 7.17–7.11 (m, 3H), 2.38 (s, 3H), 2.34 (s, 3H). ^13^C NMR (151 MHz, CDCl_3_): δ 152.7, 143.8, 139.5, 138.1, 137.2, 136.2, 131.5, 131.0, 130.2, 130.1, 129.3, 127.5, 127.1, 125.8, 125.5, 124.6, 121.7, 21.5, 21.3.

*Product* **3p: *2-(o-tolyl)-3-tosylbenzo[b]thiophene***. The sulfonylation product was purified by flash column chromatography on silica gel (PE/AcOEt: 30/1–15/1) to afford **3p** as a white solid (58 mg, 78% yield). ^1^H NMR (600 MHz, CDCl_3_): δ 8.66 (d, J = 8.3 Hz, 1H), 7.81 (d, J = 8.1 Hz, 1H), 7.54 (t, J = 8.4 Hz, 3H), 7.47–7.42 (m, 1H), 7.37 (td, J = 7.6, 1.1 Hz, 1H), 7.22 (dd, J = 14.7, 7.2 Hz, 2H), 7.17–7.12 (m, 3H), 2.35 (s, 3H), 2.08 (s, 3H). ^13^C NMR (151 MHz, CDCl_3_): δ 151.6, 144.0, 139.2, 138.5, 138.1, 135.8, 131.2, 131.0, 130.2, 129.8, 129.6, 129.4, 127.3, 125.8, 125.5, 124.9, 124.5, 121.8, 21.5, 20.2.

*Product* **3q: *2-(4-fluorophenyl)-3-tosylbenzo[b]thiophene***. The sulfonylation product was purified by flash column chromatography on silica gel (PE/AcOEt: 30/1–15/1) to afford **3q** as a yellow solid (54 mg, 71% yield). ^1^H NMR (600 MHz, CDCl_3_): δ 8.61 (d, J = 8.4 Hz, 1H), 7.80 (d, J = 8.1 Hz, 1H), 7.53 (t, J = 8.0 Hz, 3H), 7.43 (m, J = 8.6, 6.7, 1.4 Hz, 3H), 7.15 (d, J = 8.2 Hz, 2H), 7.10 (t, J = 8.6 Hz, 2H), 2.34 (s, 3H). ^13^C NMR (151 MHz, CDCl_3_): δ 162.7, 151.2, 144.0, 139.3, 138.0, 136.0, 132.4 (d, J = 8.4 Hz), 130.6, 129.4, 127.6, 126.9, 126.0, 125.7, 124.6, 121.7, 114.8 (d, J = 21.9 Hz), 21.5.

*Product* **3r: *2-(4-chlorophenyl)-3-tosylbenzo[b]thiophene***. The sulfonylation product was purified by flash column chromatography on silica gel (PE/AcOEt: 30/1–15/1) to afford **3r** as a yellow solid (55 mg, 70% yield). ^1^H NMR (600 MHz, CDCl_3_): δ 8.60 (d, J = 8.4 Hz, 1H), 7.80 (d, J = 8.0 Hz, 1H), 7.55 (t, J = 5.8 Hz, 2H), 7.54–7.51 (m, 1H), 7.46–7.42 (m, 1H), 7.40–7.36 (m, 4H), 7.16 (d, J = 8.2 Hz, 2H), 2.35 (s, 3H). ^13^C NMR (151 MHz, CDCl_3_): δ 150.8, 144.1, 139.3, 138.1, 136.0, 135.8, 131.8, 130.7, 130.1, 129.5, 127.9, 127.0, 126.0, 125.8, 124.6, 121.7, 21.5.

*Product* **3s: *2-(naphthalen-1-yl)-3-tosylbenzo[b]thiophene***. The sulfonylation product was purified by flash column chromatography on silica gel (PE/AcOEt: 15/1–7/1) to afford **3s** as a yellow solid (43 mg, 53% yield). ^1^H NMR (600 MHz, CDCl_3_): δ 8.60 (d, J = 8.4 Hz, 1H), 7.75 (d, J = 8.0 Hz, 1H), 7.54 (d, J = 8.3 Hz, 2H), 7.51–7.46 (m, 1H), 7.38 (m, J = 8.7, 7.4, 1.6 Hz, 3H), 7.11 (d, J = 8.2 Hz, 2H), 6.94–6.90 (m, 2H), 3.86 (s, 3H), 2.30 (s, 3H). ^13^C NMR (151 MHz, CDCl_3_): δ 160.6, 152.9, 143.8, 139.6, 138.0, 136.3, 131.9, 131.4, 130.4, 129.8, 129.4, 126.9, 125.8, 125.4, 124.5, 123.7, 121.7, 113.2, 55.4, 53.5, 31.4, 30.2, 21.5.

*Product* **3t: *2-(thiophen-2-yl)-3-tosylbenzo[b]thiophene***. The sulfonylation product was purified by flash column chromatography on silica gel (PE/AcOEt: 15/1–5/1) to afford **3t** as a yellow solid (56 mg, 77% yield). ^1^H NMR (600 MHz, CDCl_3_): δ 8.67 (d, J = 8.4 Hz, 1H), 7.77 (d, J = 8.1 Hz, 1H), 7.59 (d, J = 8.3 Hz, 2H), 7.53–7.49 (m, 3H), 7.44–7.40 (m, 1H), 7.17–7.11 (m, 3H), 2.33 (s, 3H). ^13^C NMR (151 MHz, CDCl_3_): δ 144.6, 143.9, 139.1, 138.2, 136.5, 132.2, 131.0, 130.7, 129.4, 129.3, 127.4, 126.9, 126.0, 125.8, 124.8, 121.5, 21.5.

*Product* **3u: *2-(3-tosylbenzo[b]thiophen-2-yl)pyridine***. The sulfonylation product was purified by flash column chromatography on silica gel (PE/AcOEt: 15/1–5/1) to afford **3u** as a white solid (59 mg, 82% yield). ^1^H NMR (600 MHz, CDCl_3_): δ 8.71–8.68 (m, 1H), 8.45 (d, J = 8.3 Hz, 1H), 7.85–7.79 (m, 5H), 7.51–7.46 (m, 1H), 7.43–7.40 (m, 1H), 7.40–7.37 (m, 1H), 7.20 (d, J = 8.2 Hz, 2H), 2.32 (s, 3H). ^13^C NMR (151 MHz, CDCl_3_): δ 151.1, 150.8, 149.0, 144.0, 138.9, 138.6, 135.8, 135.6, 130.3, 129.6, 127.3, 126.9, 125.9, 125.9, 124.5, 123.9, 122.0, 21.5.

*Product* **3v***: **3-((4-bromophenyl)sulfonyl)-2-phenylbenzo[b]thiophene**.* The sulfonylation product was purified by flash column chromatography on silica gel (PE/AcOEt: 15/1–5/1) to afford **3v** as a white solid (65 mg, 76% yield). ^1^H NMR (600 MHz, CDCl_3_): δ 8.62 (d, J = 6 Hz, 1H), 7.81 (d, J = 6 Hz, 1H), 7.55–7.53 (m, 1H), 7.48–7.43 (m, 6H), 7.42–7.39 (m, 4H). ^13^C NMR (151 MHz, CDCl_3_): δ 153.1, 141.2, 138.1, 136.0, 132.7, 132.0, 131.4, 130.5, 129.7, 128.5, 128.1, 127.7, 126.1, 125.7, 124.5, 121.8.

*Product* **5a***: **2-phenyl-3-tosyl-4H-thiochromen-4-one**.* The sulfonylation product was purified by flash column chromatography on silica gel (PE/AcOEt: 15/1–5/1) to afford **5a** as a white solid (58 mg, 75% yield). ^1^H NMR (600 MHz, CDCl_3_): δ 8.20 (d, J = 7.9 Hz, 1H), 7.84–7.73 (m, 2H), 7.61–7.54 (m, 1H), 7.52–7.34 (m, 7H), 7.19 (d, J = 7.7 Hz, 2H), 2.30 (s, 3H). ^13^C NMR (151 MHz, CDCl_3_): δ 175.9, 162.4, 144.0, 138.9, 135.7, 134.9, 132.8, 132.5, 131.6, 130.1, 128.9, 128.7, 128.5, 128.0, 127.9, 125.3, 21.3.

*Product* **5b: *3-((4-chlorophenyl)sulfonyl)-2-phenyl-4H-thiochromen-4-one***. The sulfonylation product was purified by flash column chromatography on silica gel (PE/AcOEt: 15/1–5/1) to afford **5b** as a yellow solid (66 mg, 80% yield). ^1^H NMR (600 MHz, CDCl_3_): δ 8.28 (dd, J = 8.1, 1.0 Hz, 1H), 7.91–7.86 (m, 2H), 7.58 (td, J = 8.0, 1.4 Hz, 1H), 7.50–7.42 (m, 7H), 7.38–7.34 (m, 2H). ^13^C NMR (151 MHz, CDCl_3_): δ 176.0, 163.2, 140.2, 139.6, 135.7, 134.5, 132.7, 132.6, 131.6, 130.5, 130.3, 129.2, 128.9, 128.7, 128.2, 128.1, 125.3.

*Product* **6: *1,3-dimethyl-3-(tosylmethyl)indolin-2-one***. The sulfonylation product was purified by flash column chromatography on silica gel (PE/AcOEt: 15/1–5/1) to afford **6** as a white solid (50 mg, 76% yield). ^1^H NMR (600 MHz, CDCl_3_): *δ*: 7.38–7.37 (m, 2H), 7.30–7.27 (m, 1H), 7.16 (d, J = 6 Hz, 2H), 7.07 (d, J = 6 Hz, 1H), 6.93–6.90 (m, 1H), 6.84 (d, J = 6 Hz, 1H), 3.85 (d, J = 12 Hz, 1H), 3.67 (d, J = 18 Hz, 1H), 3.16 (s, 3H), 2.39 (s, 3H), 1.38 (s, 3H); ^13^C NMR (151 MHz, CDCl_3_): δ 177.6, 144.3, 143.2, 137.0, 129.6, 129.5, 128.5, 127.8, 124.1, 122.4, 108.3, 61.9, 45.6, 26.5, 25.5, 21.5.

## 4. Conclusions

In summary, we have reported a straightforward strategy for the synthesis of sulfonated benzothiophenes and thioflavones from 2-alkynylthioanisoles and sodium sulfinates via visible-light-induced cascade cyclization reaction under metal- and photocatalyst-free conditions. This approach features good functional group tolerance, simple operation, and mild conditions. The further synthetic application of this strategy is underway in our laboratory.

## Figures and Tables

**Table 1:** Optimization of the reaction conditions *^a^*.

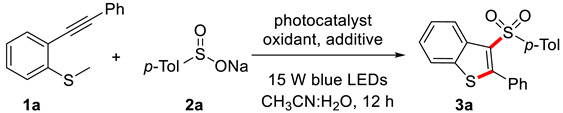				
Entry	Photocatalyst	Oxidant	Additive	Yield (%) *^b^*
1	[Ir(dFCF_3_ppy)_2_dtbbpy]PF_6_	K_2_S_2_O_8_	KI	82
2	[Ir(dFCF_3_ppy)_2_dtbbpy]PF_6_	K_2_S_2_O_8_	NaI	47
3	[Ir(dFCF_3_ppy)_2_dtbbpy]PF_6_	K_2_S_2_O_8_	NaBr	29
4	[Ir(dFCF_3_ppy)_2_dtbbpy]PF_6_	K_2_S_2_O_8_	KCl	35
5	[Ir(dFCF_3_ppy)_2_dtbbpy]PF_6_	Na_2_S_2_O_8_	KI	67
6	[Ir(dFCF_3_ppy)_2_dtbbpy]PF_6_	O_2_	KI	trace
7	[Ir(dFCF_3_ppy)_2_dtbbpy]PF_6_	DTBP	KI	15
8	[Ir(dFCF_3_ppy)_2_dtbbpy]PF_6_	TBHP	KI	23
9	4CzIPN	K_2_S_2_O_8_	KI	41
10	4CzIPN-*^t^*Bu	K_2_S_2_O_8_	KI	47
11	Mes-Acr^+^ClO_4_	K_2_S_2_O_8_	KI	65
12	--	K_2_S_2_O_8_	KI	84
13	--	K_2_S_2_O_8_	--	30
14	--	--	--	24

Reaction conditions: *^a^* **1a** (0.20 mmol), **2a** (0.4 mmol), photocatalyst (1 mol%), additive (30 mol%), oxidant (2 equiv), CH_3_CN/H_2_O (3:1, 2.0 mL), N_2_, 15-W blue LED at room temperature for 12 h; *^b^* isolated yield of the product based on **1**, reaction progress is monitored through TLC.

## Data Availability

Not applicable.

## Supplementary Materials

The following supporting information can be downloaded at: https://www.mdpi.com/article/10.3390/molecules28114436/s1. References [61,62,63,64] are cited in the supplementary materials.
