# Supplementary material for: Photoinduced Photocatalyst-Free Cascade Cyclization of Alkynes with Sodium Sulfinates for the Synthesis of Benzothiophenes and Thioflavones
Source: Molecules. 2023 May 30;28(11):4436. doi: 10.3390/molecules28114436 (PMC10254384; doi:10.3390/molecules28114436)
Supplement: Supplementary file 1 [file molecules-28-04436-s001.zip › molecules-2359273-supplementary.pdf]

Supporting information

Photoinduced Photocatalyst-Free Cascade Cyclization of Alkynes with Sodium Sulfinates for the Synthesis of Benzothiophenes and Thioflavones

Hongqiang Dong [1,*], Chunli Chen [1], Jinlei Zhao [2], Yigang Ji [3,*] and Wenchao Yang [4,5]

[1] The Open Research Fund of the National and Local Joint Engineering Laboratory of High Efficiency and Superior-Quality Cultivation and Fruit Deep Processing Technology of Characteristic Fruit Trees in South Xinjiang, College of Agriculture, Tarim University, Alaer 843300, China; chenchunli1111@163.com
[2] Jiangsu Surveying and Design Institute of Water Resources Co., Ltd., Yangzhou 225127, China; hhuzhjl@163.com
[3] Jiangsu Key Laboratory of Biofuctional Molecules, Department of Life Sciences and Chemistry, Jiangsu Second Normal University, Nanjing 210013, China
[4] National Engineering Research Center of Low-Carbon Processing and Utilization of Forest Biomass, Nanjing Forestry University, Nanjing 210037, China; wccyang@126.com
[5] Guangling College and School of Plant Protection, Yangzhou University, Yangzhou 225009
* Correspondence: dhqzky@163.com (H.D.); ygjnba@126.com (Y.J.)

Table of Contents for Supporting Information

1. General considerations……………………………………….…………….....2

2. Preparation of the starting materials……………………………….……..2

3. General procedure for the synthesis of **3a**………..…………………..………...2

4. Characterization data of products...3

5. ^{1}H NMR, ^{13}C NMR and ^{19}F NMR spectra of the products...............................27

1. General considerations

All reactions were carried out under nitrogen atmosphere. ^{1}H NMR ^{13}C NMR and ^{19}F NMR spectra were measured on a Bruker Avance NMR spectrometer (600 MHz/151 MHz/565 NMR) in $CDCl_3$ as solvent and recorded in ppm relative to internal tetramethylsilane standard. ^{1}H NMR data are reported as follows: δ, chemical shift; coupling constants (*J* are given in Hertz, Hz) and integration. Abbreviations to denote the multiplicity of a particular signal were s (singlet), d (doublet), t (triplet), q (quartet), dd (doublet of doublets) and m (multiplet).

2. Preparation of the starting materials

2-alkynylthioanisoles (**1a**) derivatives were prepared according to reported method [5]. The chemicals and solvents were purchased from commercial supplierseither Aldrich (USA), Energy Chemical (Shanghai) or Shanghai Chemical Company (P. R. China). All solvents were dried and freshly distilled in N_2 prior to use. Products were purified by flash chromatographyon 200-300 mesh silica gel.

3.1 General procedure for the synthesis of 3a and optimization of solvents.

General procedure 1 (GP 1): A dry 15 mL tube was charged with 2-alkynylthioanisole (**1a**, 0.20 mmol), sodium sulfinates (**2a**, 0.40 mmol), $CH_3CN:H_2O$ (3:1, 2 mL), KI (30 mol%), $K_2S_2O_8$ (2 equiv) and a magnetic stir bar. Then let the mixture react under 15W blue led light at room temperature and nitrogen atmosphere for 12 hours. After the reaction, the mixture was concentrated to obtain the crude product, and the crude product was further purified by rapid chromatography (silica gel, petroleum ether / ethyl acetate = 30/1 - 15/1) to obtain the required product **3a**.

3.2 Optimization of solvents.

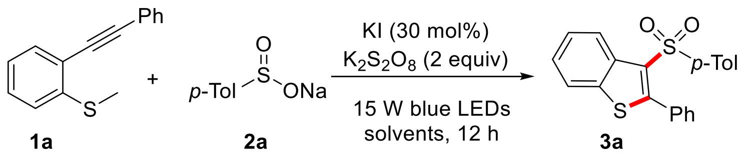

Entry	Oxidant	Additive	Solvents	Yield (%) [b]
1	$K_2S_2O_8$	KI	DMF	26
2	$K_2S_2O_8$	KI	Toluene	38
3	$K_2S_2O_8$	KI	1,4-dioxane	30
4	$K_2S_2O_8$	KI	THF	21
5	$K_2S_2O_8$	KI	MeCN	50
6	$K_2S_2O_8$	KI	$MeCN:H_2O$ (1:1)	58
7	$K_2S_2O_8$	KI	$MeCN:H_2O$ (2:1)	69
8	$K_2S_2O_8$	KI	$MeCN:H_2O$ (5:1)	72

4. Characterization Data for Products

2-phenyl-3-tosylbenzo[*b*]thiophene (3a)[1]

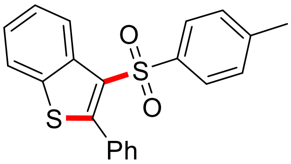

The sulfonylation product was purified by flash column chromatography on silica gel (PE/ AcOEt : 30/1 - 15/1) to afford the **3a** as a white solid (58 mg, 84% yield). **^{1}H NMR (600 MHz, CDCl$_3$)** δ 8.62 (d, J = 8.4 Hz, 1H), 7.76 (d, J = 8.1 Hz, 1H), 7.53 (d, J = 8.3 Hz, 2H), 7.52 – 7.48 (m, 1H), 7.47 – 7.43 (m, 1H), 7.43 – 7.37 (m, 5H), 7.10 (d, J = 8.1 Hz, 2H), 2.30 (s, 3H). **^{13}C NMR (151 MHz, CDCl$_3$)** δ 152.5, 143.9, 139.4, 138.2, 136.1, 131.7, 130.5, 130.3, 129.5, 129.4, 127.6, 127.0, 125.9, 125.6, 124.6, 121.8, 21.5. The characterization data matched the literature [1].

2-phenyl-3-(phenylsulfonyl)benzo[*b*]thiophene (3b) [2]

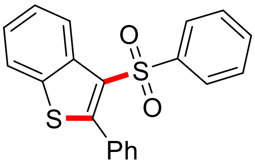

The sulfonylation product was purified by flash column chromatography on silica gel (PE/ AcOEt : 30/1 - 15/1) to afford the **3b** as a white solid (56 mg, 80% yield). **^{1}H NMR (600 MHz, CDCl$_3$)** δ 8.64 (d, J = 8.4 Hz, 1H), 7.80 (d, J = 8.0 Hz, 1H), 7.64 (dd, J = 8.4, 1.1 Hz, 2H), 7.55 – 7.51 (m, 1H), 7.48 – 7.43 (m, 3H), 7.43 – 7.39 (m, 4H), 7.33 (t, J = 7.9 Hz, 2H). **^{13}C NMR (151 MHz, CDCl$_3$)** δ 152.9, 142.3, 138.1, 136.2, 132.9, 131.6, 130.5, 130.0, 129.5, 128.7, 127.7, 126.9, 126.0, 125.6, 124.6, 121.7. The characterization data matched the literature [1].

3-((4-fluorophenyl)sulfonyl)-2-phenylbenzo[*b*]thiophene (3c) [1]

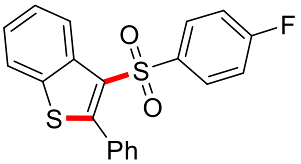

The sulfonylation product was purified by flash column chromatography on silica gel (PE/ AcOEt : 30/1 - 15/1) to afford the **3c** as a yellow solid (46 mg, 63% yield). **^{1}H NMR (600 MHz, CDCl$_3$)** δ 8.65 (d, J = 8.3 Hz, 1H), 7.81 (d, J = 8.0 Hz, 1H), 7.64 – 7.59 (m, 2H), 7.56 – 7.51 (m, 1H), 7.49 – 7.43 (m, 2H), 7.42 – 7.38 (m, 4H), 7.01 – 6.94 (m, 2H). **^{13}C NMR (151 MHz, CDCl$_3$)** δ 166.0, 164.3, 152.8, 138.3 (d, J = 3.1 Hz), 138.1, 136.0, 131.4, 130.5, 130.0, 129.8, 129.7 (d, J = 22.7 Hz), 126.9 (d, J = 252.1 Hz), 125.7, 124.5, 121.8, 115.9 (d, J = 22.8 Hz). The characterization data matched the literature [1].

3-((4-chlorophenyl)sulfonyl)-2-phenylbenzo[*b*]thiophene (3d) [1]

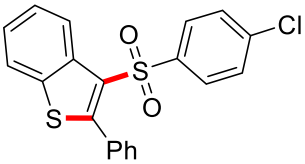

The sulfonylation product was purified by flash column chromatography on silica gel (PE/ AcOEt : 30/1 - 15/1) to afford the **3d** as a yellow solid (57 mg, 75% yield). **^{1}H NMR (600 MHz, CDCl$_3$)** δ 8.63 (d, J = 8.4 Hz, 1H), 7.82 (d, J = 8.1 Hz, 1H), 7.54 (t, J = 8.9 Hz, 3H), 7.50 – 7.44 (m, 2H), 7.43 – 7.38 (m, 4H), 7.28 (d, J = 8.6 Hz, 2H). **^{13}C NMR (151 MHz,**

$CDCl_3$) δ 153.1, 140.7, 139.5, 138.1, 136.0, 131.4, 130.5, 129.7, 129.6, 129.0, 128.4, 127.7, 126.1, 125.7, 124.5, 121.8. The characterization data matched the literature [3].

3-((4-(*tert*-butyl)phenyl)sulfonyl)-2-phenylbenzo[*b*]thiophene (3e) [1]

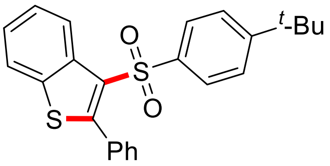

The sulfonylation product was purified by flash column chromatography on silica gel (PE/ AcOEt : 30/1 - 15/1) to afford the **3e** as a white solid (66 mg, 82% yield). **^{1}H NMR (600 MHz, $CDCl_3$)** δ 8.65 (d, J = 8.4 Hz, 1H), 7.81 (d, J = 8.1 Hz, 1H), 7.58 (d, J = 8.6 Hz, 2H), 7.55 – 7.52 (m, 1H), 7.47 – 7.43 (m, 2H), 7.43 – 7.38 (m, 4H), 7.33 (d, J = 8.6 Hz, 2H), 1.26 (s, 9H). **^{13}C NMR (151 MHz, $CDCl_3$)** δ 156.7, 152.4, 139.2, 138.1, 136.2, 131.7, 130.4, 129.4, 127.6, 126.9, 125.9, 125.7, 125.5, 124.7, 121.7, 35.1, 31.0. The characterization data matched the literature [3].

3-((4-methoxyphenyl)sulfonyl)-2-phenylbenzo[*b*]thiophene (3f) [1]

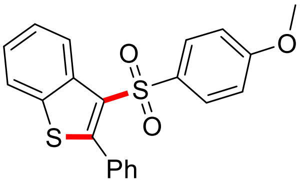

The sulfonylation product was purified by flash column chromatography on silica gel (PE/ AcOEt : 15/1 - 7/1) to afford the **3f** as a white solid (58 mg, 77% yield). **^{1}H NMR (600 MHz, $CDCl_3$)** δ 8.56 (d, J = 8.4 Hz, 1H),

7.73 (d, J = 8.1 Hz, 1H), 7.50 (d, J = 9.0 Hz, 2H), 7.47 – 7.43 (m, 1H), 7.41 – 7.38 (m, 1H), 7.37 – 7.33 (m, 5H), 6.74 – 6.69 (m, 2H), 3.71 (s, 3H). **^{13}C NMR (151 MHz, $CDCl_3$)** δ 162.1, 151.0, 137.1, 135.0, 133.0, 130.7, 129.7, 129.4, 128.3, 128.2, 126.6, 124.8, 124.5, 123.6, 120.7, 112.9, 54.5. The characterization data matched the literature [3].

6-fluoro-2-phenyl-3-tosylbenzo[*b*]thiophene (3g) [1]

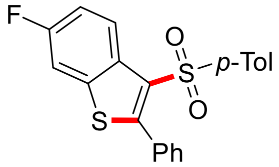

The sulfonylation product was purified by flash column chromatography on silica gel (PE/ AcOEt : 30/1 - 15/1) to afford the **3g** as a brown solid (60 mg, 79% yield). **^{1}H NMR (600 MHz, $CDCl_3$)** δ 8.61 (dd, J = 9.2, 5.1 Hz, 1H), 7.50 (d, J = 8.3 Hz, 2H), 7.48 – 7.44 (m, 2H), 7.41 – 7.38 (m, 4H), 7.29 – 7.24 (m, 1H), 7.12 (d, J = 8.2 Hz, 2H), 2.33 (s, 3H). **^{13}C NMR (151 MHz, $CDCl_3$)** δ 160.8 (d, J = 247.7 Hz), 152.0 (d, J = 3.3 Hz), 144.0, 139.2 (d, J = 4.4 Hz), 139.1, 132.6, 131.3, 130.5, 130.1, 129.6, 129.4, 127.7, 127.0, 126.1 (d, J = 8.9 Hz), 114.9 (d, J = 23.9 Hz), 107.9 (d, J = 25.4 Hz), 21.5. The characterization data matched the literature [3].

6-chloro-2-phenyl-3-tosylbenzo[*b*]thiophene (3h) [2]

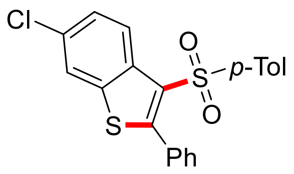

The sulfonylation product was purified by flash column chromatography on silica gel (PE/ AcOEt : 30/1 - 15/1) to afford the **3h** as a white solid (40 mg, 51% yield). **^{1}H NMR (600 MHz, CDCl$_3$)** δ 8.57 (d, J = 8.9 Hz, 1H), 7.78 (d, J = 1.9 Hz, 1H), 7.50 – 7.46 (m, 4H), 7.40 (d, J = 4.4 Hz, 4H), 7.12 (d, J = 8.2 Hz, 2H), 2.33 (s, 3H). **^{13}C NMR (151 MHz, CDCl$_3$)** δ 152.7, 144.1, 139.1, 139.1, 134.6, 131.9, 131.2, 130.5, 130.3, 129.6, 129.4, 127.7, 127.0, 126.8, 125.6, 121.3, 21.5. The characterization data matched the literature [3].

6-bromo-2-phenyl-3-tosylbenzo[*b*]thiophene (3i) [1]

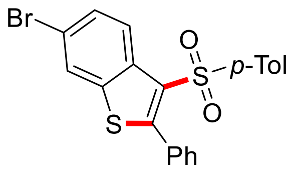

The sulfonylation product was purified by flash column chromatography on silica gel (PE/ AcOEt : 30/1 - 15/1) to afford the **3i** as a yellow solid (60 mg, 68% yield). **^{1}H NMR (600 MHz, CDCl$_3$)** δ 8.51 (d, J = 8.9 Hz, 1H), 7.94 (d, J = 1.8 Hz, 1H), 7.62 (dd, J = 8.9, 1.8 Hz, 1H), 7.48 (t, J = 7.0 Hz, 3H), 7.40 (d, J = 4.4 Hz, 4H), 7.12 (d, J = 8.2 Hz, 2H), 2.33 (s, 3H). **^{13}C NMR (151 MHz, CDCl$_3$)** δ 152.7, 144.1, 139.4, 139.1, 135.0,

131.1, 130.5, 130.3, 129.7, 129.4, 129.4, 127.7, 127.0, 125.8, 124.2, 119.7, 21.5. The characterization data matched the literature [1].

2-(*p*-tolyl)-3-tosylbenzo[*b*]thiophene (3j) [1]

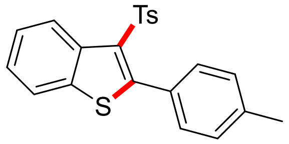

The sulfonylation product was purified by flash column chromatography on silica gel (PE/ AcOEt : 30/1 - 15/1) to afford the **3j** as a white solid (63 mg, 84% yield). **^{1}H NMR (600 MHz, $CDCl_3$)** δ 8.59 (d, J = 8.4 Hz, 1H), 7.76 (d, J = 8.0 Hz, 1H), 7.56 (d, J = 8.3 Hz, 2H), 7.51 – 7.47 (m, 1H), 7.42 – 7.37 (m, 1H), 7.33 (d, J = 8.0 Hz, 2H), 7.21 (d, J = 7.9 Hz, 2H), 7.12 (d, J = 8.2 Hz, 2H), 2.43 (s, 3H), 2.31 (s, 3H). **^{13}C NMR (151 MHz, $CDCl_3$)** δ 153.0, 143.8, 139.6, 139.5, 138.1, 136.2, 130.4, 129.9, 129.4, 128.7, 128.4, 127.0, 125.8, 125.5, 124.5, 121.7, 21.5, 21.5. The characterization data matched the literature [3].

2-(4-ethylphenyl)-3-tosylbenzo[*b*]thiophene (3k)[3]

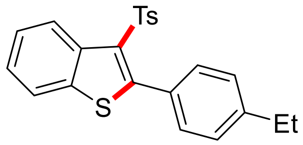

The sulfonylation product was purified by flash column chromatography on silica gel (PE/ AcOEt : 30/1 - 15/1) to afford the **3k** as a white solid (69 mg, 89% yield). **^{1}H NMR (600 MHz, $CDCl_3$)** δ 8.61 (d, J = 8.4 Hz,

1H), 7.74 (d, J = 8.1 Hz, 1H), 7.54 (d, J = 8.3 Hz, 2H), 7.50 – 7.46 (m, 1H), 7.40 – 7.36 (m, 1H), 7.34 (d, J = 8.1 Hz, 2H), 7.22 (d, J = 8.0 Hz, 2H), 7.09 (d, J = 8.2 Hz, 2H), 2.71 (q, J = 7.6 Hz, 2H), 2.29 (s, 3H), 1.29 (t, J = 7.6 Hz, 3H). **^{13}C NMR (151 MHz, CDCl$_3$)** δ 152.9, 145.8, 143.8, 139.5, 138.1, 136.2, 130.5, 130.0, 129.4, 128.9, 127.2, 127.0, 125.8, 125.5, 124.6, 121.7, 28.8, 21.5, 15.4. The characterization data matched the literature. [3]

2-(4-(*tert*-butyl)phenyl)-3-tosylbenzo[*b*]thiophene (3l) [2]

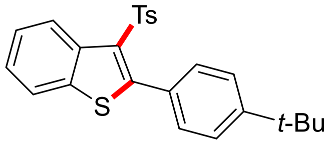

The sulfonylation product was purified by flash column chromatography on silica gel (PE/ AcOEt : 30/1 - 15/1) to afford the **3l** as a white solid (65 mg, 78% yield). **^{1}H NMR (600 MHz, CDCl$_3$)** δ 8.63 (d, J = 8.3 Hz, 1H), 7.78 (d, J = 8.0 Hz, 1H), 7.52 – 7.48 (m, 3H), 7.43 – 7.37 (m, 3H), 7.36 – 7.32 (m, 2H), 7.08 (d, J = 8.1 Hz, 2H), 2.31 (s, 3H), 1.38 (s, 9H). **^{13}C NMR (151 MHz, CDCl$_3$)** δ 152.7, 152.6, 143.6, 139.4, 138.1, 136.3, 130.2, 130.0, 129.2, 128.6, 127.1, 125.8, 125.4, 124.6, 124.6, 121.7, 34.8, 31.3, 21.5. The characterization data matched the literature [1].

2-(4-methoxyphenyl)-3-tosylbenzo[*b*]thiophene (3m) [1]

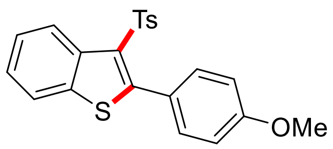

The sulfonylation product was purified by flash column chromatography on silica gel (PE/ AcOEt : 15/1 - 7/1) to afford the **3m** as a white solid (59 mg, 76% yield). **^{1}H NMR (600 MHz, $CDCl_3$)** δ 8.60 (d, J = 8.4 Hz, 1H), 7.77 (d, J = 8.0 Hz, 1H), 7.54 (d, J = 8.3 Hz, 2H), 7.52 – 7.47 (m, 1H), 7.42 – 7.36 (m, 3H), 7.12 (d, J = 8.2 Hz, 2H), 6.93 (d, J = 8.7 Hz, 2H), 3.88 (s, 3H), 2.32 (s, 3H). **^{13}C NMR (151 MHz, $CDCl_3$)** δ 160.6, 152.8, 143.8, 139.5, 138.0, 136.3, 131.9, 129.8, 129.4, 126.9, 125.8, 125.4, 124.5, 123.7, 121.7, 113.1, 55.4, 21.5. The characterization data matched the literature.

2-(4-ethoxyphenyl)-3-tosylbenzo[*b*]thiophene (3n)

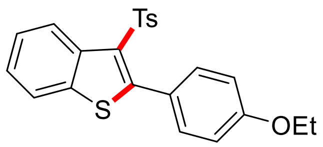

The sulfonylation product was purified by flash column chromatography on silica gel (PE/ AcOEt : 15/1 - 7/1) to afford the **3n** as a white solid (50 mg, 62% yield). **^{1}H NMR (600 MHz, $CDCl_3$)** δ 8.61 (d, J = 8.3 Hz, 1H), 7.77 (d, J = 8.0 Hz, 1H), 7.54 (d, J = 8.3 Hz, 2H), 7.52 – 7.47 (m, 1H), 7.42 – 7.38 (m, 1H), 7.38 – 7.34 (m, 2H), 7.12 (d, J = 8.1 Hz, 2H), 6.94 – 6.89 (m, 2H), 4.10 (q, J = 7.0 Hz, 2H), 2.32 (s, 3H), 1.46 (t, J = 7.0 Hz, 3H). **^{13}C NMR (151 MHz, $CDCl_3$)** δ 160.0, 153.0, 143.7, 139.6, 138.0,

136.3, 131.9, 129.7, 129.3, 126.9, 125.8, 125.4, 124.5, 123.5, 121.6, 113.6, 63.6, 21.5, 14.8. The characterization data matched the literature [2].

2-(*m*-tolyl)-3-tosylbenzo[*b*]thiophene (3o)[3]

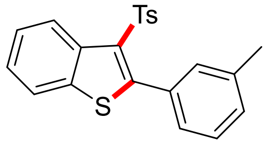

The sulfonylation product was purified by flash column chromatography on silica gel (PE/ AcOEt : 30/1 - 15/1) to afford the **3o** as a white solid (51 mg, 68% yield). **^{1}H NMR (600 MHz, CDCl$_3$)** δ 8.63 (d, J = 8.4 Hz, 1H), 7.79 (d, J = 8.1 Hz, 1H), 7.55 (d, J = 8.3 Hz, 2H), 7.54 – 7.50 (m, 1H), 7.45 – 7.40 (m, 1H), 7.29 (dd, J = 14.9, 7.5 Hz, 2H), 7.23 (d, J = 7.3 Hz, 1H), 7.17 – 7.11 (m, 3H), 2.38 (s, 3H), 2.34 (s, 3H). **^{13}C NMR (151 MHz, CDCl$_3$)** δ 152.7, 143.8, 139.5, 138.1, 137.2, 136.2, 131.5, 131.0, 130.2, 130.1, 129.3, 127.5, 127.1, 125.8, 125.5, 124.6, 121.7, 21.5, 21.3. The characterization data matched the literature [3].

2-(*o*-tolyl)-3-tosylbenzo[*b*]thiophene (3p) [3]

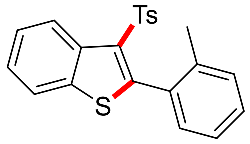

The sulfonylation product was purified by flash column chromatography on silica gel (PE/ AcOEt : 30/1 - 15/1) to afford the **3p** as a white solid

(58 mg, 78% yield). **^{1}H NMR (600 MHz, CDCl$_3$)** δ 8.66 (d, J = 8.3 Hz, 1H), 7.81 (d, J = 8.1 Hz, 1H), 7.54 (t, J = 8.4 Hz, 3H), 7.47 – 7.42 (m, 1H), 7.37 (td, J = 7.6, 1.1 Hz, 1H), 7.22 (dd, J = 14.7, 7.2 Hz, 2H), 7.17 – 7.12 (m, 3H), 2.35 (s, 3H), 2.08 (s, 3H). **^{13}C NMR (151 MHz, CDCl$_3$)** δ 151.6, 144.0, 139.2, 138.5, 138.1, 135.8, 131.2, 131.0, 130.2, 129.8, 129.6, 129.4, 127.3, 125.8, 125.5, 124.9, 124.5, 121.8, 21.5, 20.2. The characterization data matched the literature.

2-(4-fluorophenyl)-3-tosylbenzo[*b*]thiophene (3q)[1]

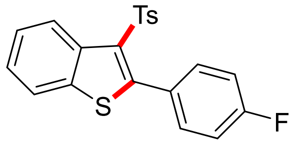

The sulfonylation product was purified by flash column chromatography on silica gel (PE/ AcOEt : 30/1 - 15/1) to afford the **3q** as a yellow solid (54 mg, 71% yield). **^{1}H NMR (600 MHz, CDCl$_3$)** δ 8.61 (d, J = 8.4 Hz, 1H), 7.80 (d, J = 8.1 Hz, 1H), 7.53 (t, J = 8.0 Hz, 3H), 7.43 (m, J = 8.6, 6.7, 1.4 Hz, 3H), 7.15 (d, J = 8.2 Hz, 2H), 7.10 (t, J = 8.6 Hz, 2H), 2.34 (s, 3H). **^{13}C NMR (151 MHz, CDCl$_3$)** δ 162.7, 151.2, 144.0, 139.3, 138.0, 136.0, 132.4 (d, J = 8.4 Hz), 130.6, 129.4, 127.6, 126.9, 126.0, 125.7, 124.6, 121.7, 114.8 (d, J = 21.9 Hz), 21.5. The characterization data matched the literature.

2-(4-chlorophenyl)-3-tosylbenzo[*b*]thiophene (3r) [1]

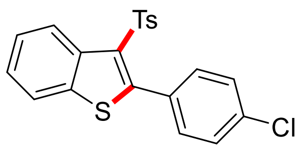

The sulfonylation product was purified by flash column chromatography on silica gel (PE/ AcOEt : 30/1 - 15/1) to afford the **3r** as a yellow solid (55 mg, 70% yield). **^{1}H NMR (600 MHz, CDCl$_3$)** δ 8.60 (d, J = 8.4 Hz, 1H), 7.80 (d, J = 8.0 Hz, 1H), 7.55 (t, J = 5.8 Hz, 2H), 7.54 – 7.51 (m, 1H), 7.46 – 7.42 (m, 1H), 7.40 – 7.36 (m, 4H), 7.16 (d, J = 8.2 Hz, 2H), 2.35 (s, 3H). **^{13}C NMR (151 MHz, CDCl$_3$)** δ 150.8, 144.1, 139.3, 138.1, 136.0, 135.8, 131.8, 130.7, 130.1, 129.5, 127.9, 127.0, 126.0, 125.8, 124.6, 121.7, 21.5. The characterization data matched the literature [3].

2-(naphthalen-1-yl)-3-tosylbenzo[*b*]thiophene (3s)

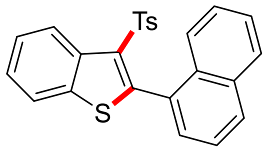

The sulfonylation product was purified by flash column chromatography on silica gel (PE/ AcOEt : 15/1 - 7/1) to afford the **3s** as a yellow solid (43 mg, 53% yield). **^{1}H NMR (600 MHz, CDCl$_3$)** δ 8.60 (d, J = 8.4 Hz, 1H), 7.75 (d, J = 8.0 Hz, 1H), 7.54 (d, J = 8.3 Hz, 2H), 7.51 – 7.46 (m, 1H), 7.38 (m, J = 8.7, 7.4, 1.6 Hz, 3H), 7.11 (d, J = 8.2 Hz, 2H), 6.94 – 6.90 (m, 2H), 3.86 (s, 3H), 2.30 (s, 3H). **^{13}C NMR (151 MHz, CDCl$_3$)** δ 160.6, 152.9, 143.8, 139.6, 138.0, 136.3, 131.9, 131.4, 130.4, 129.8,

129.4, 126.9, 125.8, 125.4, 124.5, 123.7, 121.7, 113.2, 55.4, 53.5, 31.4, 30.2, 21.5. The characterization data matched the literature.[3]

2-(thiophen-2-yl)-3-tosylbenzo[*b*]thiophene (3t) [3]

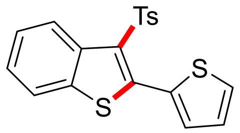

The sulfonylation product was purified by flash column chromatography on silica gel (PE/ AcOEt : 15/1 - 5/1) to afford the **3t** as a yellow solid (56 mg, 77% yield). **^{1}H NMR (600 MHz, $CDCl_3$)** δ 8.67 (d, J = 8.4 Hz, 1H), 7.77 (d, J = 8.1 Hz, 1H), 7.59 (d, J = 8.3 Hz, 2H), 7.53 – 7.49 (m, 3H), 7.44 – 7.40 (m, 1H), 7.17 – 7.11 (m, 3H), 2.33 (s, 3H). **^{13}C NMR (151 MHz, $CDCl_3$)** δ 144.6, 143.9, 139.1, 138.2, 136.5, 132.2, 131.0, 130.7, 129.4, 129.3, 127.4, 126.9, 126.0, 125.8, 124.8, 121.5, 21.5. The characterization data matched the literature.

2-(3-tosylbenzo[*b*]thiophen-2-yl)pyridine (3u)

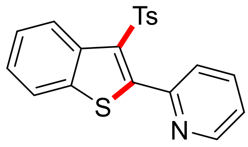

The sulfonylation product was purified by flash column chromatography on silica gel (PE/ AcOEt : 15/1 - 5/1) to afford the **3u** as a white solid (59 mg, 82% yield). **^{1}H NMR (600 MHz, $CDCl_3$)** δ 8.71 – 8.68 (m, 1H), 8.45 (d, J = 8.3 Hz, 1H), 7.85 – 7.79 (m, 5H), 7.51 – 7.46 (m, 1H), 7.43 –

7.40 (m, 1H), 7.40 – 7.37 (m, 1H), 7.20 (d, J = 8.2 Hz, 2H), 2.32 (s, 3H). **^{13}C NMR (151 MHz, CDCl$_3$)** δ 151.1, 150.8, 149.0, 144.0, 138.9, 138.6, 135.8, 135.6, 130.3, 129.6, 127.3, 126.9, 125.9, 125.9, 124.5, 123.9, 122.0, 21.5. The characterization data matched the literature. [2]

3-((4-bromophenyl)sulfonyl)-2-phenylbenzo[*b*]thiophene (3v)

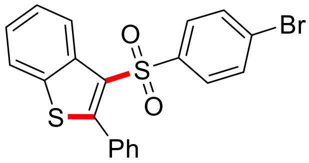

The sulfonylation product was purified by flash column chromatography on silica gel (PE/ AcOEt : 15/1 - 5/1) to afford the **3v** as a white solid (65 mg, 76% yield). **^{1}H NMR (600 MHz, CDCl$_3$)** δ 8.62 (d, J = 6 Hz, 1H), 7.81 (d, J = 6 Hz, 1H), 7.55 – 7.53 (m, 1H), 7.48 – 7.43 (m, 6H), 7.42 – 7.39 (m, 4H). ^{13}C NMR (151 MHz, CDCl$_3$) δ 153.1, 141.2, 138.1, 136.0, 132.7, 132.0, 131.4, 130.5, 129.7, 128.5, 128.1, 127.7, 126.1, 125.7, 124.5, 121.8.

2-phenyl-3-tosyl-4H-thiochromen-4-one (5a)[4]

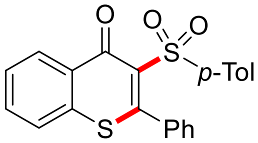

The sulfonylation product was purified by flash column chromatography on silica gel (PE/ AcOEt : 15/1 - 5/1) to afford the **5a** as a white solid (58 mg, 75% yield). **^{1}H NMR (600 MHz, CDCl$_3$)** δ 8.20 (d, J = 7.9 Hz, 1H),

7.84 – 7.73 (m, 2H), 7.61 – 7.54 (m, 1H), 7.52 – 7.34 (m, 7H), 7.19 (d, J = 7.7 Hz, 2H), 2.30 (s, 3H). **13C NMR (151 MHz, $CDCl_3$)** δ 175.9, 162.4, 144.0, 138.9, 135.7, 134.9, 132.8, 132.5, 131.6, 130.1, 128.9, 128.7, 128.5, 128.0, 127.9, 125.3, 21.3. The characterization data matched the literature.[4]

3-((4-chlorophenyl)sulfonyl)-2-phenyl-4H-thiochromen-4-one (5b) [4]

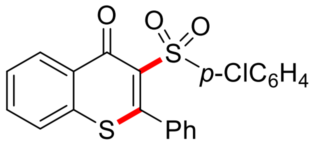

The sulfonylation product was purified by flash column chromatography on silica gel (PE/ AcOEt : 15/1 - 5/1) to afford the **5b** as a yellow solid (66 mg, 80% yield). **1H NMR (600 MHz, $CDCl_3$)** δ 8.28 (dd, J = 8.1, 1.0 Hz, 1H), 7.91 – 7.86 (m, 2H), 7.58 (td, J = 8.0, 1.4 Hz, 1H), 7.50 – 7.42 (m, 7H), 7.38 – 7.34 (m, 2H). **13C NMR (151 MHz, $CDCl_3$)** δ 176.0, 163.2, 140.2, 139.6, 135.7, 134.5, 132.7, 132.6, 131.6, 130.5, 130.3, 129.2, 128.9, 128.7, 128.2, 128.1, 125.3. The characterization data matched the literature.[4]

1,3-dimethyl-3-(tosylmethyl)indolin-2-one (6)

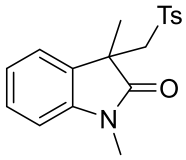

The sulfonylation product was purified by flash column chromatography on silica gel (PE/ AcOEt : 15/1 - 5/1) to afford the **6** as a white solid (50 mg, 76% yield). ^{1}H NMR (600 MHz, $CDCl_3$) δ: 7.38 – 7.37 (m, 2H), 7.30 – 7.27 (m, 1H), 7.16 (d, J = 6 Hz, 2H), 7.07 (d, J = 6 Hz, 1H), 6.93 – 6.90 (m, 1H), 6.84 (d, J = 6 Hz, 1H), 3.85 (d, J = 12 Hz, 1H), 3.67 (d, J = 18 Hz, 1H), 3.16 (s, 3H), 2.39 (s, 3H), 1.38 (s, 3H); ^{13}C NMR (151 MHz, $CDCl_3$) δ 177.6, 144.3, 143.2, 137.0, 129.6, 129.5, 128.5, 127.8, 124.1, 122.4, 108.3, 61.9, 45.6, 26.5, 25.5, 21.5.

References for known compounds:

1. Xu, J.; Yu, X.; Yan, J.; Song, Q. Synthesis of 3-(arylsulfonyl) benzothiophenes and benzoselenophenes via TBHP-initiated radical cyclization of 2-alkynylthioanisoles or-selenoanisoles with sulfinic acids. *Org. Lett.* **2017**, *19*, 6292-6295.
2. Yan, J.; Xu, J.; Zhou, Y.; Chen, J.; Song, Q. A facile synthesis of diverse 5-arylated triazoles *via* a Cu-catalyzed oxidative interrupted click reaction with arylboronic acids in air**.** *Org. Chem. Front.* **2018**, *5*, 1483-1487.
3. Zhang, D.; Cai, J.; Du, J.; Wang, X.; He, W.; Yang, Z.; Liu, C.; Fang, Z.; Guo, K. Oxidant-and Catalyst-Free Synthesis of Sulfonated Benzothiophenes via Electrooxidative Tandem Cyclization. *J. Org. Chem.* **2021**, *86*, 2593-2601.
4. Jiang, Y.; Li, J.; Feng, Z.; Xu, G.; Shi, X.; Ding, Q.; Li, W.; Ma, C.; Yu, B. Ethylene Glycol: A Green Solvent for Visible Light-Promoted Aerobic Transition Metal-Free Cascade Sulfonation/Cyclization Reaction. *Adv. Synth. Catal.* **2020**, *362*, 2609-2614.

5 Liu, W.; Hu, Y.-Q.; Hong, X.-Y.; Li, G.-X.; Huang, X.-B.; Gao, W.-X.; Liu, M.-C.; Xia, Y.; Zhou, Y.-B.; Wu, H.-Y. Direct synthesis of 3-acylbenzothiophenes via the radical cyclization of 2-alkynylthioanisoles with α-oxocarboxylic acids. *Chem. Commun.* **2018**, *54*, 14148-14151

5. ^{1}H NMR, ^{13}C NMR and ^{19}F NMR spectra of the products

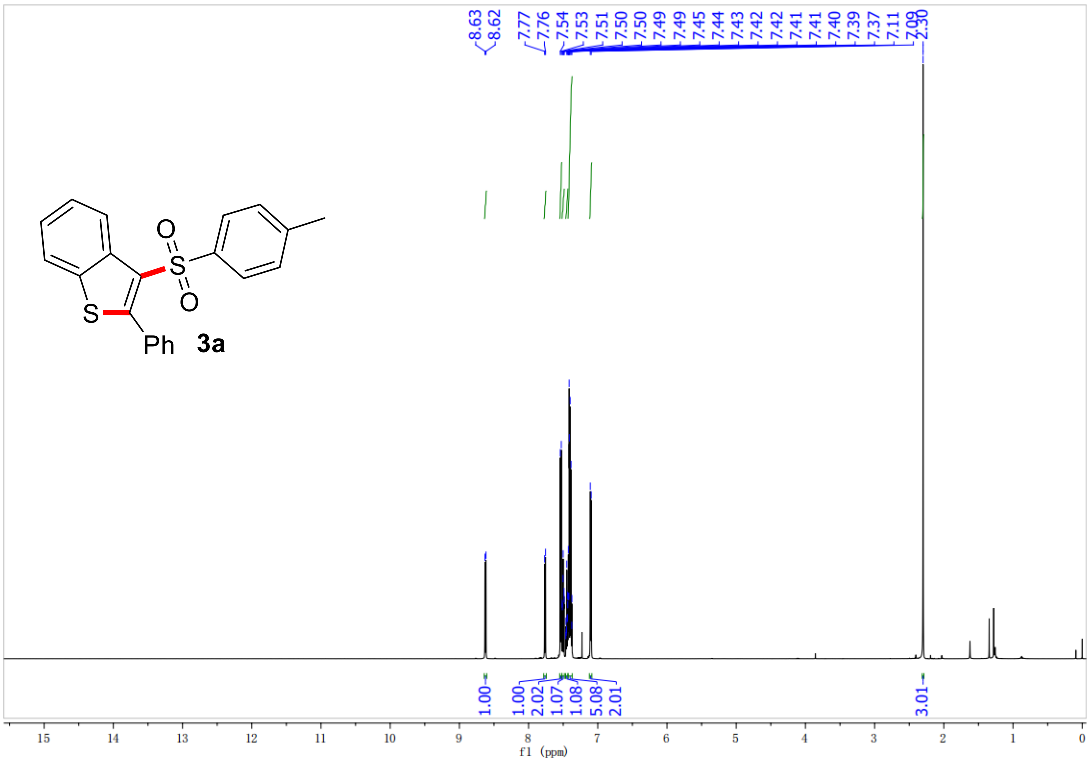

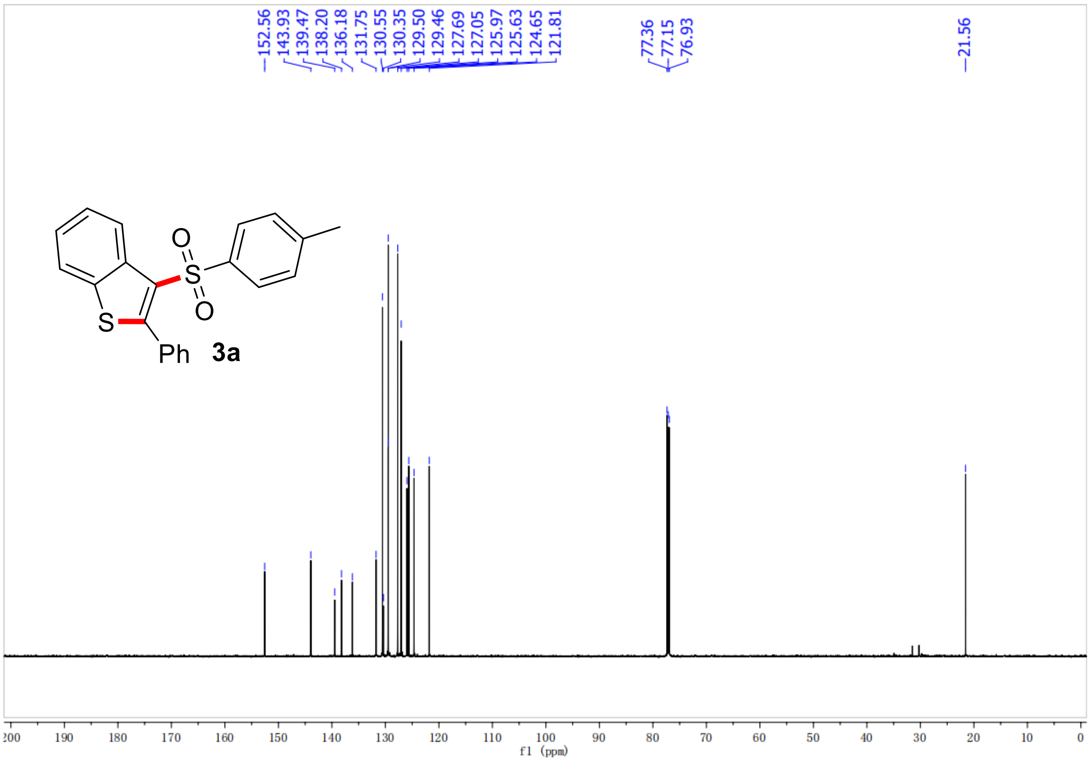

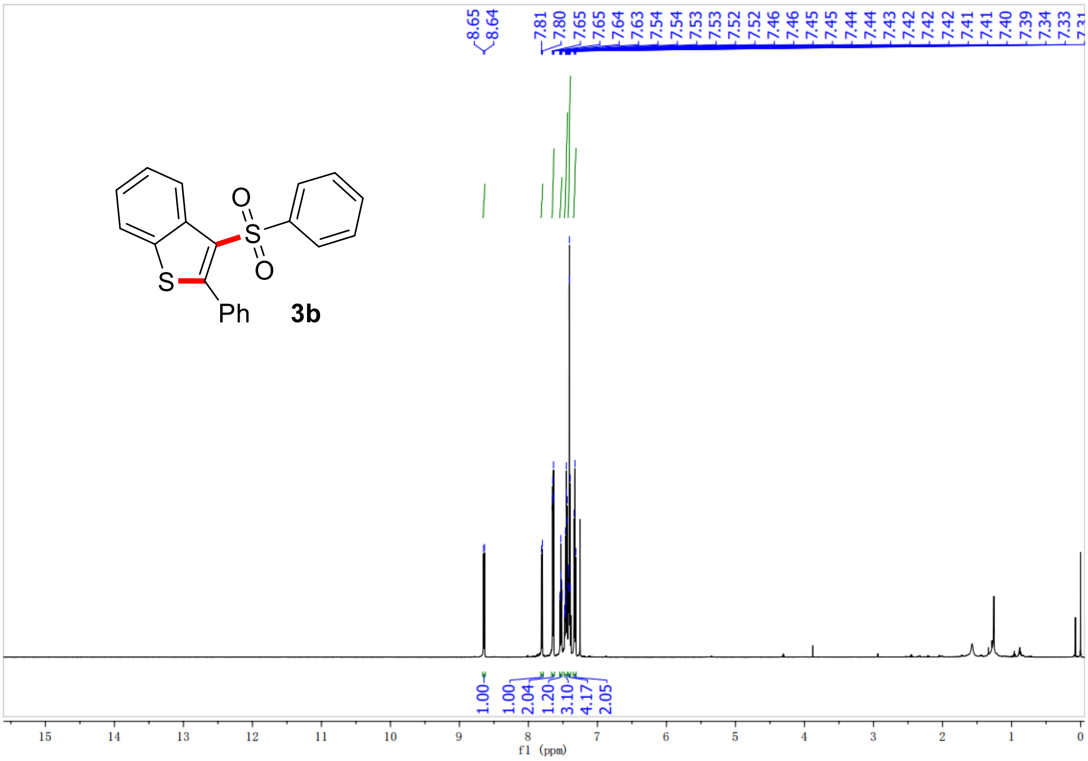

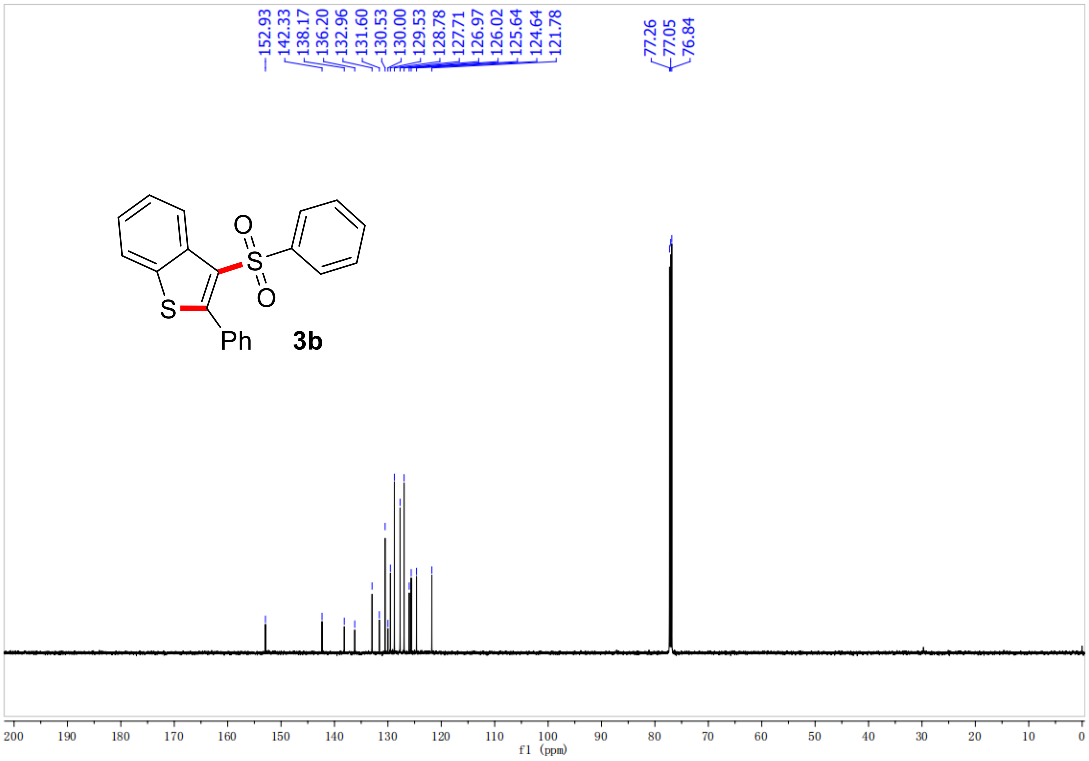

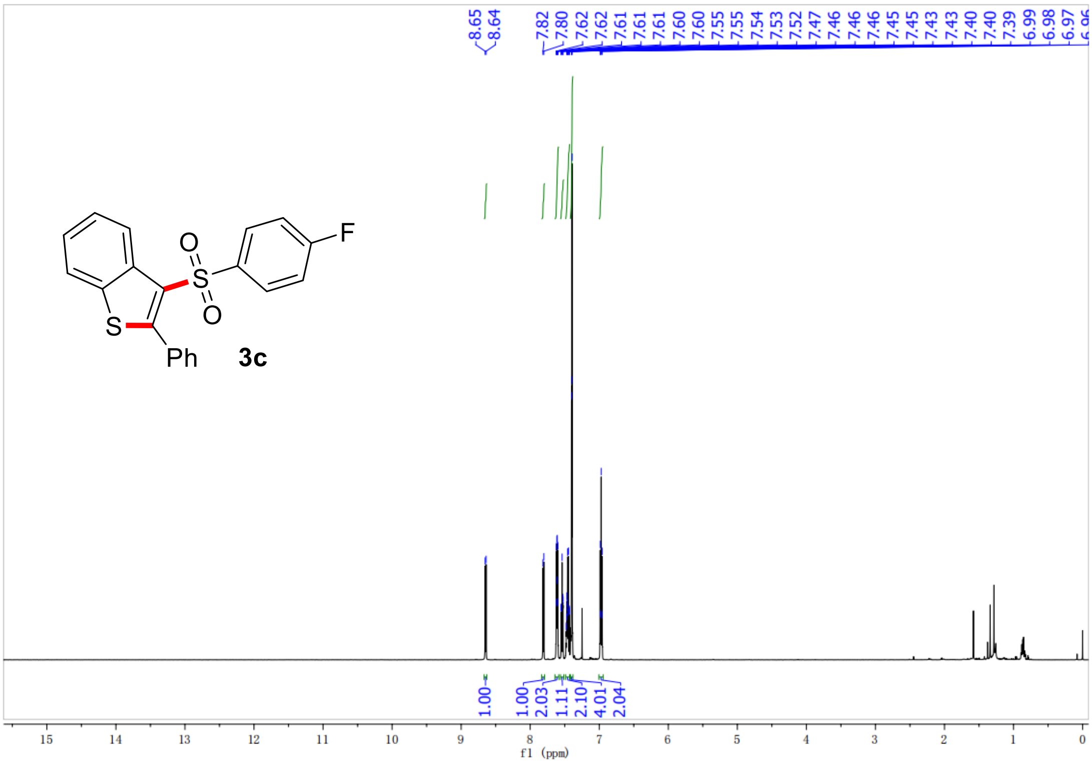
3c
Ph
F
f1 (ppm)

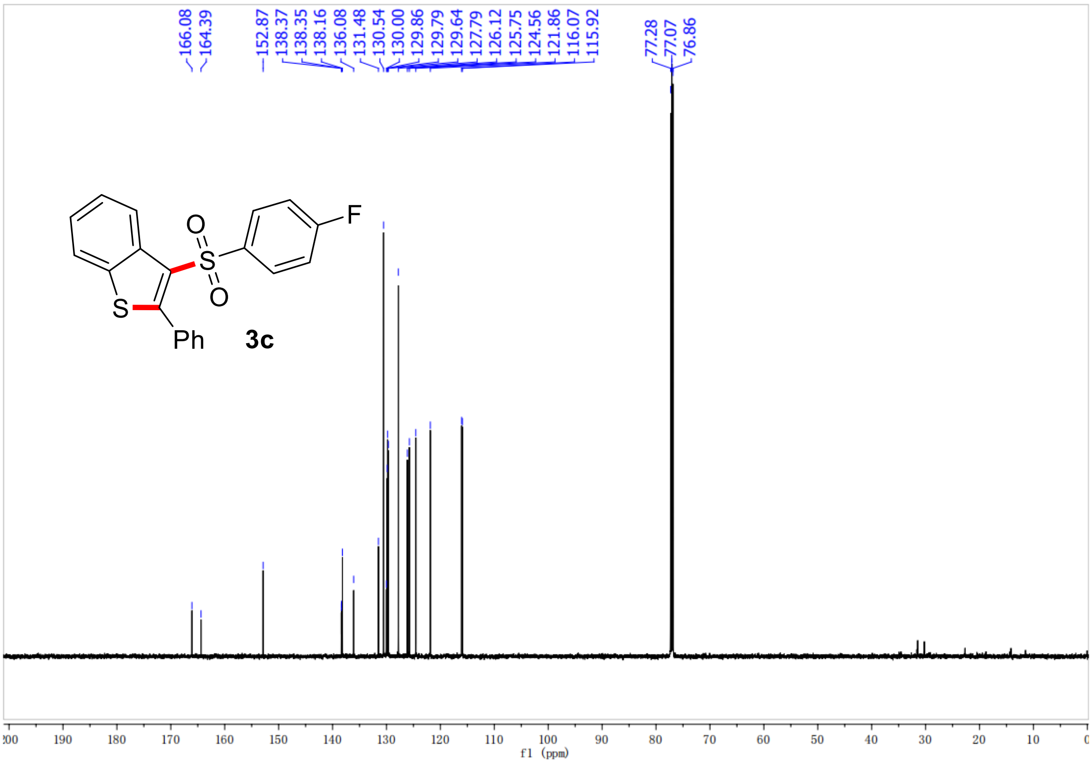
3c
Ph
F
f1 (ppm)

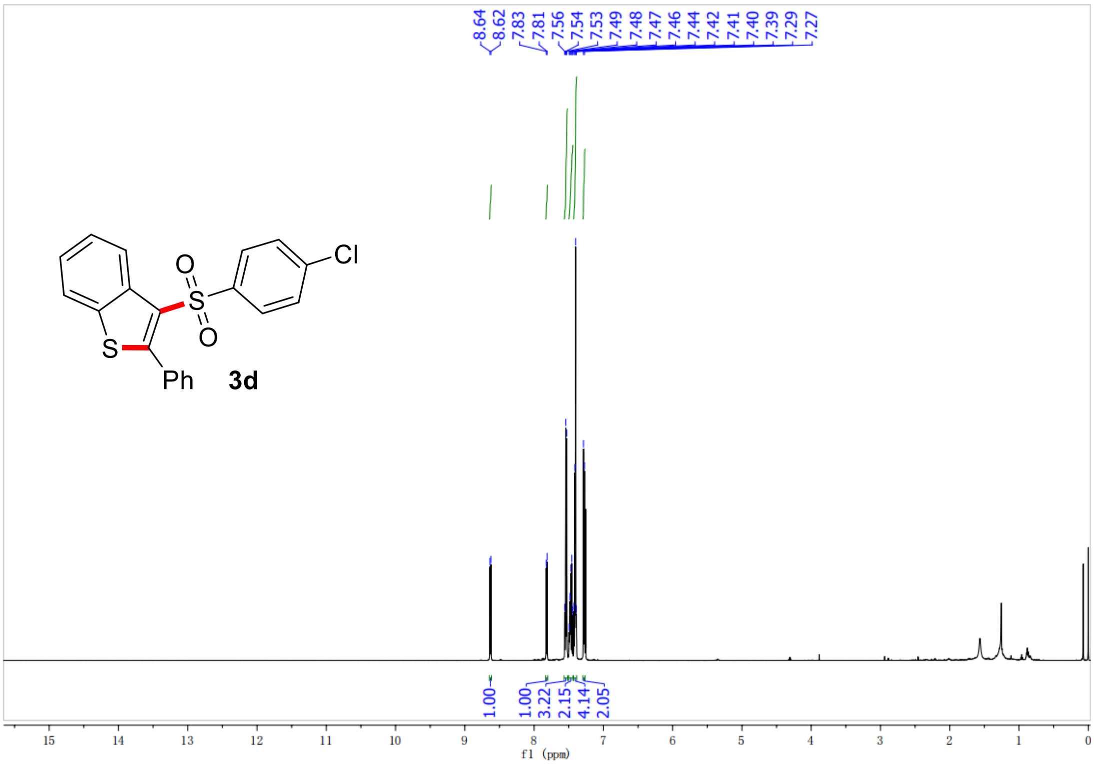
8.64
8.62
7.83
7.81
7.56
7.54
7.53
7.49
7.48
7.47
7.46
7.44
7.42
7.41
7.40
7.39
7.29
7.27
Cl
O
S
O
S
Ph
3d
1.00
1.00
3.22
2.15
4.14
2.05
15 14 13 12 11 10 9 8 7 6 5 4 3 2 1 0
f1 (ppm)

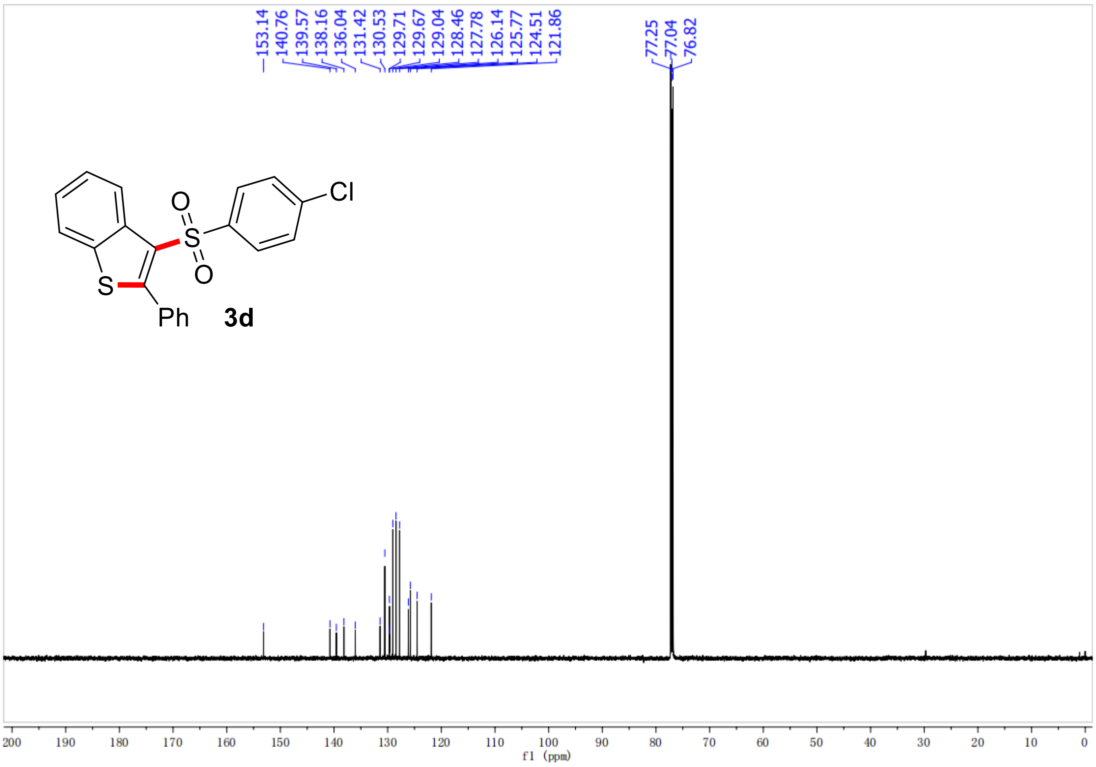
153.14
140.76
139.57
138.16
136.04
131.42
130.53
129.71
129.67
129.04
128.46
127.78
126.14
125.77
124.51
121.86
77.25
77.04
76.82
Cl
O
S
O
S
Ph
3d
200 190 180 170 160 150 140 130 120 110 100 90 80 70 60 50 40 30 20 10 0
f1 (ppm)

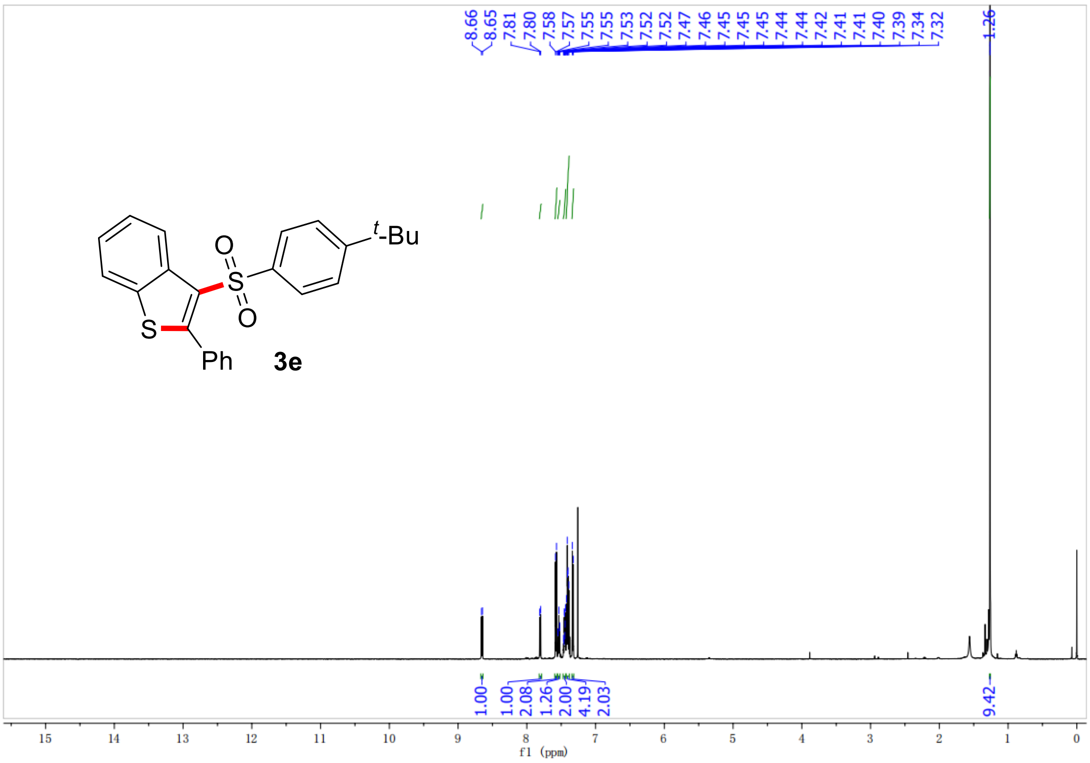
8.66
8.65
7.81
7.80
7.58
7.57
7.55
7.55
7.53
7.52
7.52
7.47
7.46
7.45
7.45
7.45
7.44
7.44
7.42
7.41
7.41
7.40
7.39
7.34
7.32
1.26
t-Bu
O
S
O
S
Ph
3e
1.00
1.00
2.08
1.26
2.00
4.19
2.03
9.42
15
14
13
12
11
10
9
8
7
6
5
4
3
2
1
0
f1 (ppm)

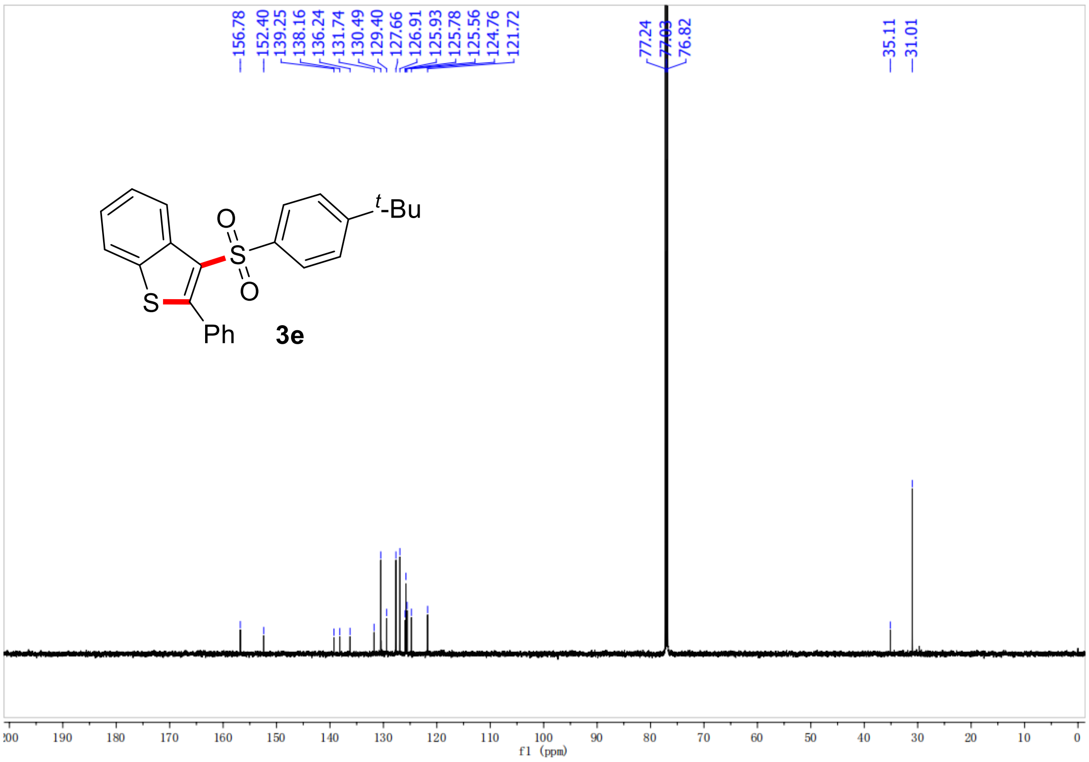
156.78
152.40
139.25
138.16
136.24
131.74
130.49
129.40
127.66
126.91
125.93
125.78
125.56
124.76
121.72
77.24
77.03
76.82
35.11
31.01
t-Bu
O
S
O
S
Ph
3e
190
180
170
160
150
140
130
120
110
100
90
80
70
60
50
40
30
20
10
0
f1 (ppm)

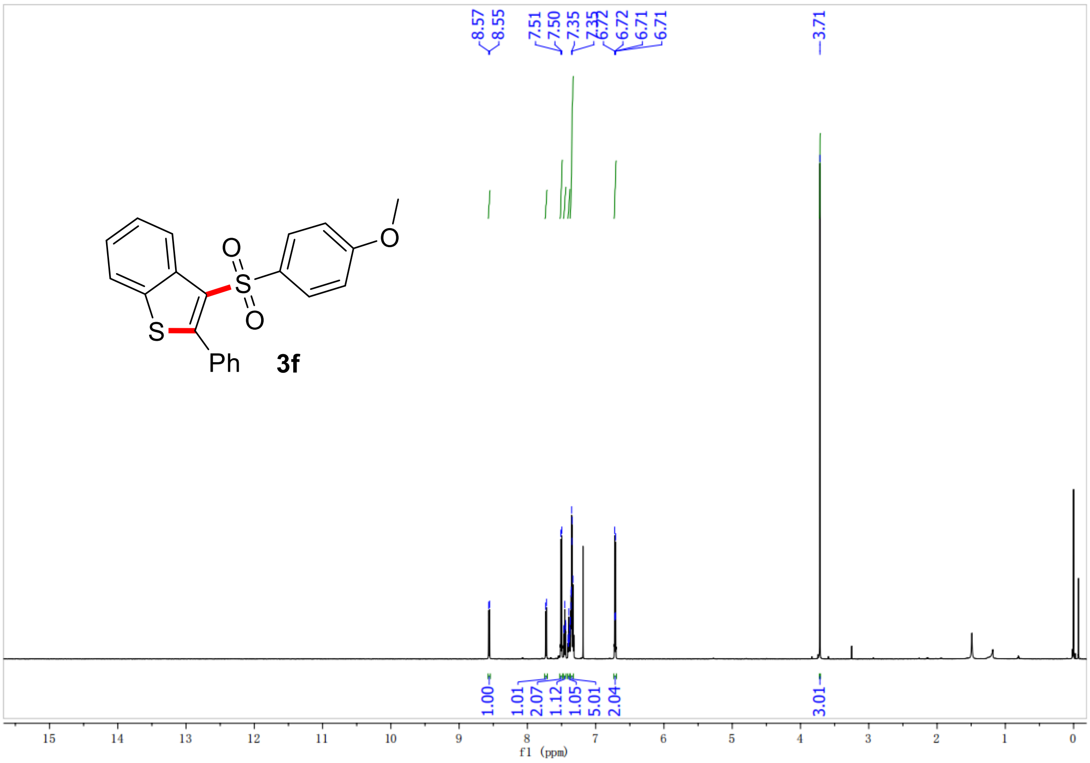
8.57
8.55
7.51
7.50
7.35
7.35
6.72
6.72
6.71
6.71
3.71
S
O
O
O
Ph
3f
1.00
1.01
2.07
1.12
1.05
5.01
2.04
3.01
f1 (ppm)

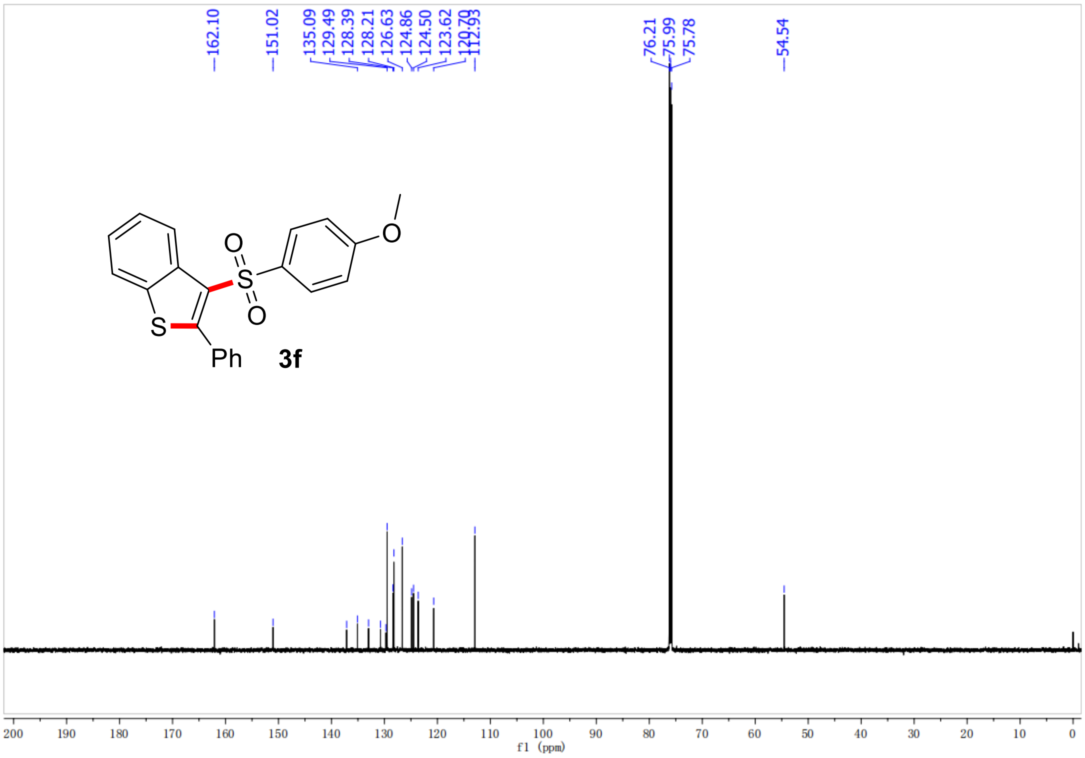
162.10
151.02
135.09
129.49
128.39
128.21
126.63
124.86
124.50
123.62
112.93
76.21
75.99
75.78
54.54
S
O
O
O
Ph
3f
f1 (ppm)

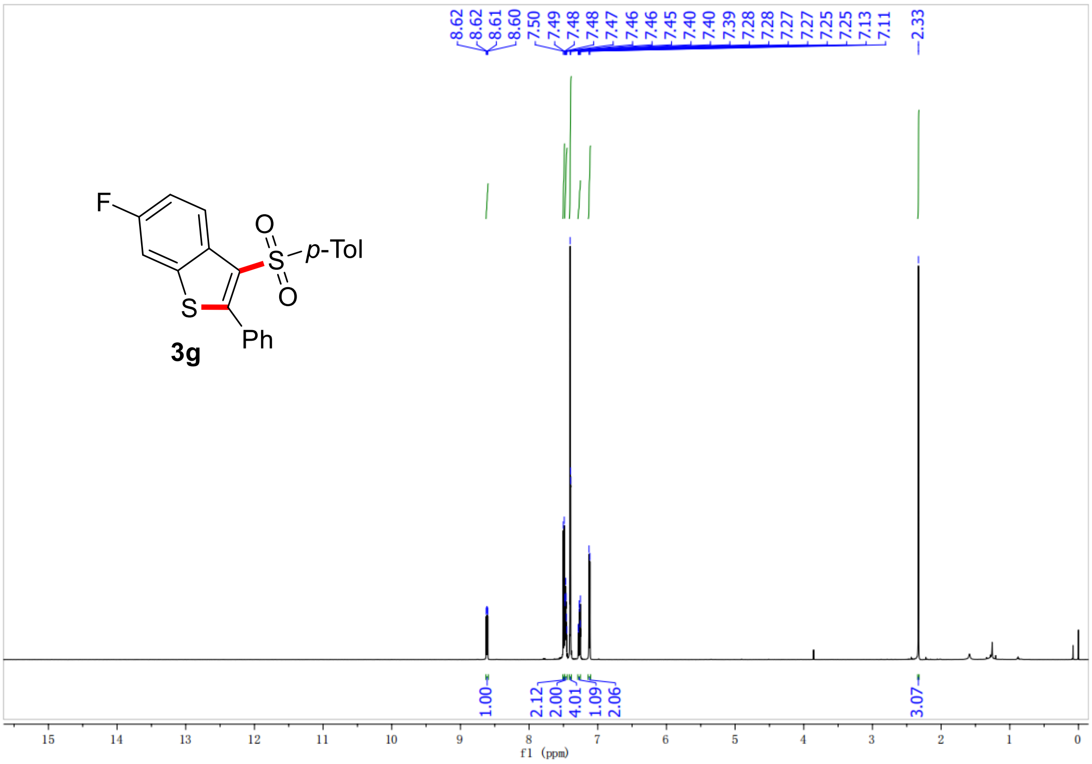
8.62
8.62
8.61
8.60
7.50
7.49
7.48
7.48
7.47
7.46
7.45
7.40
7.40
7.39
7.28
7.28
7.27
7.27
7.25
7.25
7.13
7.11
2.33
F
O
S
p-Tol
O
S
Ph
3g
1.00
2.12
2.00
4.01
1.09
2.06
3.07
f1 (ppm)

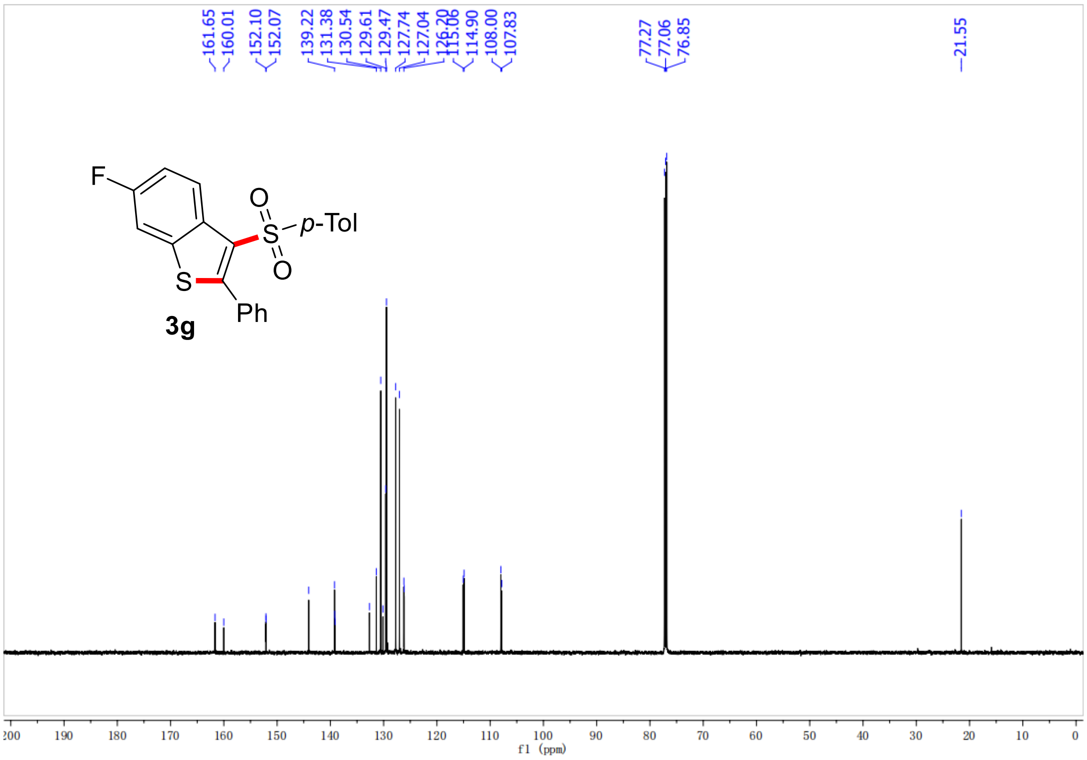
161.65
160.01
152.10
152.07
139.22
131.38
130.54
129.61
129.47
127.74
127.04
126.20
115.06
114.90
108.00
107.83
77.27
77.06
76.85
21.55
F
O
S
p-Tol
O
S
Ph
3g
f1 (ppm)

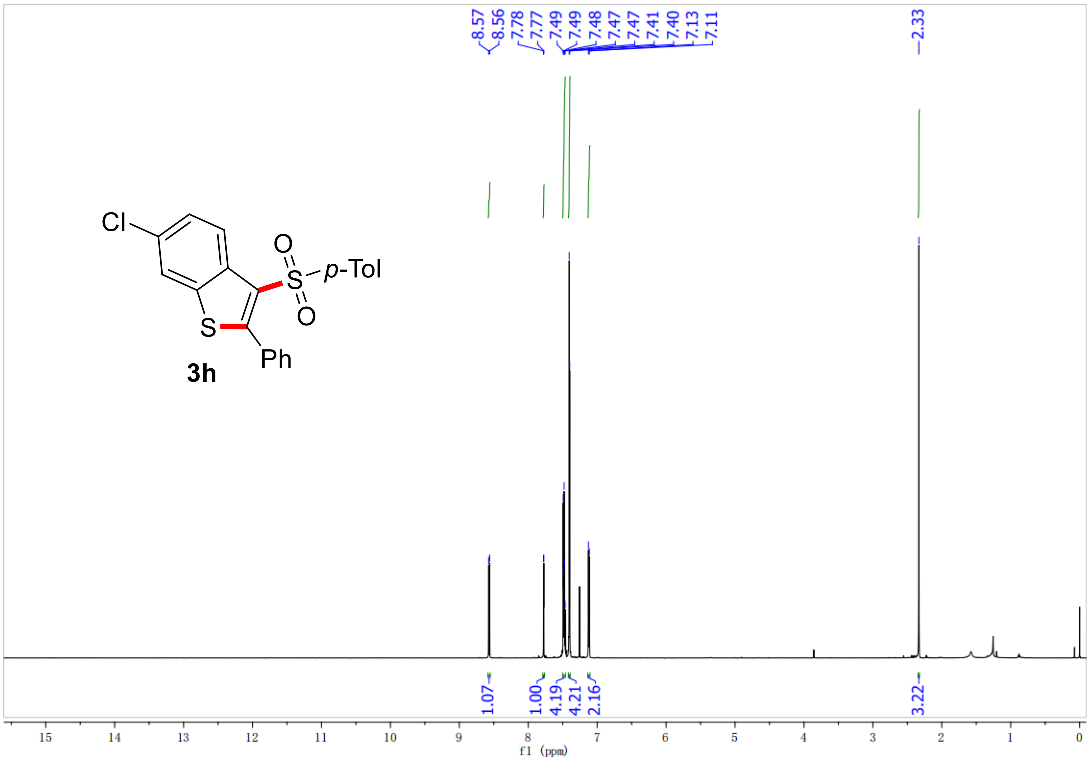
3h
8.57
8.56
7.78
7.77
7.49
7.49
7.48
7.47
7.47
7.41
7.40
7.13
7.11
2.33
1.07
1.00
4.19
4.21
2.16
3.22
f1 (ppm)

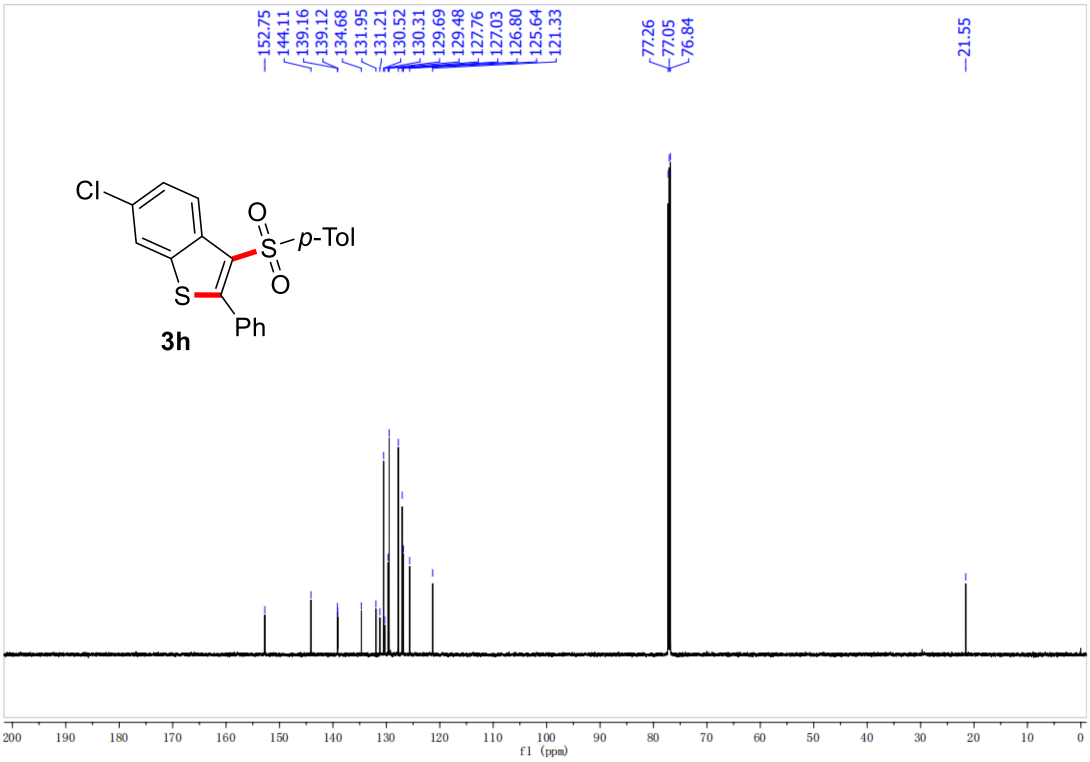
3h
152.75
144.11
139.16
139.12
134.68
131.95
131.21
130.52
130.31
129.69
129.48
127.76
127.03
126.80
125.64
121.33
77.26
77.05
76.84
21.55
f1 (ppm)

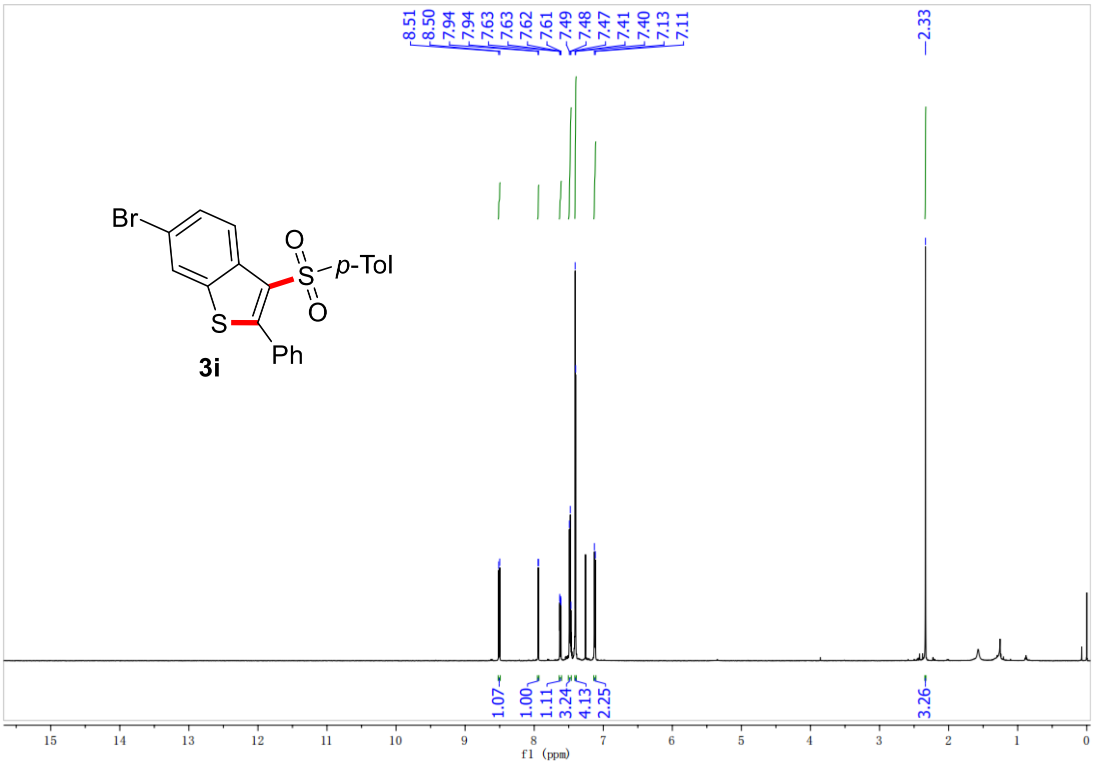
8.51
8.50
7.94
7.94
7.63
7.63
7.62
7.61
7.49
7.48
7.47
7.41
7.40
7.13
7.11
2.33
Br
O
S
p-Tol
O
S
Ph
3i
1.07
1.00
1.11
3.24
4.13
2.25
3.26
f1 (ppm)

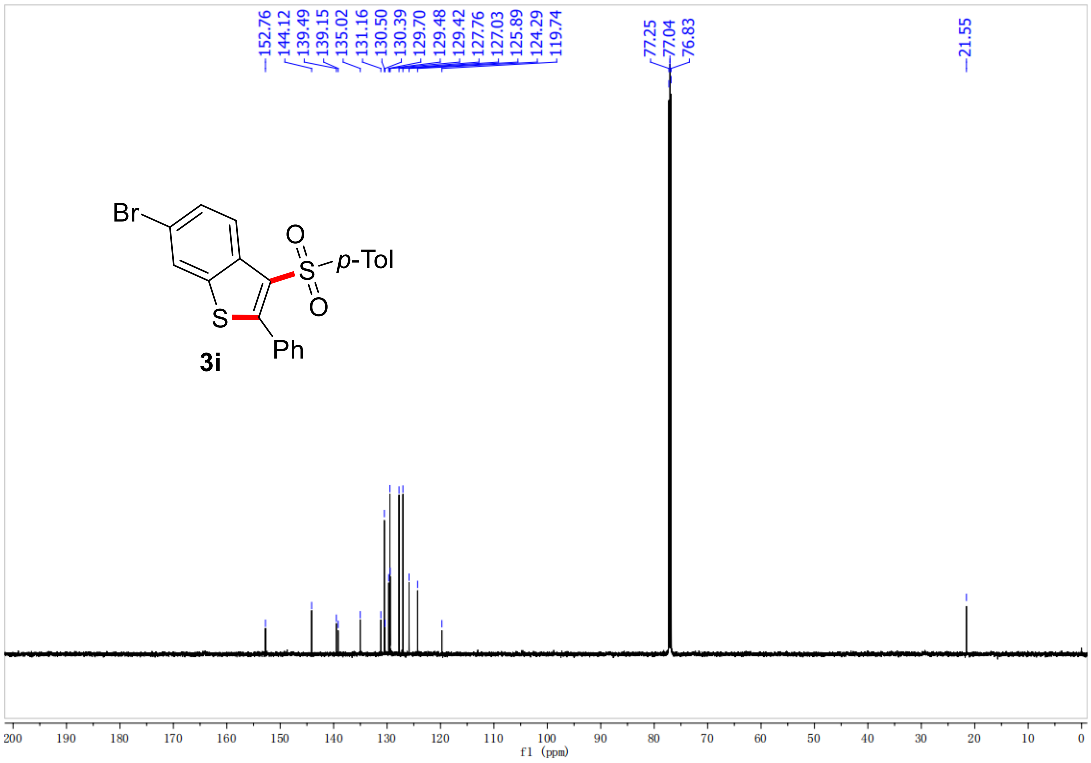
152.76
144.12
139.49
139.15
135.02
131.16
130.50
130.39
129.70
129.48
129.42
127.76
127.03
125.89
124.29
119.74
77.25
77.04
76.83
21.55
Br
O
S
p-Tol
O
S
Ph
3i
f1 (ppm)

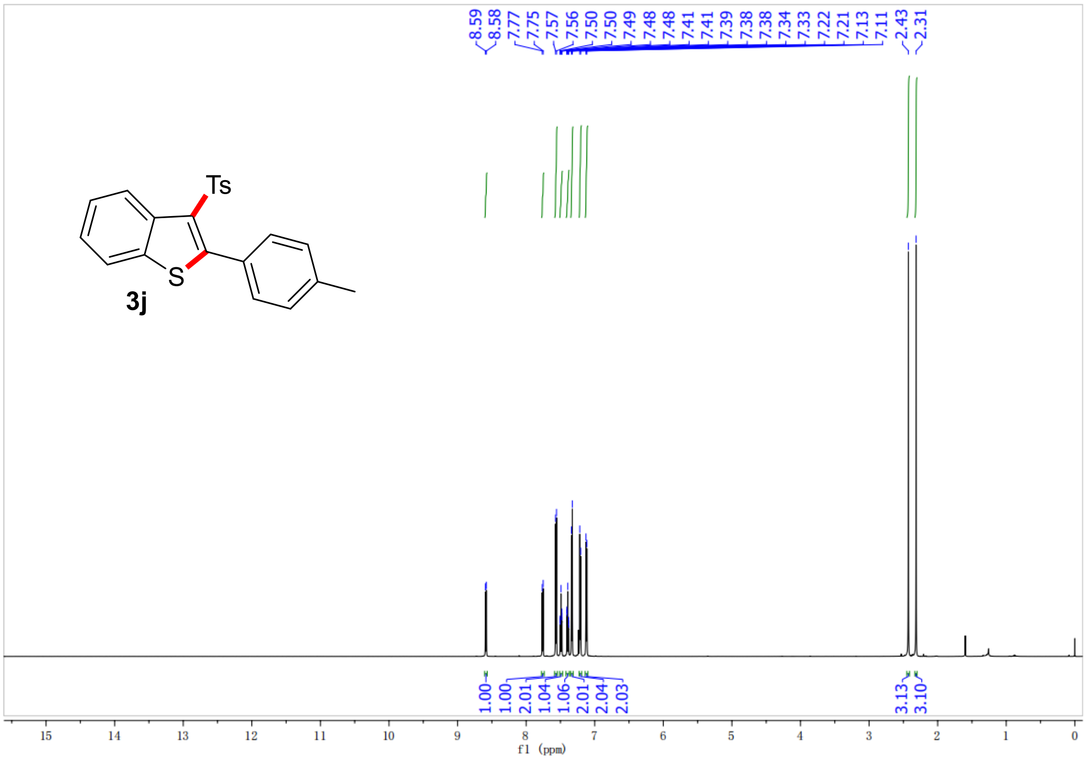
8.59
8.58
7.77
7.75
7.57
7.56
7.50
7.50
7.49
7.48
7.48
7.41
7.41
7.39
7.38
7.38
7.34
7.33
7.22
7.21
7.13
7.11
2.43
2.31
Ts
S
3j
1.00
1.00
2.01
1.04
1.06
2.01
2.04
2.03
3.13
3.10
f1 (ppm)

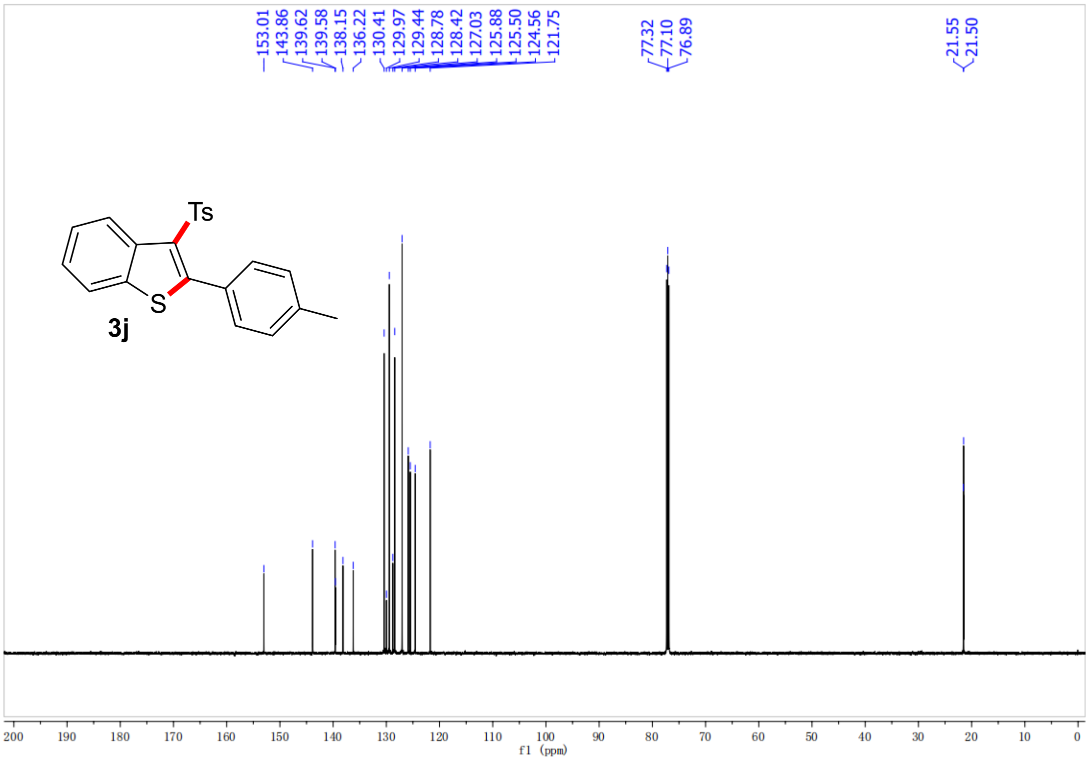
153.01
143.86
139.62
139.58
138.15
136.22
130.41
129.97
129.44
128.78
128.42
127.03
125.88
125.50
124.56
121.75
77.32
77.10
76.89
21.55
21.50
Ts
S
3j
f1 (ppm)

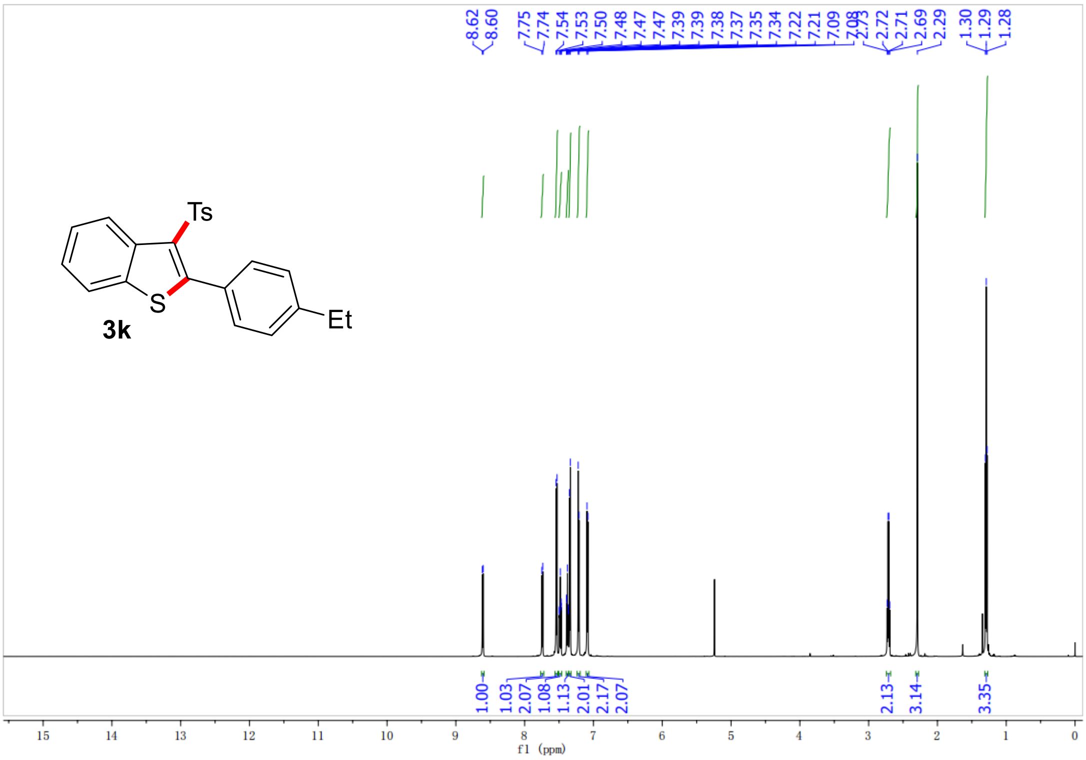
Ts
S
Et
3k
8.62
8.60
7.75
7.74
7.54
7.53
7.50
7.48
7.47
7.47
7.39
7.39
7.38
7.37
7.35
7.34
7.22
7.21
7.09
7.08
2.73
2.72
2.71
2.69
2.29
1.30
1.29
1.28
1.00
1.03
2.07
1.08
1.13
2.01
2.17
2.07
2.13
3.14
3.35
15 14 13 12 11 10 9 8 7 6 5 4 3 2 1 0
f1 (ppm)

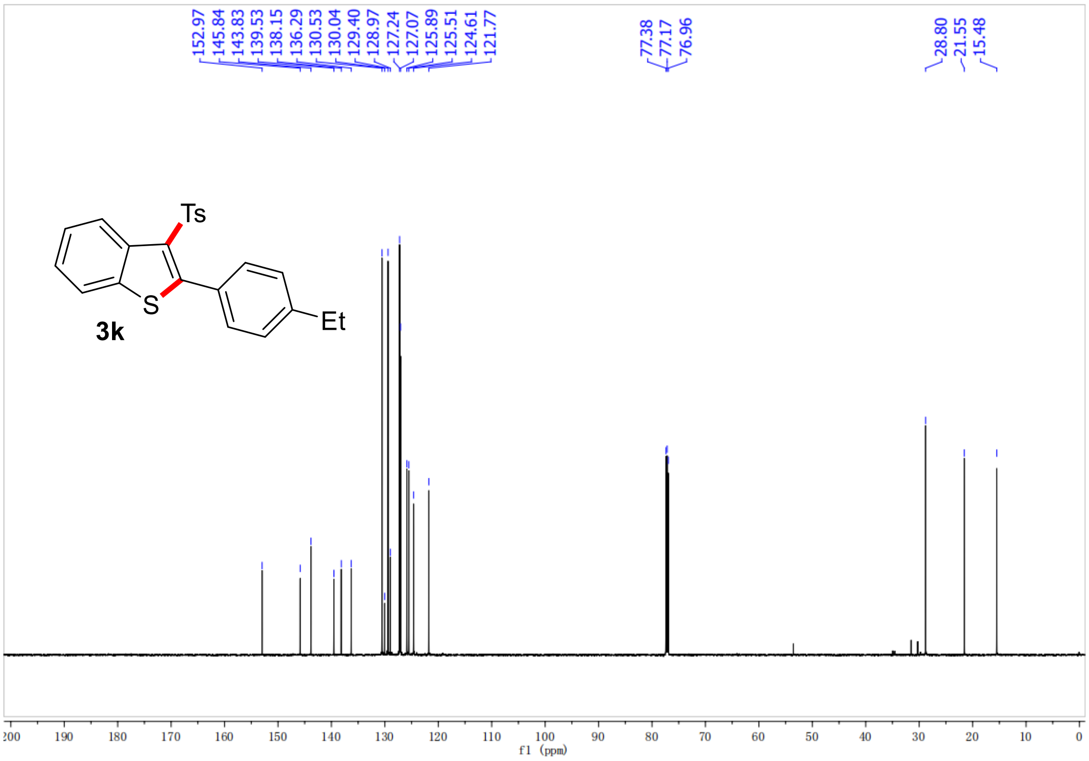
Ts
S
Et
3k
152.97
145.84
143.83
139.53
138.15
136.29
130.53
130.04
129.40
128.97
127.24
127.07
125.89
125.51
124.61
121.77
77.38
77.17
76.96
28.80
21.55
15.48
200 190 180 170 160 150 140 130 120 110 100 90 80 70 60 50 40 30 20 10 0
f1 (ppm)

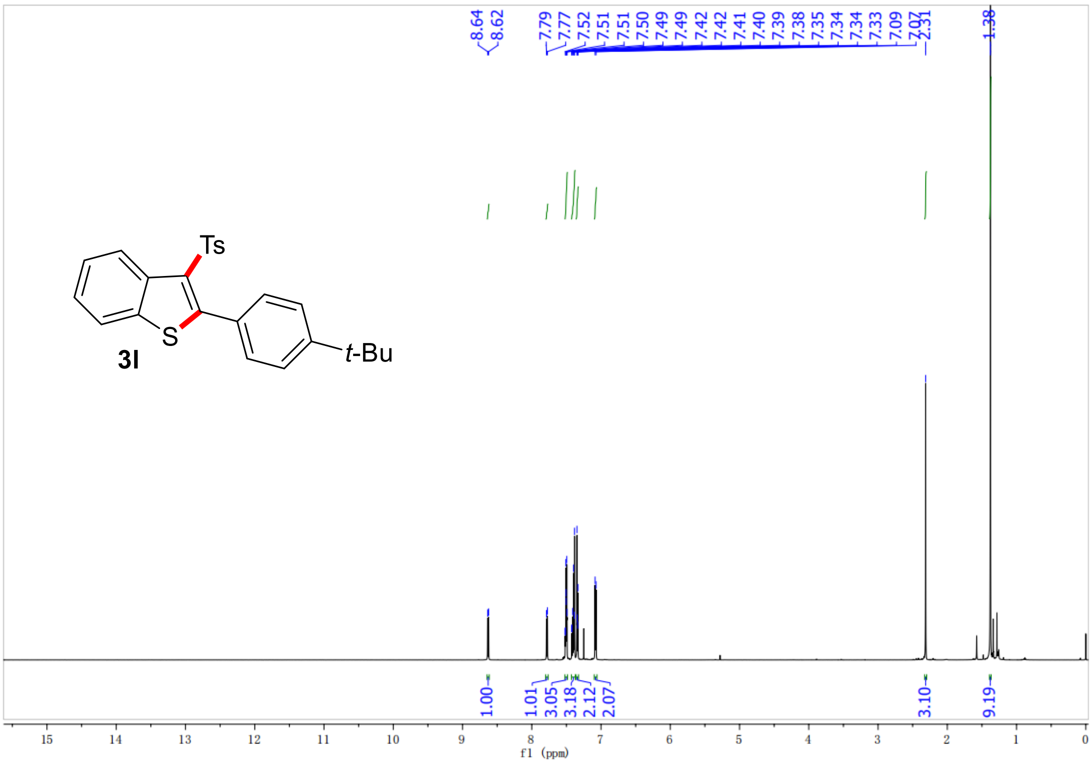
8.64
8.62
7.79
7.77
7.52
7.51
7.51
7.50
7.49
7.49
7.42
7.42
7.41
7.40
7.39
7.38
7.35
7.34
7.34
7.33
7.09
7.07
2.31
1.38
Ts
S
3l
t-Bu
1.00
1.01
3.05
3.18
2.12
2.07
3.10
9.19
f1 (ppm)

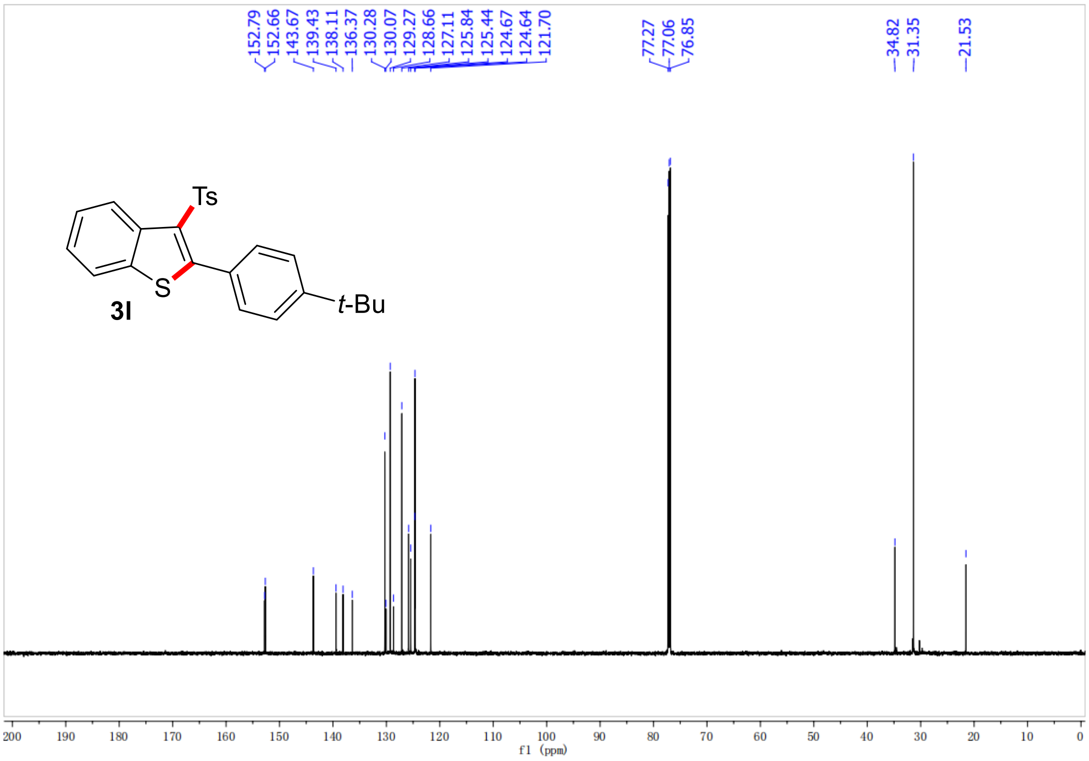
152.79
152.66
143.67
139.43
138.11
136.37
130.28
130.07
129.27
128.66
127.11
125.84
125.44
124.67
124.64
121.70
77.27
77.06
76.85
34.82
31.35
21.53
Ts
S
3l
t-Bu
f1 (ppm)

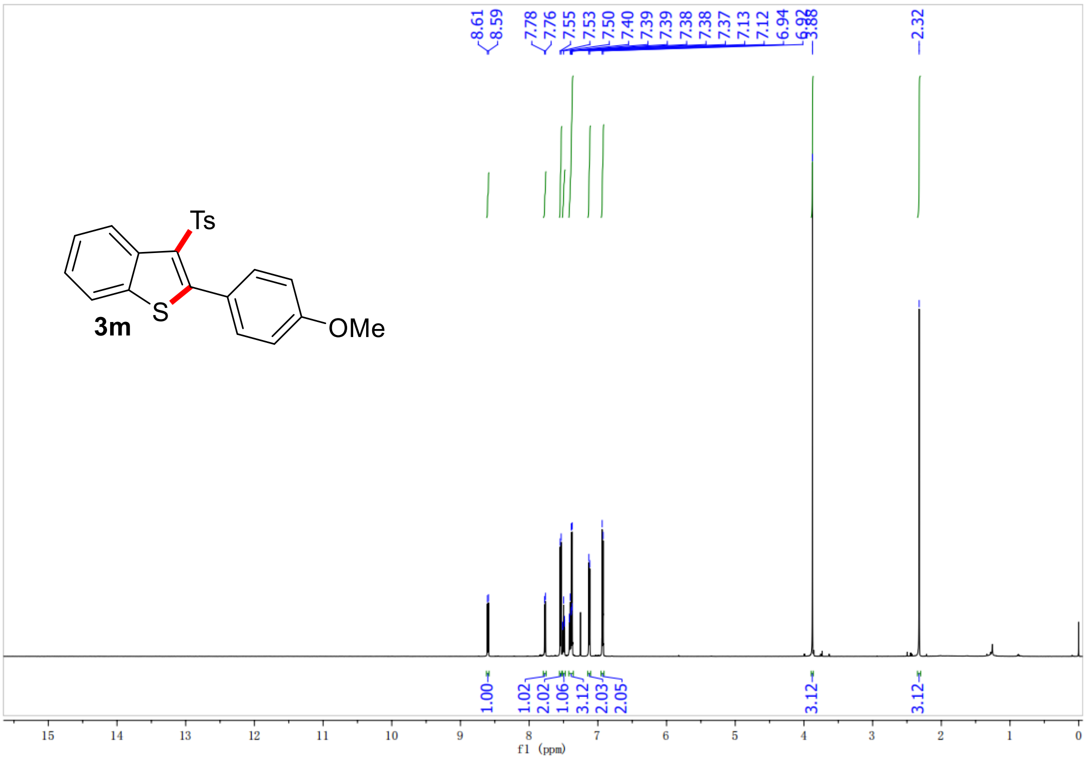
8.61
8.59
7.78
7.76
7.55
7.53
7.50
7.40
7.39
7.39
7.38
7.38
7.37
7.13
7.12
6.94
6.92
3.88
2.32
Ts
S
3m
OMe
1.00
1.02
2.02
1.06
3.12
2.03
2.05
3.12
3.12
f1 (ppm)

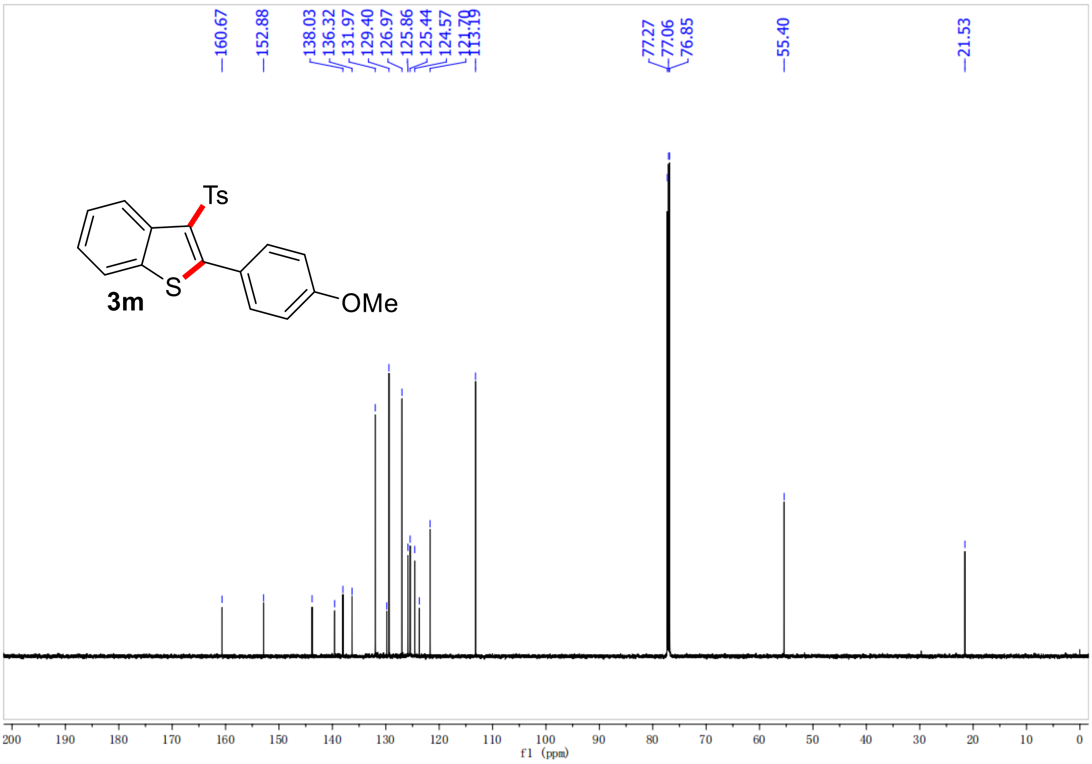
160.67
152.88
138.03
136.32
131.97
129.40
126.97
125.86
125.44
124.57
121.70
113.19
77.27
77.06
76.85
55.40
21.53
Ts
S
3m
OMe
f1 (ppm)

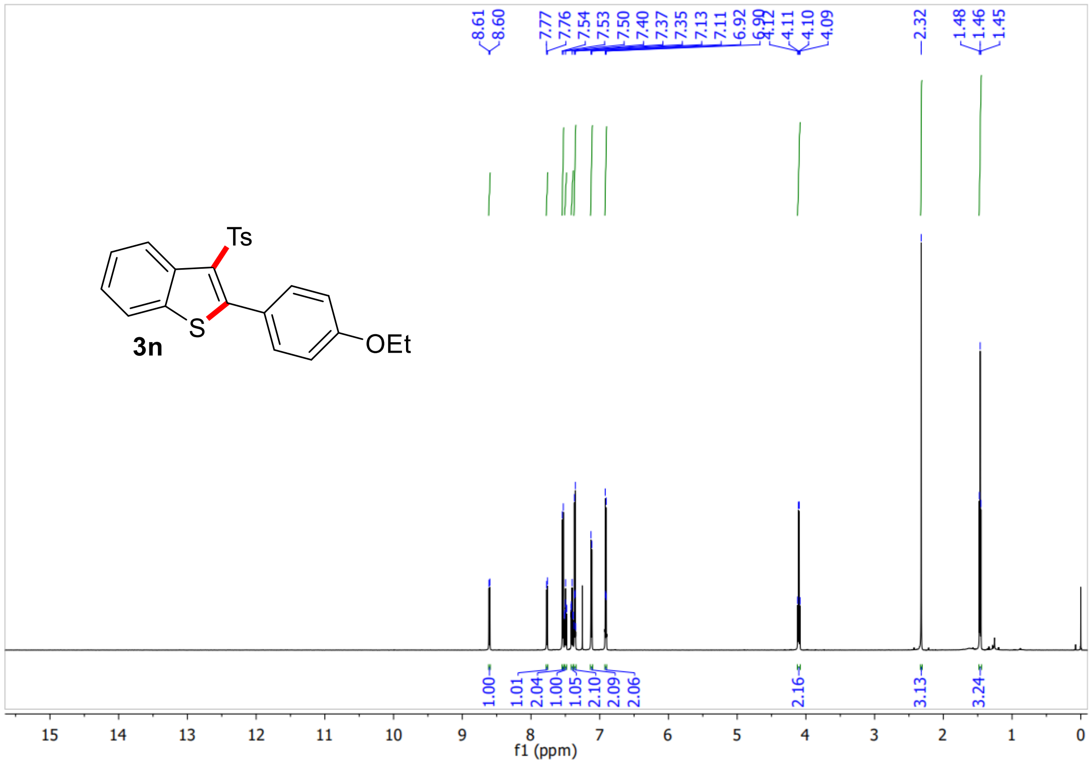
3n
Ts
S
OEt
f1 (ppm)

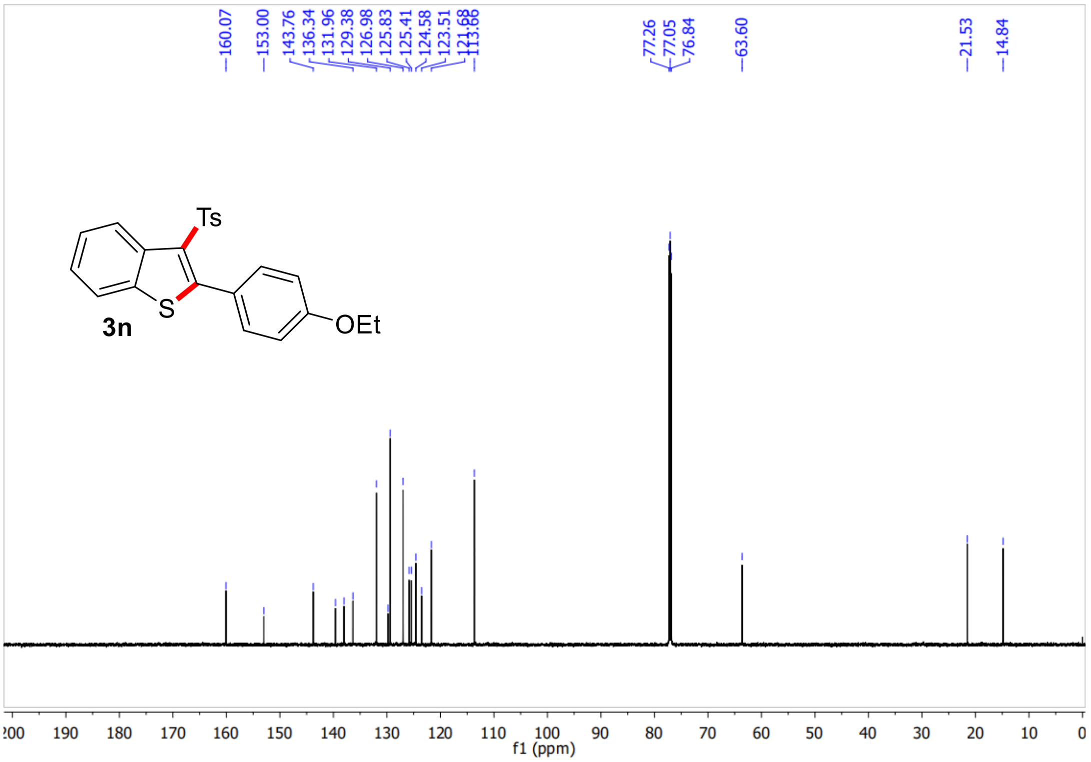
3n
Ts
S
OEt
f1 (ppm)

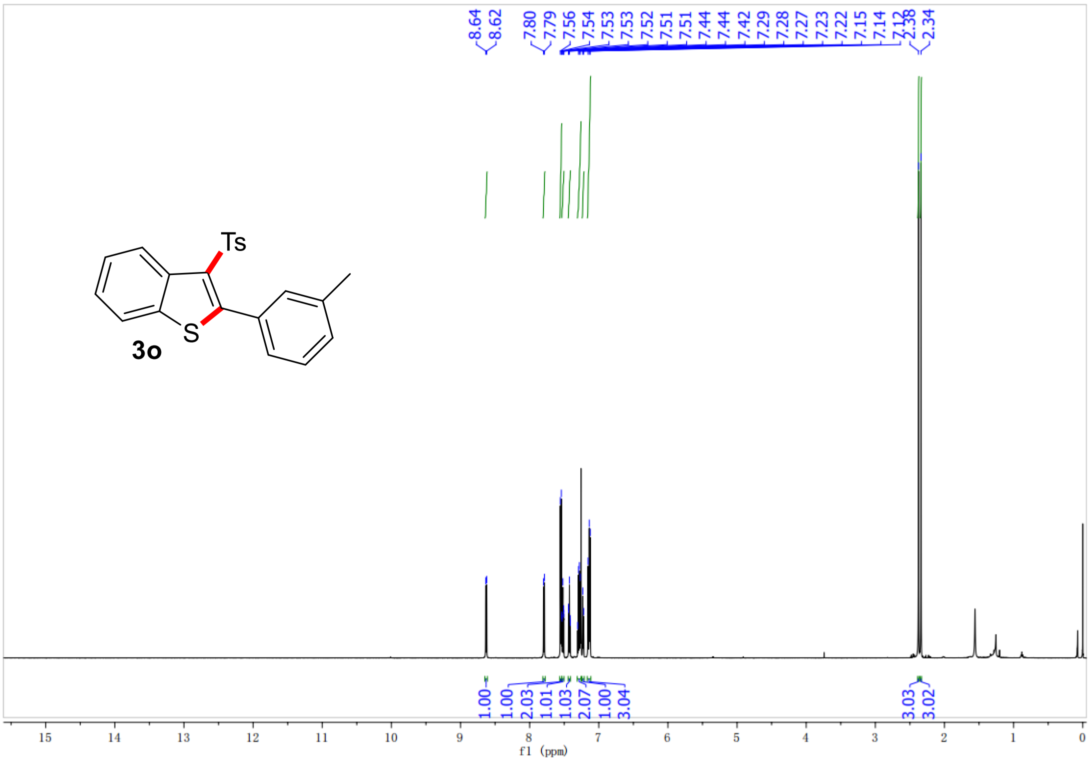
Ts
S
3o
8.64
8.62
7.80
7.79
7.56
7.54
7.53
7.53
7.52
7.51
7.51
7.44
7.44
7.42
7.29
7.28
7.27
7.23
7.22
7.15
7.14
7.12
2.38
2.34
1.00
1.00
2.03
1.01
1.03
2.07
1.00
3.04
3.03
3.02
f1 (ppm)

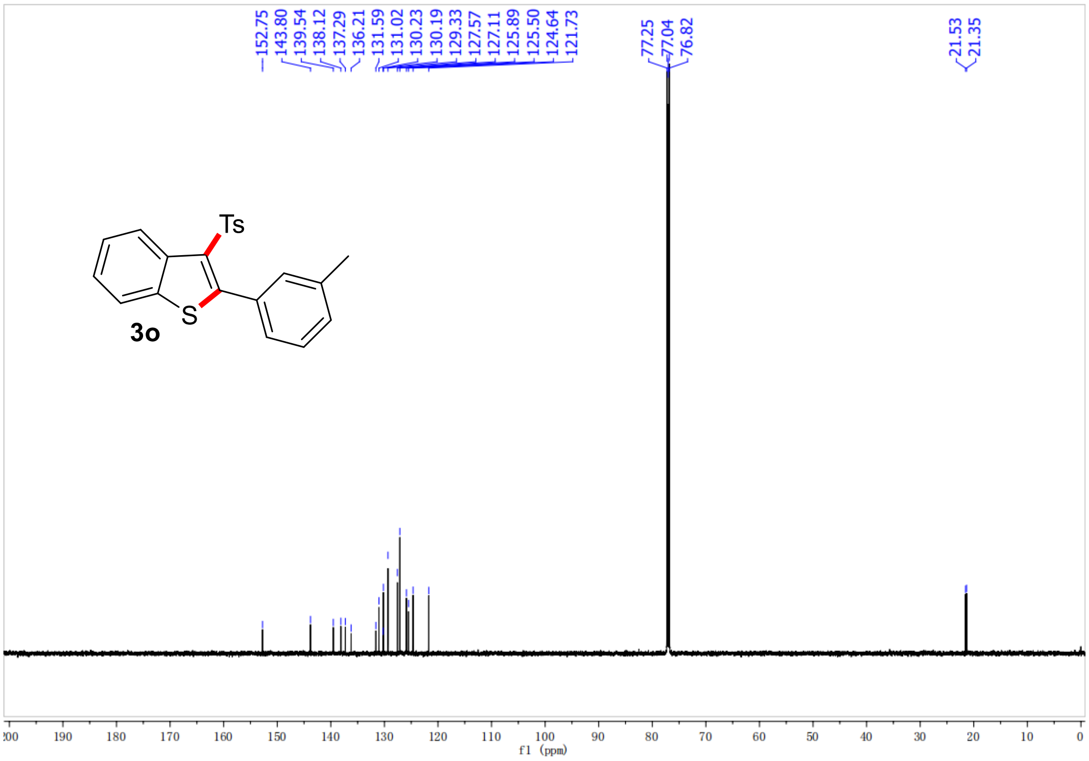
Ts
S
3o
152.75
143.80
139.54
138.12
137.29
136.21
131.59
131.02
130.23
130.19
129.33
127.57
127.11
125.89
125.50
124.64
121.73
77.25
77.04
76.82
21.53
21.35
f1 (ppm)

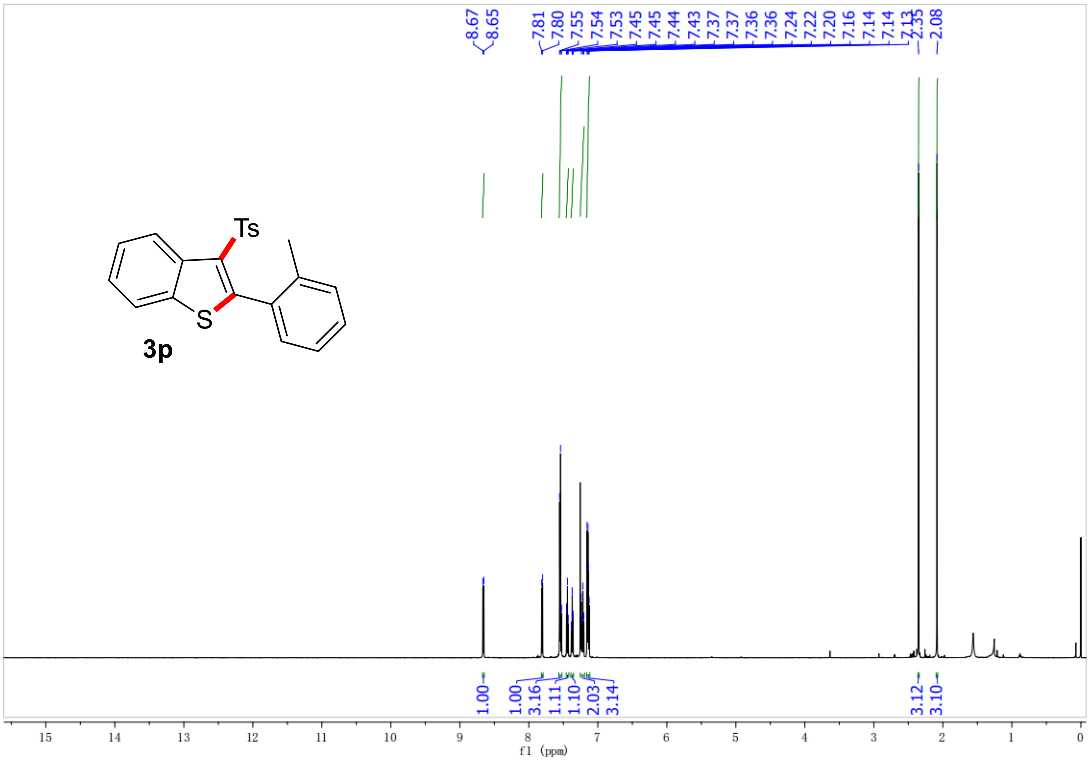

8.67
8.65
7.81
7.80
7.55
7.54
7.53
7.45
7.45
7.44
7.43
7.37
7.37
7.36
7.36
7.24
7.22
7.20
7.16
7.14
7.13
2.35
2.08
Ts
S
3p
1.00
1.00
3.16
1.11
1.10
2.03
3.14
3.12
3.10
f1 (ppm)

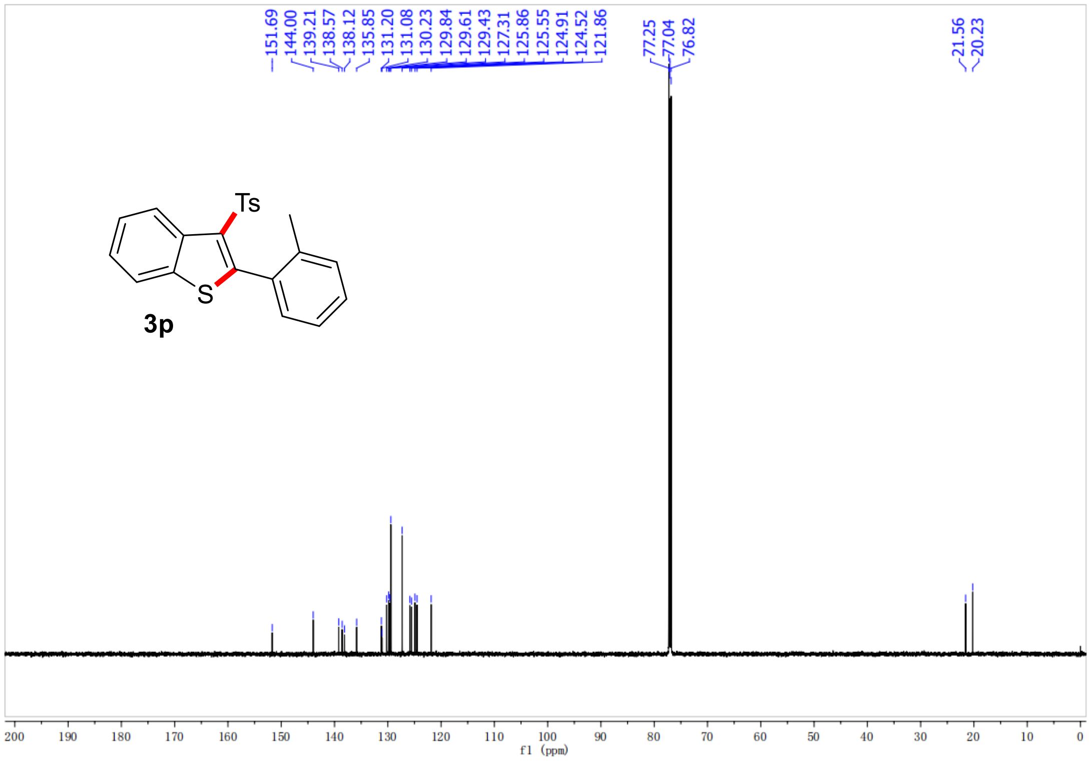

151.69
144.00
139.21
138.57
138.12
135.85
131.20
131.08
130.23
129.84
129.61
129.43
127.31
125.86
125.55
124.91
124.52
121.86
77.25
77.04
76.82
21.56
20.23
Ts
S
3p
f1 (ppm)

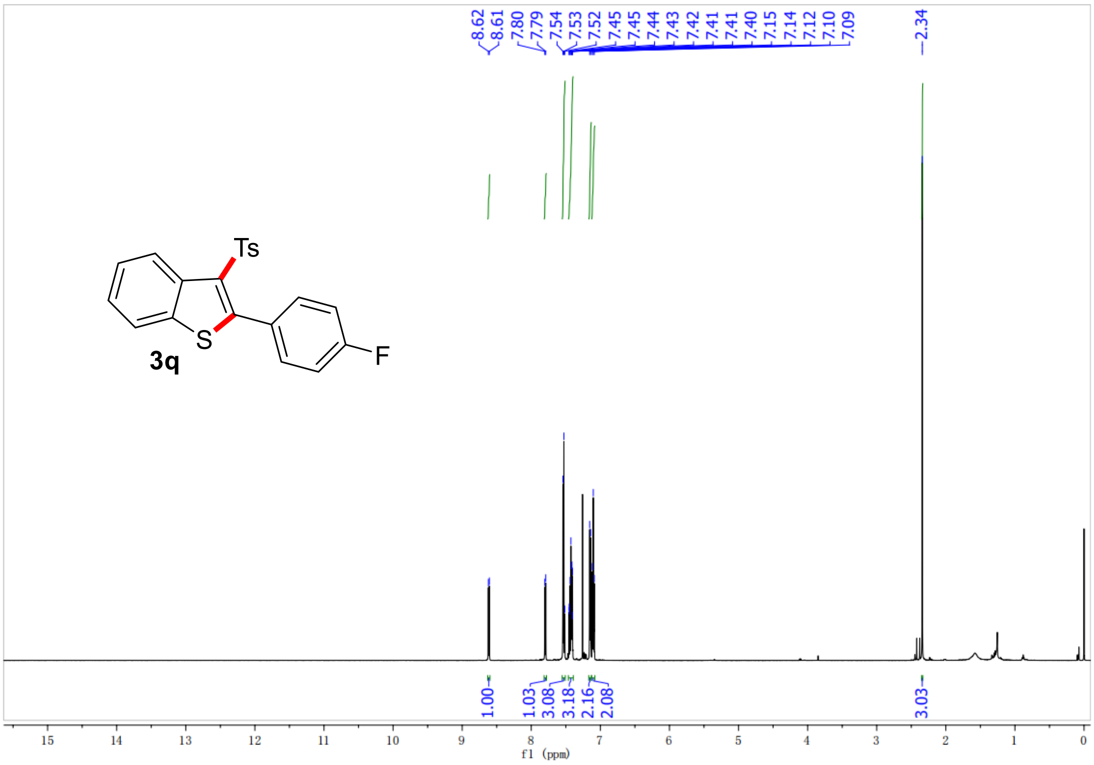

Ts
S
3q
F
8.62
8.61
7.80
7.79
7.54
7.53
7.52
7.45
7.45
7.44
7.43
7.42
7.41
7.41
7.40
7.15
7.14
7.12
7.10
7.09
2.34
1.00
1.03
3.08
3.18
2.16
2.08
3.03
15
14
13
12
11
10
9
8
7
6
5
4
3
2
1
0
f1 (ppm)

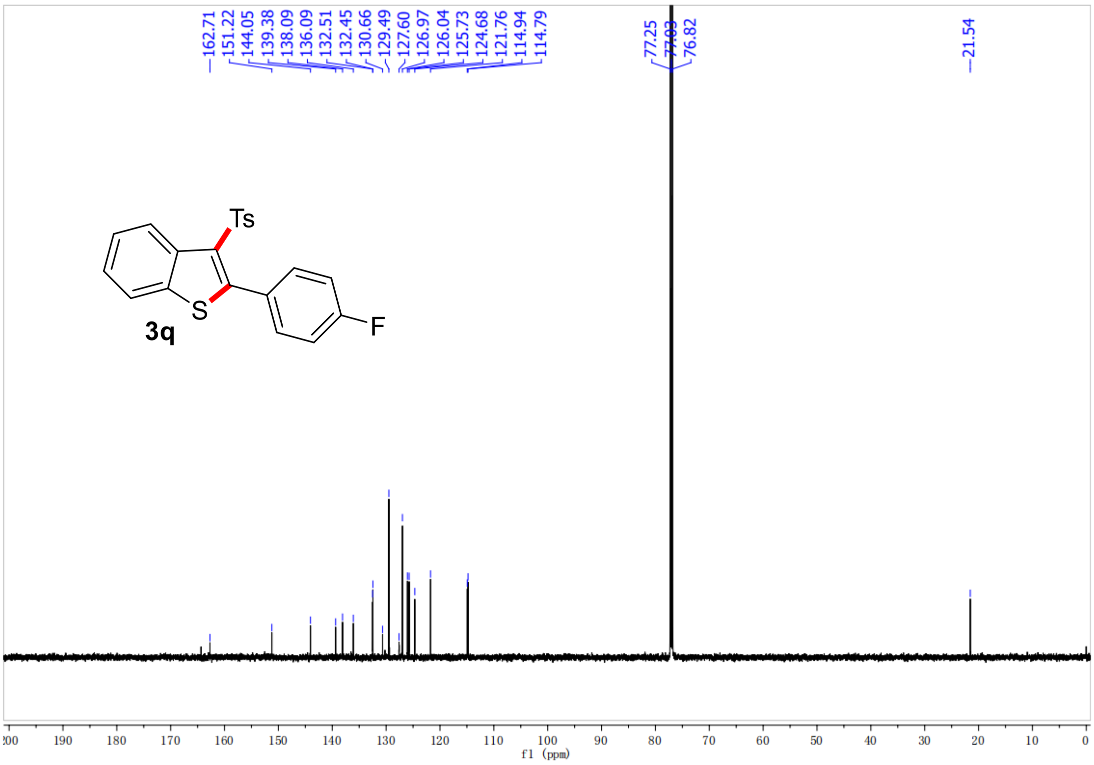

Ts
S
3q
F
162.71
151.22
144.05
139.38
138.09
136.09
132.51
132.45
130.66
129.49
127.60
126.97
126.04
125.73
124.68
121.76
114.94
114.79
77.25
77.03
76.82
21.54
200
190
180
170
160
150
140
130
120
110
100
90
80
70
60
50
40
30
20
10
0
f1 (ppm)

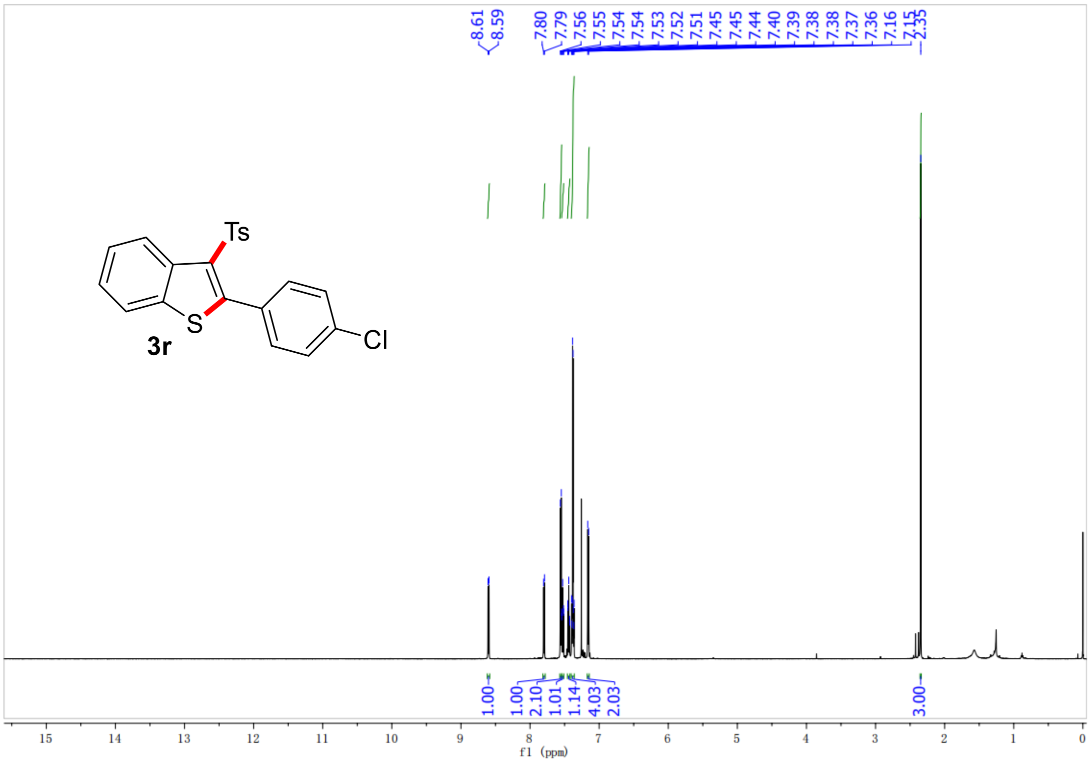
8.61
8.59
7.80
7.79
7.56
7.55
7.54
7.54
7.53
7.52
7.51
7.45
7.45
7.44
7.40
7.39
7.38
7.38
7.37
7.36
7.16
7.15
2.35
Ts
S
Cl
3r
1.00
1.00
2.10
1.01
1.14
4.03
2.03
3.00
15
14
13
12
11
10
9
8
7
6
5
4
3
2
1
0
f1 (ppm)

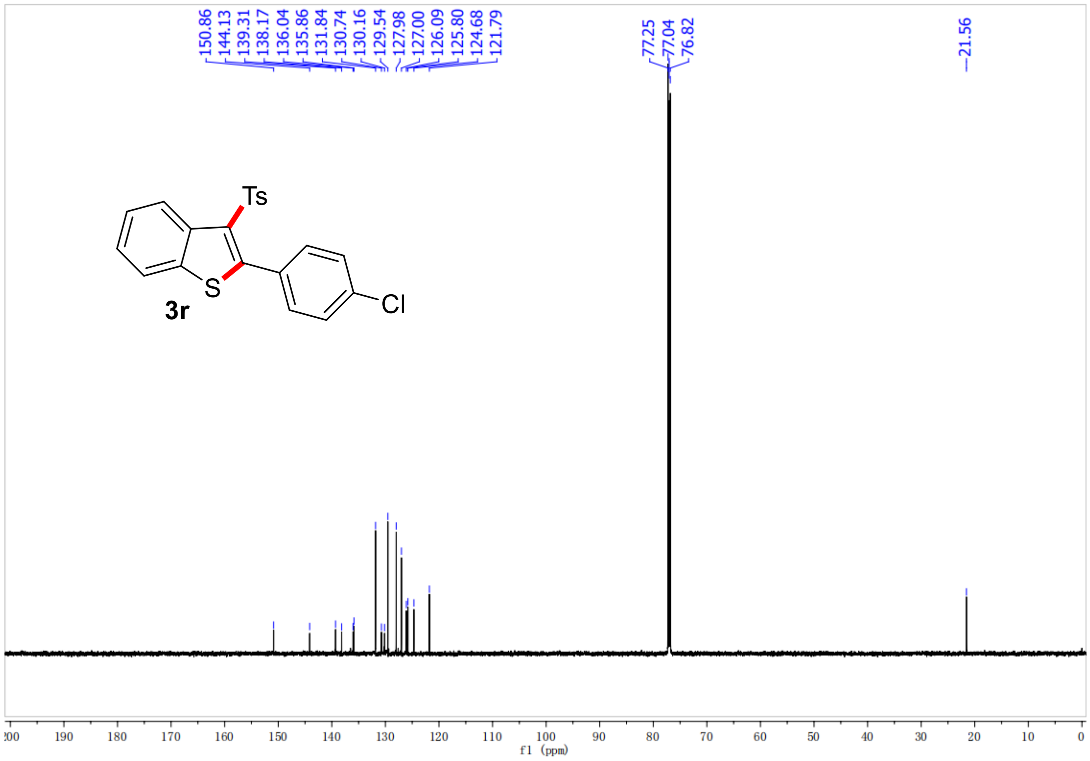
150.86
144.13
139.31
138.17
136.04
135.86
131.84
130.74
130.16
129.54
127.98
127.00
126.09
125.80
124.68
121.79
77.25
77.04
76.82
21.56
Ts
S
Cl
3r
200
190
180
170
160
150
140
130
120
110
100
90
80
70
60
50
40
30
20
10
0
f1 (ppm)

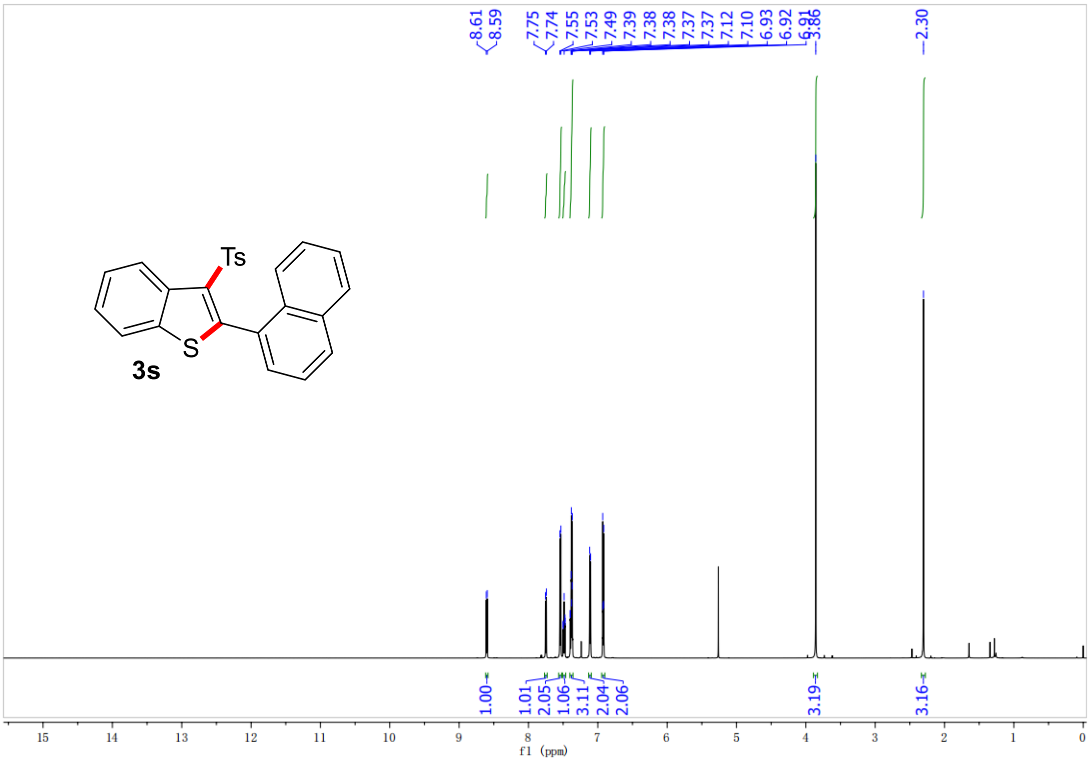
Ts
S
3s
8.61
8.59
7.75
7.74
7.55
7.53
7.49
7.39
7.38
7.38
7.37
7.37
7.12
7.10
6.93
6.92
3.86
2.30
1.00
1.01
2.05
1.06
3.11
2.04
2.06
3.19
3.16
f1 (ppm)

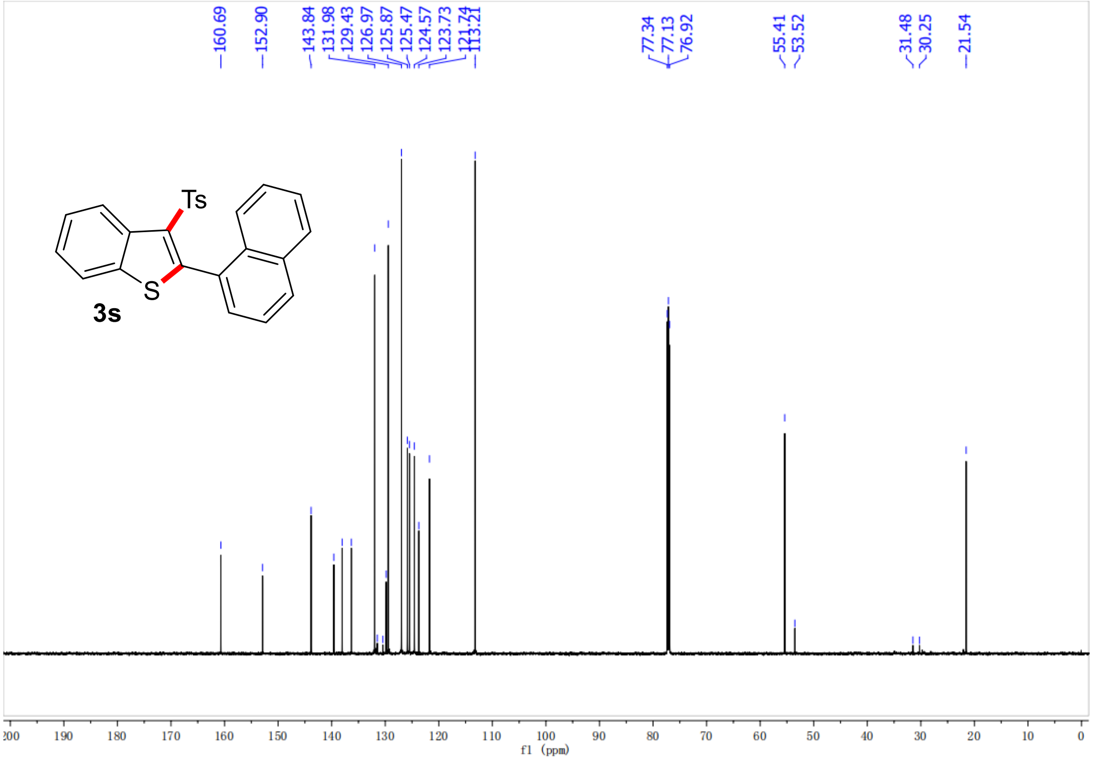
Ts
S
3s
160.69
152.90
143.84
131.98
129.43
126.97
125.87
125.47
124.57
123.73
121.74
113.21
77.34
77.13
76.92
55.41
53.52
31.48
30.25
21.54
f1 (ppm)

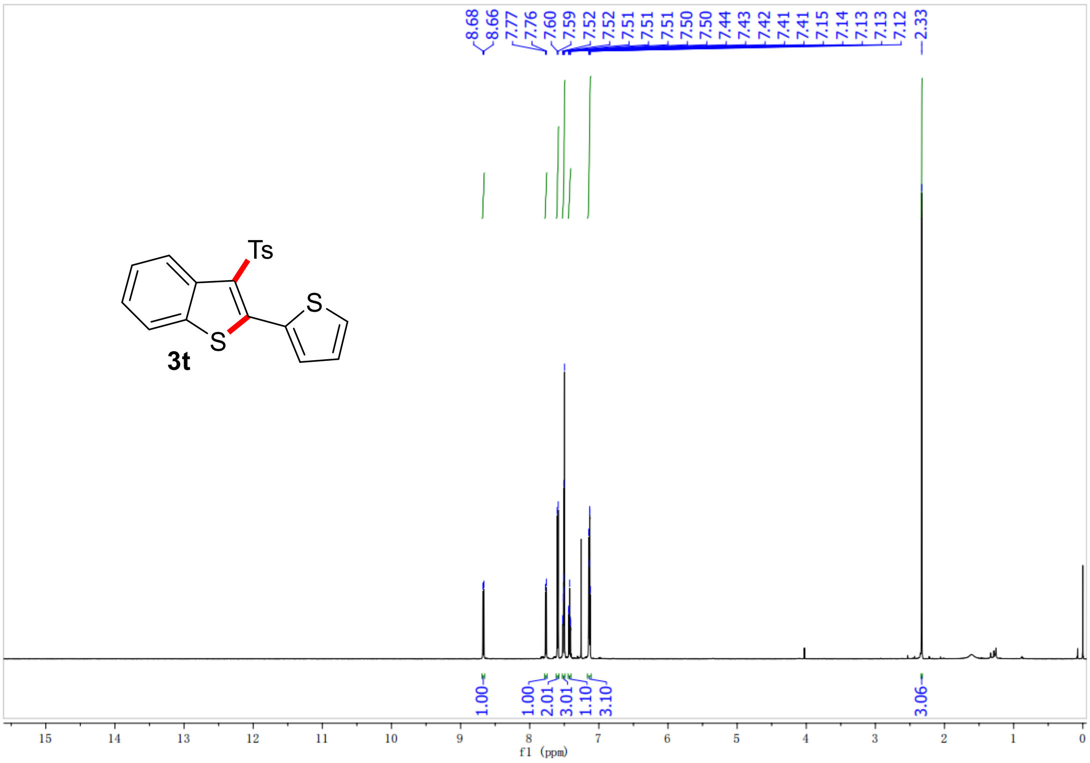
8.68
8.66
7.77
7.76
7.60
7.59
7.52
7.52
7.51
7.51
7.51
7.50
7.50
7.44
7.43
7.42
7.41
7.41
7.15
7.14
7.13
7.13
7.12
2.33
Ts
S
S
3t
1.00
1.00
2.01
3.01
1.10
3.10
3.06
f1 (ppm)

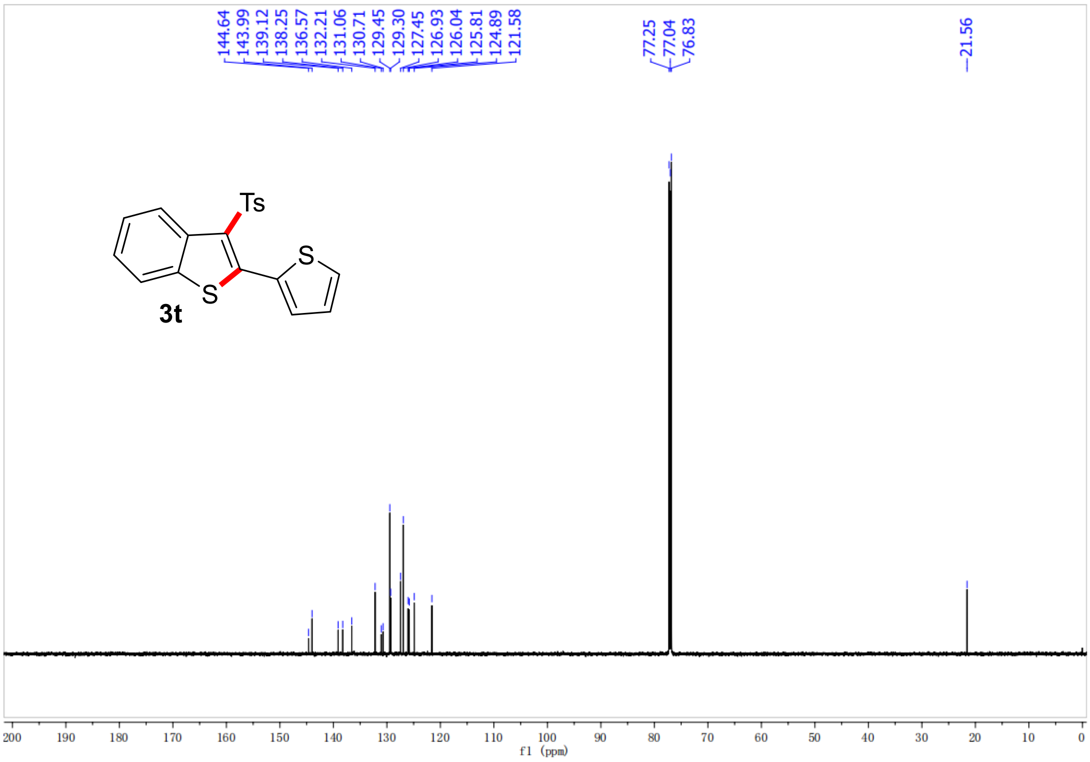
144.64
143.99
139.12
138.25
136.57
132.21
131.06
130.71
129.45
129.30
127.45
126.93
126.04
125.81
124.89
121.58
77.25
77.04
76.83
21.56
Ts
S
S
3t
f1 (ppm)

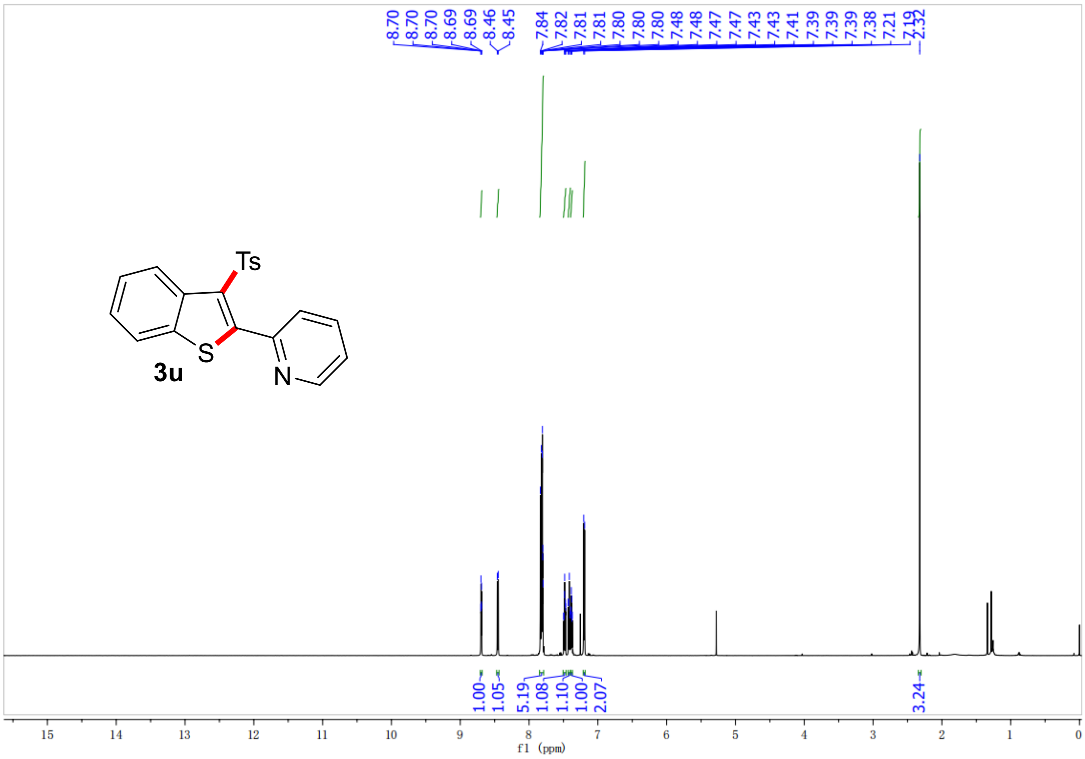

8.70
8.70
8.70
8.69
8.69
8.46
8.45
7.84
7.82
7.81
7.81
7.80
7.80
7.80
7.48
7.48
7.47
7.47
7.43
7.43
7.41
7.39
7.39
7.39
7.38
7.21
7.19
2.32
Ts
S
N
3u
1.00
1.05
5.19
1.08
1.10
1.00
2.07
3.24
f1 (ppm)

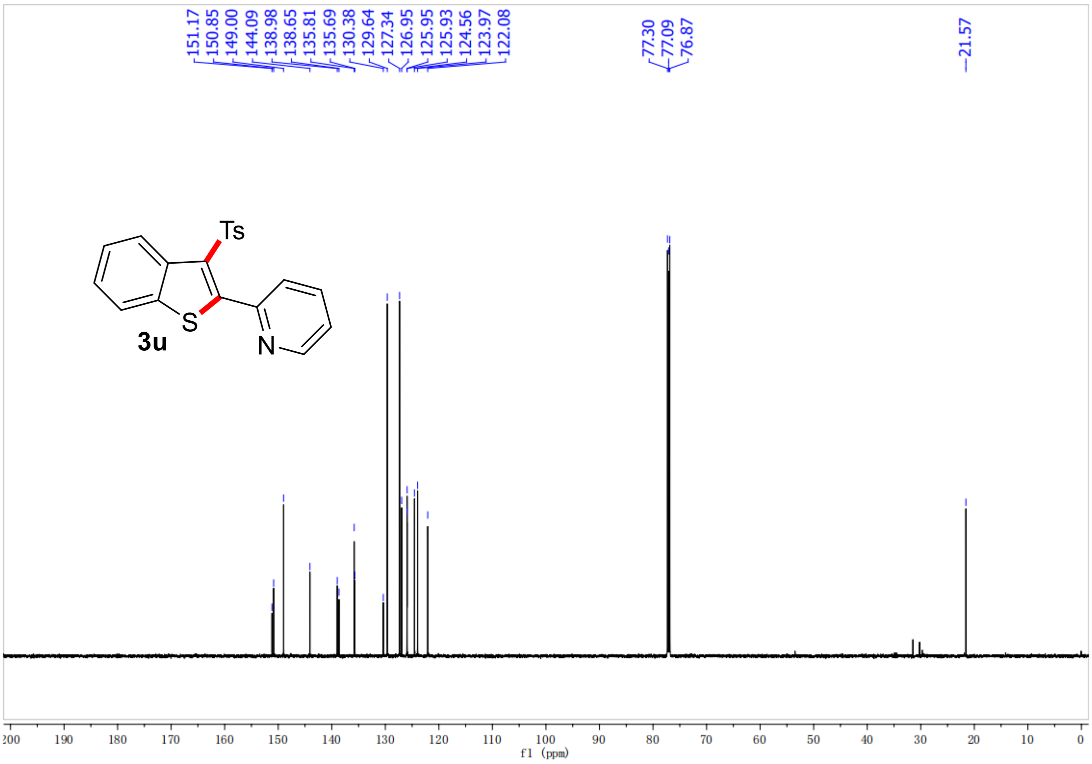

151.17
150.85
149.00
144.09
138.98
138.65
135.81
135.69
130.38
129.64
127.34
126.95
125.95
125.93
124.56
123.97
122.08
77.30
77.09
76.87
21.57
Ts
S
N
3u
f1 (ppm)

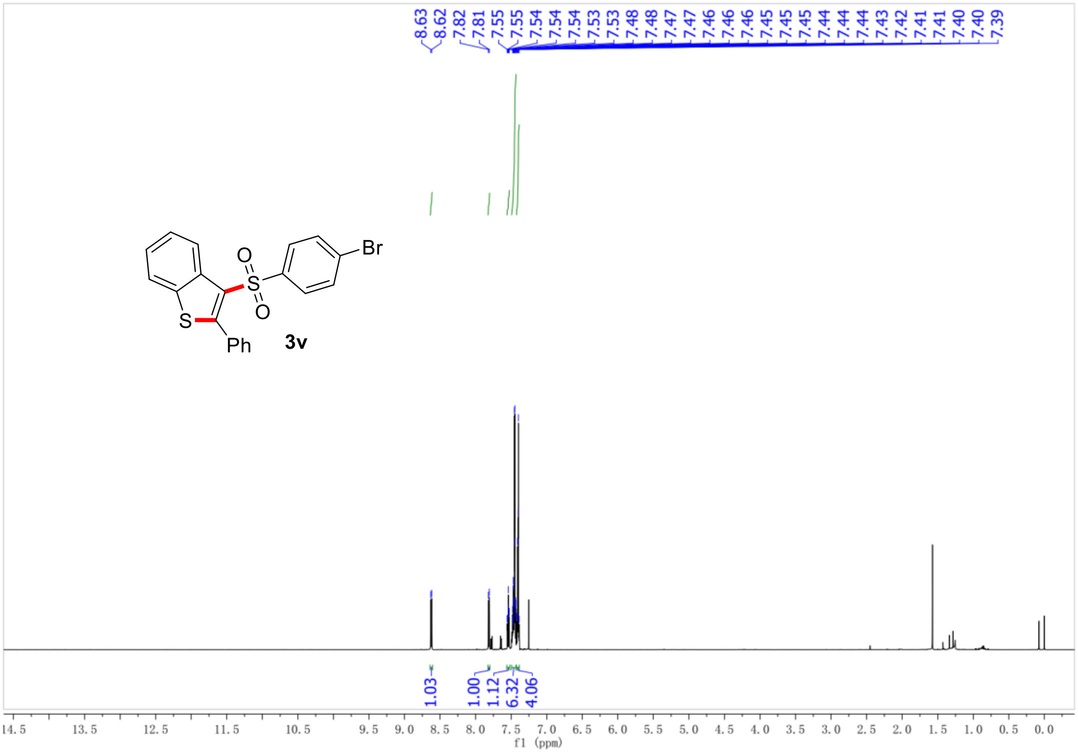
8.63
8.62
7.82
7.81
7.55
7.55
7.54
7.54
7.54
7.53
7.53
7.48
7.48
7.47
7.47
7.46
7.46
7.45
7.45
7.45
7.44
7.44
7.44
7.43
7.42
7.41
7.40
7.40
7.39
Br
O
S
O
S
Ph
3v
1.03
1.00
1.12
6.32
4.06
f1 (ppm)

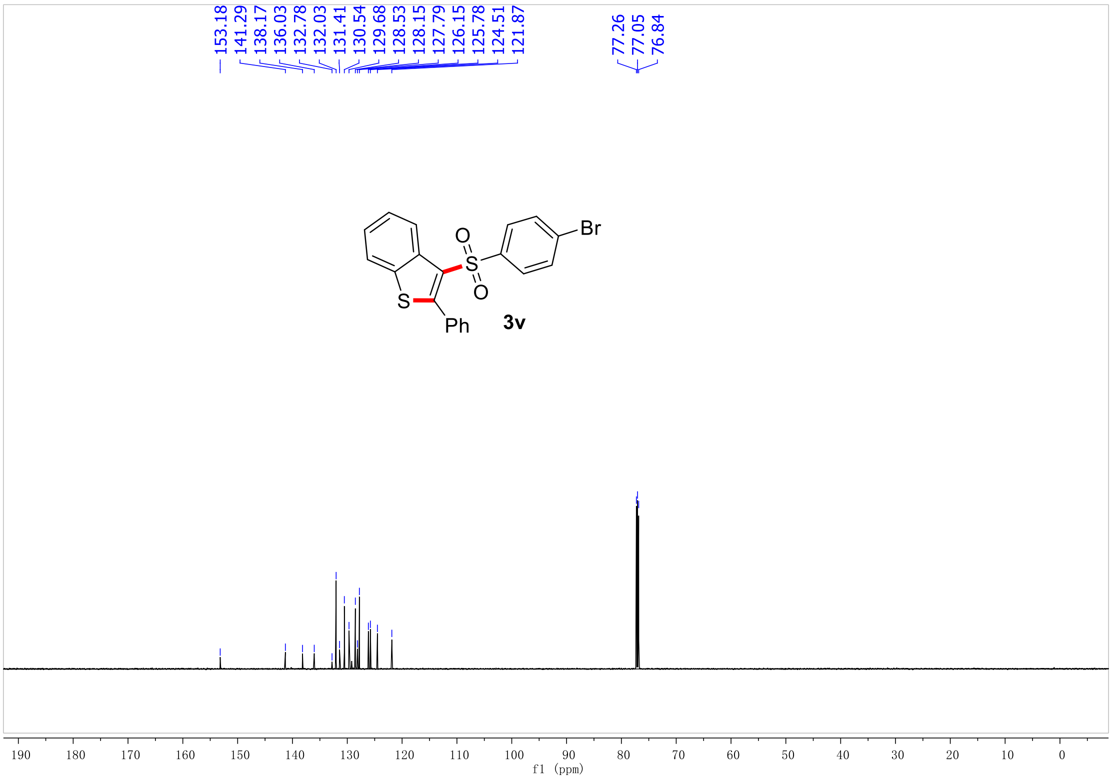
153.18
141.29
138.17
136.03
132.78
132.03
131.41
130.54
129.68
128.53
128.15
127.79
126.15
125.78
124.51
121.87
77.26
77.05
76.84
Br
O
S
O
S
Ph
3v
f1 (ppm)

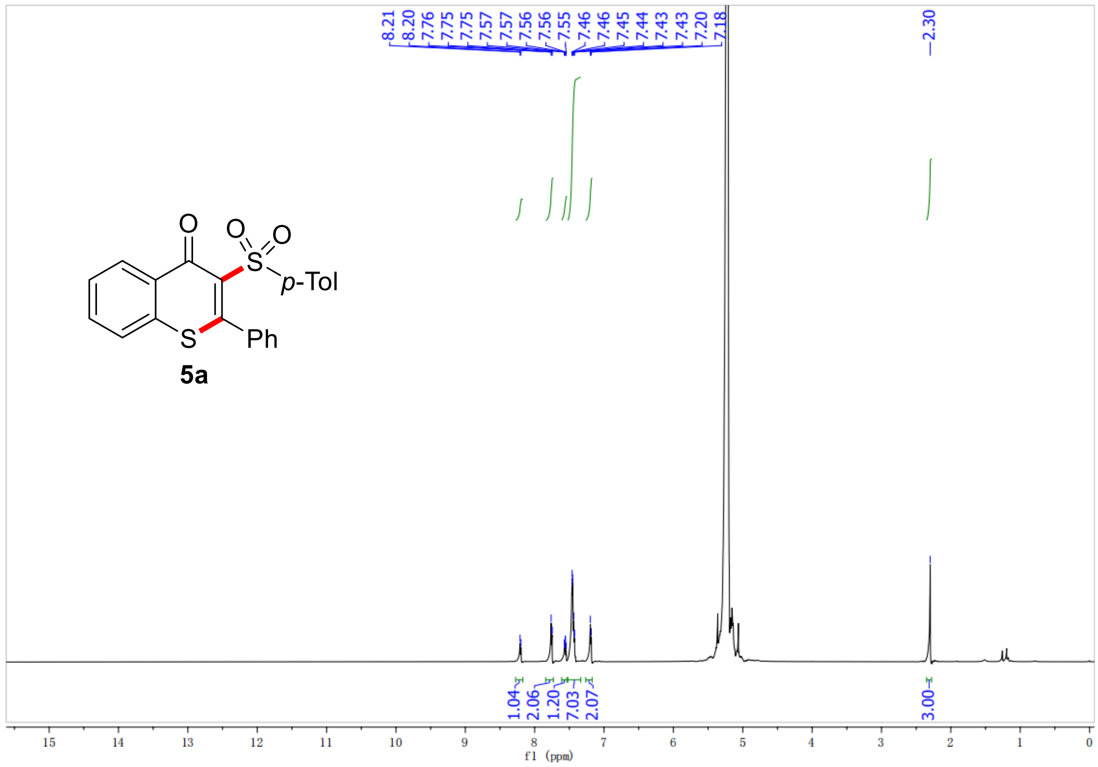
8.21
8.20
7.76
7.75
7.75
7.57
7.57
7.56
7.56
7.55
7.46
7.46
7.45
7.44
7.43
7.43
7.20
7.18
2.30
5a
p-Tol
Ph
1.04
2.06
1.20
7.03
2.07
3.00
f1 (ppm)

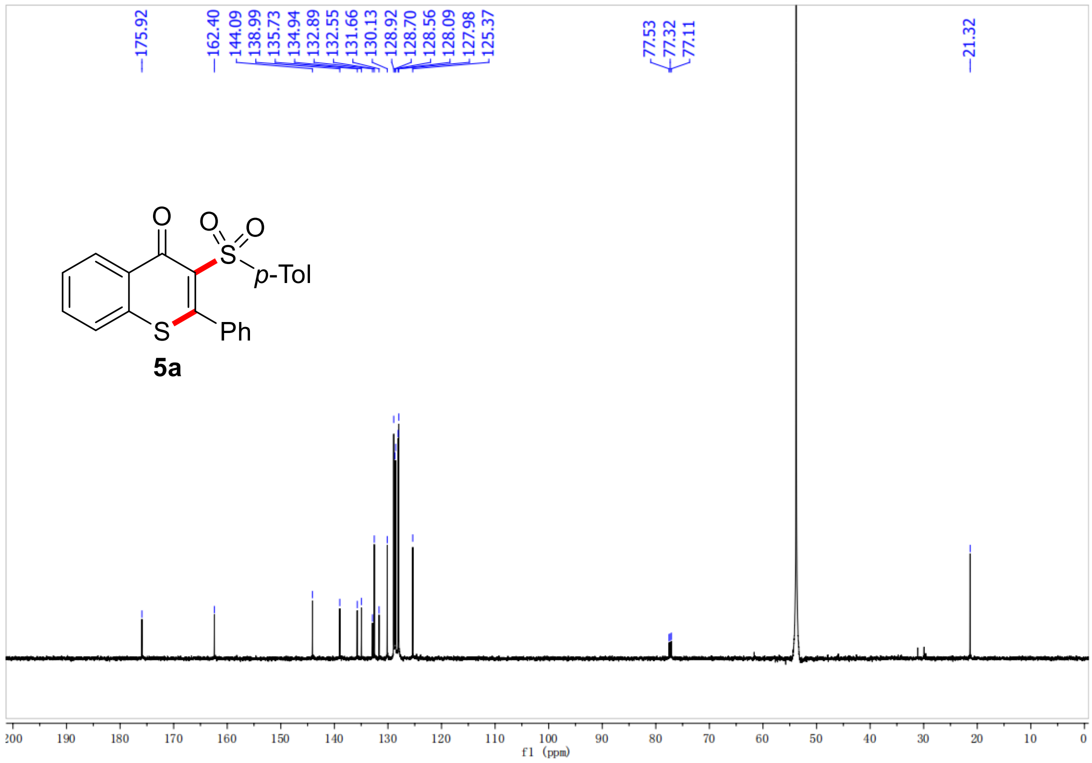
175.92
162.40
144.09
138.99
135.73
134.94
132.89
132.55
131.66
130.13
128.92
128.70
128.56
128.09
127.98
125.37
77.53
77.32
77.11
21.32
5a
p-Tol
Ph
f1 (ppm)

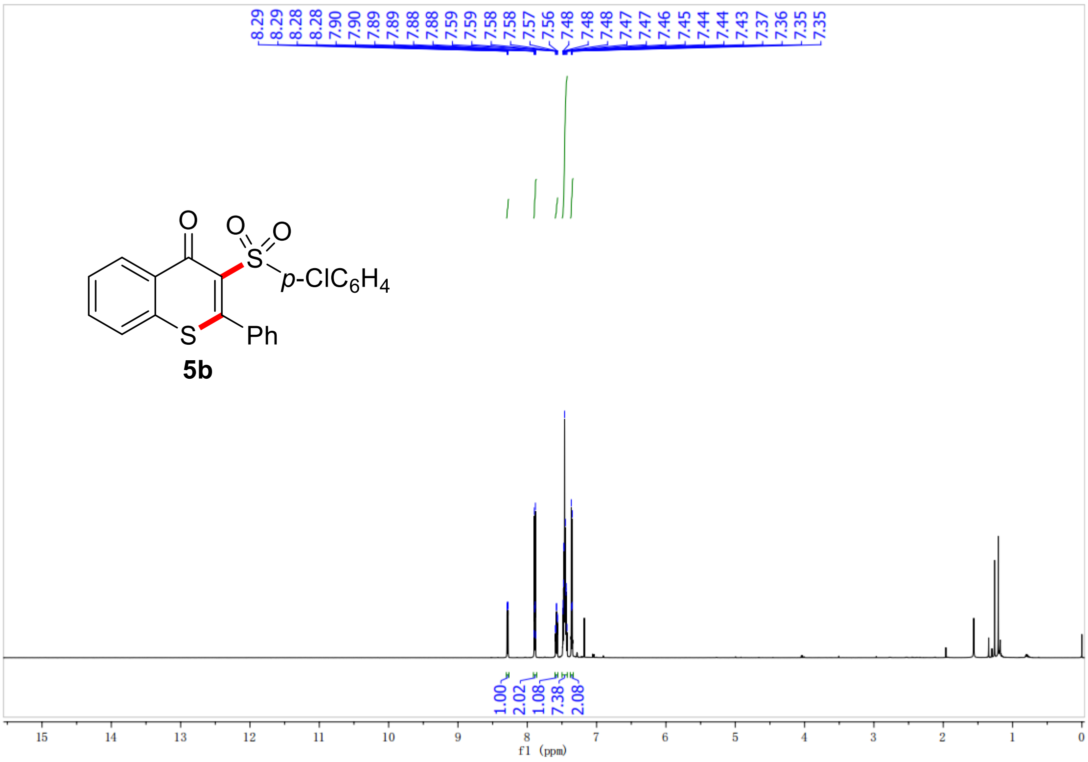
8.29
8.29
8.28
8.28
7.90
7.90
7.89
7.89
7.88
7.88
7.59
7.59
7.58
7.58
7.57
7.56
7.48
7.48
7.48
7.47
7.46
7.45
7.44
7.44
7.43
7.37
7.36
7.35
7.35
p-ClC6H4
Ph
O
S
5b
1.00
2.02
1.08
7.38
2.08
f1 (ppm)

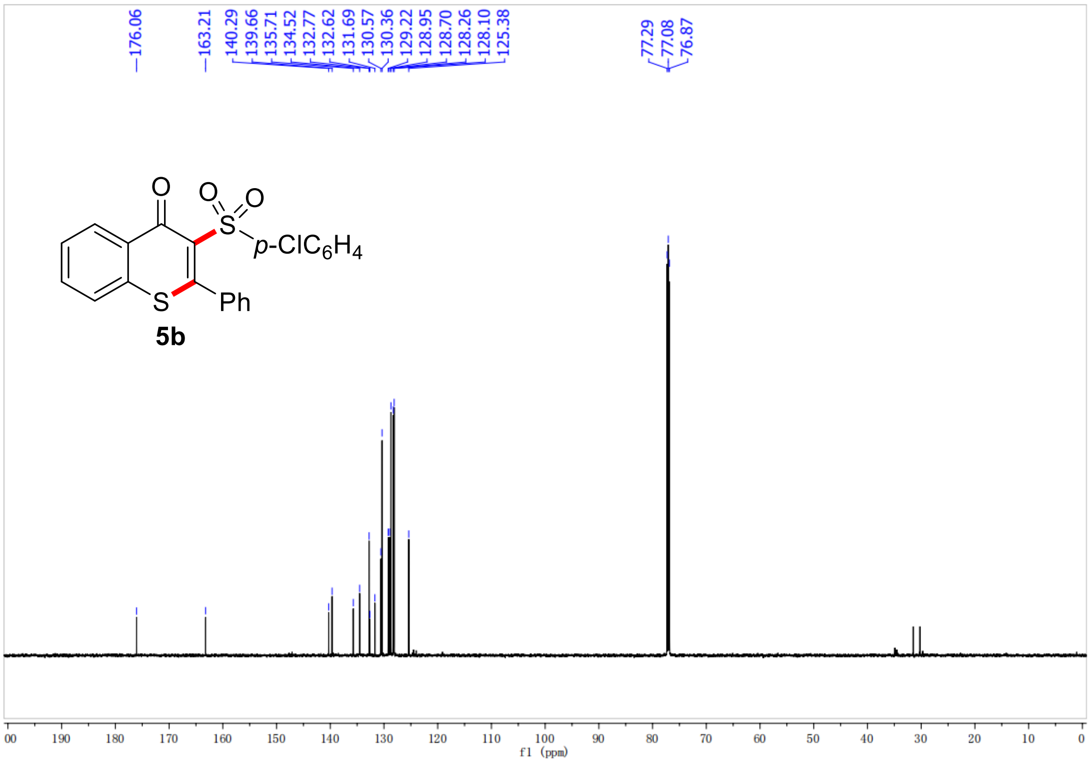
176.06
163.21
140.29
139.66
135.71
134.52
132.77
132.62
131.69
130.57
130.36
129.22
128.95
128.70
128.26
128.10
125.38
77.29
77.08
76.87
p-ClC6H4
Ph
5b
f1 (ppm)

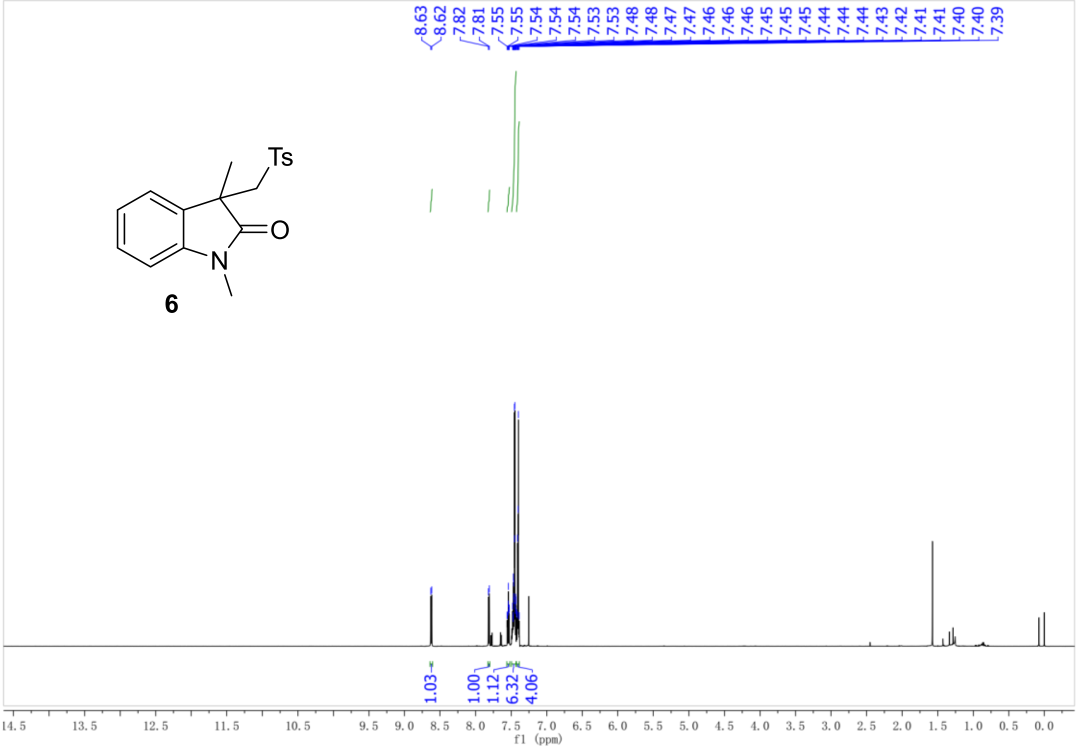
8.63
8.62
7.82
7.81
7.55
7.55
7.54
7.54
7.54
7.53
7.53
7.48
7.48
7.47
7.46
7.46
7.45
7.45
7.45
7.44
7.44
7.44
7.43
7.42
7.41
7.41
7.40
7.40
7.39
Ts
O
N
6
1.03
1.00
1.12
6.32
4.06
f1 (ppm)

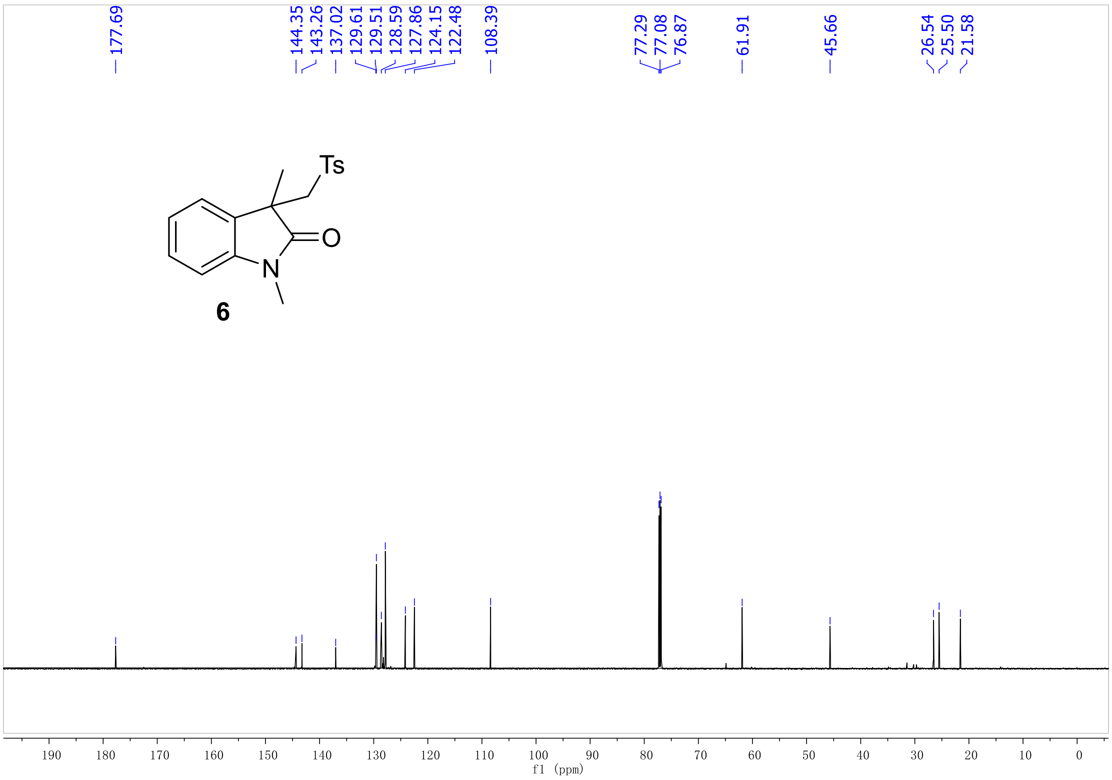
177.69
144.35
143.26
137.02
129.61
129.51
128.59
127.86
124.15
122.48
108.39
77.29
77.08
76.87
61.91
45.66
26.54
25.50
21.58
Ts
O
N
6
f1 (ppm)
